# Mitochondria function associated genes contribute to Parkinson’s Disease risk and later age at onset

**DOI:** 10.1038/s41531-019-0080-x

**Published:** 2019-05-22

**Authors:** Kimberley J. Billingsley, Ines A. Barbosa, Sara Bandrés-Ciga, John P. Quinn, Vivien J. Bubb, Charu Deshpande, Juan A. Botia, Regina H. Reynolds, David Zhang, Michael A. Simpson, Cornelis Blauwendraat, Ziv Gan-Or, J. Raphael Gibbs, Mike A. Nalls, Andrew Singleton, International Parkinson’s Disease Genomics Consortium (IPDGC), A. Noyce, A. Tucci, B. Middlehurst, D. Kia, M. Tan, H. Houlden, H. R. Morris, H. Plun-Favreau, P. Holmans, J. Hardy, D. Trabzuni, J. Bras, K. Mok, K. Kinghorn, N. Wood, P. Lewis, R. Guerreiro, R. Lovering, L. R’Bibo, M. Rizig, V. Escott-Price, V. Chelban, T. Foltynie, N. Williams, A. Brice, F. Danjou, S. Lesage, M. Martinez, A. Giri, C. Schulte, K. Brockmann, J. Simón-Sánchez, P. Heutink, P. Rizzu, M. Sharma, T. Gasser, A. Nicolas, M. Cookson, F. Faghri, D. Hernandez, J. Shulman, L. Robak, S. Lubbe, S. Finkbeiner, N. Mencacci, C. Lungu, S. Scholz, X. Reed, H. Leonard, G. Rouleau, L. Krohan, J. van Hilten, J. Marinus, A. Adarmes-Gómez, M. Aguilar, I. Alvarez, V. Alvarez, F. Javier Barrero, J. Bergareche Yarza, I. Bernal-Bernal, M. Blazquez, M. Bonilla-Toribio Bernal, M. Boungiorno, Dolores Buiza-Rueda, A. Cámara, M. Carcel, F. Carrillo, M. Carrión-Claro, D. Cerdan, J. Clarimón, Y. Compta, M. Diez-Fairen, O. Dols-Icardo, J. Duarte, R. l. Duran, F. Escamilla-Sevilla, M. Ezquerra, M. Fernández, R. Fernández-Santiago, C. Garcia, P. García-Ruiz, P. Gómez-Garre, M. Gomez Heredia, I. Gonzalez-Aramburu, A. Gorostidi Pagola, J. Hoenicka, J. Infante, S. Jesús, A. Jimenez-Escrig, J. Kulisevsky, M. Labrador-Espinosa, J. Lopez-Sendon, A. López de Munain Arregui, D. Macias, I. Martínez Torres, J. Marín, M. Jose Marti, J. Martínez-Castrillo, C. Méndez-del-Barrio, M. Menéndez González, A. Mínguez, P. Mir, E. Mondragon Rezola, E. Muñoz, J. Pagonabarraga, P. Pastor, F. Perez Errazquin, T. Periñán-Tocino, J. Ruiz-Martínez, C. Ruz, A. Sanchez Rodriguez, M. Sierra, E. Suarez-Sanmartin, C. Tabernero, J. Pablo Tartari, C. Tejera-Parrado, E. Tolosa, F. Valldeoriola, L. Vargas-González, L. Vela, F. Vives, A. Zimprich, L. Pihlstrom, P. Taba, K. Majamaa, A. Siitonen, N. Okubadejo, O. Ojo, Mina Ryten, Sulev Koks

**Affiliations:** 1 Department of Molecular and Clinical Pharmacology, Institute of Translational Medicine, , University of Liverpool, Liverpool, UK; 2 Laboratory of Neurogenetics, National Institute on Aging, National Institutes of Health, Bethesda, MD 20892 USA; 3 Department of Medical and Molecular Genetics, King’s College London School of Basic and Medical Biosciences, London, SE1 9RT UK; 4 Clinical Genetics Unit, Guys and St. Thomas’ NHS Foundation Trust, London, SE1 9RT UK; 5 Departamento de Ingeniería de la Información y las Comunicaciones, Universidad de Murcia, 30100 Murcia, Spain; 6 Department of Neurodegenerative Disease, UCL Institute of Neurology, 10-12 Russell Square House, London, UK; 7 Montreal Neurological Institute, McGill University, Montréal, QC Canada; 8 Department of Neurology and Neurosurgery, McGill University, Montréal, QC Canada; 9 Department of Human Genetics, McGill University, Montréal, QC Canada; 10 Data Tecnica International, Glen Echo, MD 20812 USA; 11 The Perron Institute for Neurological and Translational Science, 8 Verdun Street, Nedlands, WA 6009 Australia; 12 Centre for Comparative Genomics, Murdoch University, Murdoch, 6150 Australia; 13 Preventive Neurology Unit, Wolfson Institute of Preventive Medicine, QMUL, London, UK; 14 Department of Molecular Neuroscience, UCL Institute of Neurology, London, UK; 15 UCL Genetics Institute; and Department of Molecular Neuroscience, UCL Institute of Neurology, London, UK; 16 Department of Clinical Neuroscience, University College London, London, UK; 17 Biostatistics & Bioinformatics Unit, Institute of Psychological Medicine and Clinical Neuroscience, MRC Centre for Neuropsychiatric Genetics & Genomics, Cardiff, UK; 18 Department of Genetics, King Faisal Specialist Hospital and Research Centre, Riyadh, 11211 Saudi Arabia; 19 UK Dementia Research Institute at UCL and Department of Molecular Neuroscience, UCL Institute of Neurology, London, UK; 20 Institute of Healthy Ageing, University College London, London, UK; 21 University of Reading, Reading, UK; 22 University College London, London, UK; 23 MRC Centre for Neuropsychiatric Genetics and Genomics, Cardiff, UK; 24 Cardiff University School of Medicine, Cardiff, UK; 25 Institut du Cerveau et de la Moelle épinière, ICM, Inserm U 1127, CNRS, UMR 7225, Sorbonne Universités, UPMC University Paris 06, UMR S 1127, AP-HP, Pitié-Salpêtrière Hospital, Paris, France; 26 INSERM UMR 1220; and Paul Sabatier University, Toulouse, France; 27 Department for Neurodegenerative Diseases, Hertie Institute for Clinical Brain Research, University of Tübingen, Tübingen, Germany; 28 DZNE, German Center for Neurodegenerative Diseases, Tübingen, Germany; 29 Centre for Genetic Epidemiology, Institute for Clinical Epidemiology and Applied Biometry, University of Tubingen, Tubingen, Germany; 30 Department of Computer Science, University of Illinois at Urbana-Champaign, Urbana, IL USA; 31 Departments of Neurology, Neuroscience, and Molecular & Human Genetics, Baylor College of Medicine, Houston, TX USA; 32 Jan and Dan Duncan Neurological Research Institute, Texas Children’s Hospital, Houston, TX USA; 33 Baylor College of Medicine, Houston, TX USA; 34 Ken and Ruth Davee Department of Neurology, Northwestern University Feinberg School of Medicine, Chicago, IL USA; 35 Departments of Neurology and Physiology, University of California, San Francisco, CA USA; 36 Gladstone Institute of Neurological Disease, San Francisco, CA USA; 37 Taube/Koret Center for Neurodegenerative Disease Research, San Francisco, CA USA; 38 Northwestern University Feinberg School of Medicine, Chicago, IL USA; 39 National Institutes of Health Division of Clinical Research, NINDS, National Institutes of Health, Bethesda, MD USA; 40 Neurodegenerative Diseases Research Unit, National Institute of Neurological Disorders and Stroke, Bethesda, MD USA; 41 Department of Human Genetics, McGill University, Montréal, QC H3A 0G4 Canada; 42 Department of Neurology, Leiden University Medical Center, Leiden, Netherlands; 43 Instituto de Biomedicina de Sevilla (IBiS), Hospital Universitario Virgen del Rocío/CSIC/Universidad de Sevilla, Seville, Spain; 44 Fundació Docència i Recerca Mútua de Terrassa and Movement Disorders Unit, Department of Neurology, University Hospital Mutua de Terrassa, Terrassa, Barcelona, Spain; 45 Hospital Universitario Central de Asturias, Oviedo, Spain; 46 Hospital Universitario Parque Tecnologico de la Salud, Granada, Spain; 47 Instituto de Investigación Sanitaria Biodonostia, San Sebastián, Spain; 48 Hospital Clinic de Barcelona, Barcelona, Spain; 49 Hospital General de Segovia, Segovia, Spain; 50 Memory Unit, Department of Neurology, IIB Sant Pau, Hospital de la Santa Creu i Sant Pau, Universitat Autònoma de Barcelona, Barcelona, Spain; 51 Centro de Investigación Biomédica en Red en Enfermedades Neurodegenerativas (CIBERNED), Madrid, Spain; 52 Centro de Investigacion Biomedica, Universidad de Granada, Granada, Spain; 53 Hospital Universitario Virgen de las Nieves, Instituto de Investigación Biosanitaria de Granada, Granada, Spain; 54 Instituto de Investigación Sanitaria Fundación Jiménez Díaz, Madrid, Spain; 55 Hospital Universitario Virgen de la Victoria, Malaga, Spain; 56 Hospital Universitario Marqués de Valdecilla-IDIVAL, Santander, Spain; 57 Instituto de Investigación Sanitaria Biodonostia, San Sebastián, Spain; 58 Institut de Recerca Sant Joan de Déu, Barcelona, Spain; 59 Hospital Universitario Ramón y Cajal Madrid, Madrid, Spain; 60 Movement Disorders Unit, Department of Neurology, IIB Sant Pau, Hospital de la Santa Creu i Sant Pau, Universitat Autònoma de Barcelona, Barcelona, Spain; 61 Department of Neurology, Instituto de Investigación Sanitaria La Fe, Hospital Universitario y Politécnico La Fe, Valencia, Spain; 62 Department of Neurology, Hospital Universitario Fundación Alcorcón, Madrid, Spain; 63 Department of Neurology, Medical University of Vienna, Vienna, Austria; 64 Department of Neurology, Oslo University Hospital, Oslo, Norway; 65 Department of Neurology and Neurosurgery, University of Tartu, Tartu, Estonia; 66 Institute of Clinical Medicine, Department of Neurology, University of Oulu, Oulu, Finland; 67 Department of Neurology and Medical Research Center, Oulu University Hospital, Oulu, Finland; 68 University of Lagos, Yaba, Lagos State Nigeria

**Keywords:** Medical genetics, Risk factors

## Abstract

Mitochondrial dysfunction has been implicated in the etiology of monogenic Parkinson’s disease (PD). Yet the role that mitochondrial processes play in the most common form of the disease; sporadic PD, is yet to be fully established. Here, we comprehensively assessed the role of mitochondrial function-associated genes in sporadic PD by leveraging improvements in the scale and analysis of PD GWAS data with recent advances in our understanding of the genetics of mitochondrial disease. We calculated a mitochondrial-specific polygenic risk score (PRS) and showed that cumulative small effect variants within both our primary and secondary gene lists are significantly associated with increased PD risk. We further reported that the PRS of the secondary mitochondrial gene list was significantly associated with later age at onset. Finally, to identify possible functional genomic associations we implemented Mendelian randomization, which showed that 14 of these mitochondrial function-associated genes showed functional consequence associated with PD risk. Further analysis suggested that the 14 identified genes are not only involved in mitophagy, but implicate new mitochondrial processes. Our data suggests that therapeutics targeting mitochondrial bioenergetics and proteostasis pathways distinct from mitophagy could be beneficial to treating the early stage of PD.

## Introduction

Parkinson’s disease (PD) is a progressive neurodegenerative movement disorder characterized pathologically by the death of dopaminergic neurons in the substantia nigra (SN) and aggregation of α-synuclein protein (encoded by *SNCA*), within intraneuronal inclusions called Lewy bodies. The majority of PD cases are apparently sporadic in nature (~95%). Aging is a major risk factor for disease and due to population ageing the prevalence of PD is predicted to increase rapidly, making the identification of therapeutic targets a high priority.^1–3^

Although there have been great advances in understanding both the genetic architecture and cellular processes involved in PD, the exact molecular mechanisms that underlie PD remain unknown.^1^ However, it is suggested that PD has a complex etiology, involving several molecular pathways, and understanding these specific pathways will be key to establishing mechanistic targets for therapeutic intervention. While several key pathways are currently being investigated, including autophagy, endocytosis, immune response, and lysosomal function,^4–7^ mitochondrial function was the first biological process to be associated with PD.^8,9^

An interest in mitochondrial function and PD began with the observation that exposure to the drug 1-methyl-4-phenyl-1,2,3,4-tetrahydropyridine (MPTP) can cause rapid parkinsonism and neuronal loss in the SN in humans, and that this is mediated through inhibition of complex I of the mitochondrial electron transport chain.^7,10,11^ Subsequent work suggested that individuals with sporadic PD have reduced complex I activity not only in the SN, but in other brain regions and peripheral tissues.^12^ Genetic studies focusing on monogenic forms of PD provided further support for the involvement of mitochondrial dysfunction in the disease. Pathogenic mutations that lead to autosomal recessive forms of PD have been reported in *PINK1*, *PARK2, PARK7*, *CHCHD2*, and *VPS13C* and the proteins they encode are all now known to be involved in the mitochondrial quality control system and in particular mitophagy.^13–16^

Therefore, in this paper, we aim to comprehensively assess the role of mitochondrial function in sporadic PD by leveraging improvements in the scale and analysis of PD genome-wide association study (GWAS) data with recent advances in our understanding of the genetics of mitochondrial disease. The availability of large-scale genome-wide association data in PD cases and the rapid identification of genetic lesions that underlie mitochondrial disease provide an opportunity to systematically assess the role of genetic variability in mitochondrial linked genes in the context of risk for PD.^17^ In this study we combine these new resources with current statistical tools, such as polygenic risk scoring and Mendelian randomization, to explore the role of mitochondrial function in both PD risk and age at onset of disease to obtain novel insights.

## Results

### Common variation within mitochondria function genes contributes to the heritable component of PD

**Fig. 1:**
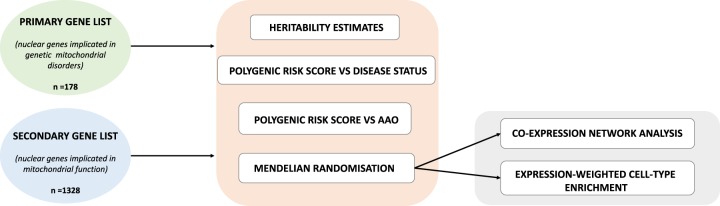
Workflow of mitochondrial-function specific PD analysis

The general workflow for the genetic analysis used in this study is shown in Fig. 1. First, to study the importance of mitochondrial function in sporadic PD, we investigated the heritability of PD specifically within genomic regions that contained genes annotated as important in mitochondrial function. The construction of this annotation was driven by the principle that genomic regions, which are known to be the sites of mutations in individuals with rare mitochondrial diseases or are candidate regions for such mutations provide the best evidence for involvement in mitochondrial function.

GCTA is a statistical method that estimates phenotypic variance of complex traits explained by genome-wide SNPs, including those not associated with the trait in a GWAS. Using GCTA, heritability estimates were first calculated for the four largest IPDGC GWAS datasets, including all variants (WTCCC PD GWAS (PMID:21044948), Spanish Parkinson’s (IPDGC) part2, NIA PD GWAS (PMID:19915575), Dutch GWAS (PMID:21248740). Owing to the low number of included cases, the heritability estimates in the other IPDGC datasets were deemed less reliable. Consistent with previous heritability estimates from both Keller et al.(24%) and Chang et al. (21%), our random-effects meta-analysis for the four datasets identified 23% (95% CI 12–34, *p* = 2.72E-05) phenotypic variance associated with all PD samples (Tables 1 and 2). There was a high degree of consistency across the cohorts.

**Table 1:** Cohort-level heritability analysis for the primary and secondary mitochondrial gene lists

	Primary		Secondary	
	Heritability estimate	SE of heritability estimate	Heritability estimate	SE of heritability estimate
WTCCC PD GWAS (PMID:21044948)	0.00321	0.00277	0.00563	0.00688
Spanish Parkinson’s (IPDGC) part2	0.00027	0.00314	0.00629	0.00932
NIA PD GWAS (PMID:19915575)	0.00945	0.00540	0.03616	0.01365
Dutch GWAS (PMID:21248740)	0.00000	0.00530	0.03562	0.01681

Reporting estimates for the WTCCC PD GWAS (PMID:21044948), Spanish Parkinson’s (IPDGC) part2, NIA PD GWAS (PMID:19915575), Dutch GWAS (PMID:21248740) cohorts. Showing heritability estimates generated using GCTA and standard error of the estimates (SE)

**Table 2:** Summary of random-effects meta-analysis for the primary and secondary mitochondrial gene lists

	Heritability estimate from random-effects	Lower 95% confidence interval	Upper 95% confidence interval	*p*-value from random effects	Heterogeneity of variance from random effects (%)	Heterogeneity *p*-value
All SNPs	0.2313	0.1233	0.3393	2.72E-05	0.0100	3.00E-03
Primary	0.0026	−0.0011	0.0062	1.66E-01	0.0000	4.85E-01
Secondary	0.0167	0.0007	0.0328	4.10E-02	0.0001	9.63E-02

Here, we show the random-effects meta-analysis of heritability estimates for all SNPs in the genome (All SNPs), estimate calculated with for the SNPs within the primary mitochondria list genes (Primary), and the SNPs within the secondary mitochondria list genes (Secondary)

After establishing the consistency of our heritability estimates, we next calculated heritability using only variants located within genic regions specified as being of primary (*n* = 176) or secondary (*n* = 1463) importance in mitochondrial function. Initially, to assess the full contribution of the mitochondria pathway we ran the analysis including and excluding the PD risk genes.^6^ However, as shown in Supplementary Fig. 1 there was little difference overall in the heritability estimates. Therefore, to only to catalog mitochondria-specific genetic risk outside of known loci we focused on the analysis excluding these genes. The heritability estimate using a random-effects meta-analysis for the primary gene list excluding the PD genes was estimated to be a modest 0.26% (95% CI −0.11–0.66, *p* = 0.166). However, the heritability estimate using a random-effects meta-analysis for the secondary list, namely genes implicated in mitochondrial function or morphology, as well as disease, was estimated to be 1.67% (95% CI −0.07–0.32, *p* = 0.041).

### Mitochondria function-specific polygenic risk score is significantly associated with disease status

Next, we calculated PRS to capture the addictive effect of all common variants within genes implicated in mitochondria function on PD risk. PRS is a particularly powerful approach in this context because it is able to efficiently incorporate information from all hits, including sub-significant hits, which may nonetheless be etiologically relevant. Again, initially we ran the analysis including and excluding the PD risk genes and the comparison can be seen in Supplementary Fig. 2. However, to only report novel associations we focused on the lists excluding the PD risk genes.

**Fig. 2:**
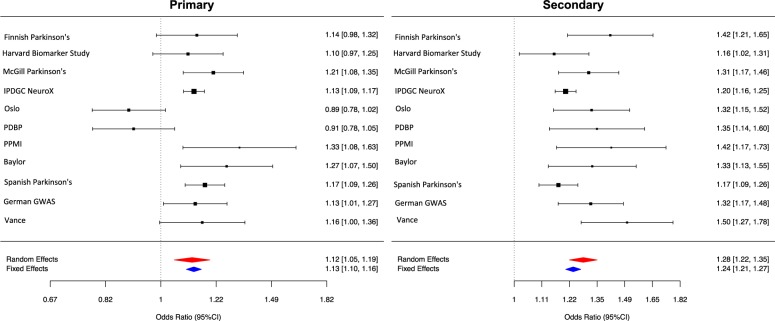
Forest plots of PRS for Parkinson’s Disease across cohorts. Random effect meta-analysis results are shown as red diamonds and fixed effects are shown as blue, with the centerline of each diamond representing the summary PRS for that dataset. IPDGC NeuroX = (Nalls et al. 2015, PMID:25444595), OSLO = Oslo Parkinson’s Disease Study, PDBP = Parkinson’s Disease Biomarker’s Program, PPMI = Parkinson’s Progression Markers Initiative, Baylor = Baylor College of Medicine/University of Maryland, German GWAS = (PMID:19915575), VANCE = Vance (dbGap phs000394)

Using this approach, the primary and secondary mitochondrial genomic annotations were found to be significantly associated with PD disease status. Remarkably, the primary gene list consisting of only 176 genes implicated in Mendelian mitochondrial disorders was associated with PD with an odds ratio of 1.12 per standard deviation increase in the PRS from the population mean (random-effects *p*-value = 6.00E-04, beta = 0.11, SE = 0.03). The secondary gene list, which also included genes implicated in mitochondria function or morphology, was associated with PD with a higher odds ratio of 1.28 per standard deviation increase in the PRS from the population mean (random-effects *p*-value = 1.9E-22, beta = 0.25, SE = 0.03) (Fig. 2). Altogether, these analyses not only provide further support for importance of mitochondrial processes in PD, but potentially provide a tool for identifying PD patients most likely to benefit from treatments specifically targeting mitochondrial function.

### Mitochondria function-specific polygenic risk score is significantly associated with later age at onset

**Fig. 3:**
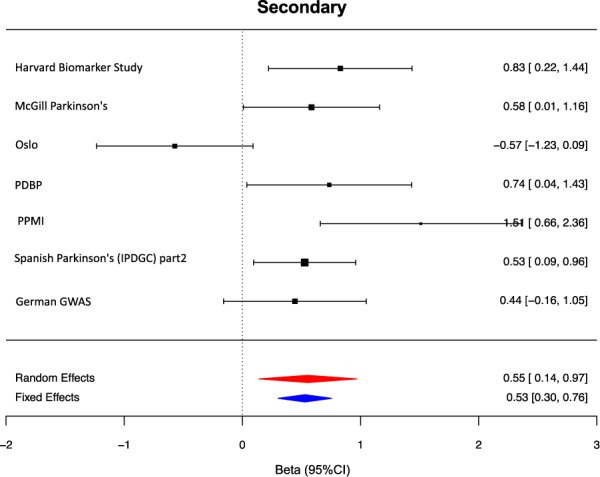
Forest plots of PRS for the age at onset of Parkinson’s Disease across cohorts. Random effect meta-analysis results are shown as red diamonds and fixed effects are shown as blue, with the centerline of each diamond representing the summary PRS for that dataset. OSLO = Oslo Parkinson’s Disease Study, PDBP = Parkinson’s Disease Biomarker’s Program, PPMI = Parkinson’s Progression Markers Initiative, German GWAS = (PMID:19915575), VANCE = Vance

Although multiple lines of evidence point to the importance of mitochondrial dysfunction as a primary cause of PD, impaired mitochondrial dynamics appears to be common to a wide range of neurodegenerative diseases, including Huntington’s disease,^18,19^ amyotrophic lateral sclerosis,^20,21^ and Alzheimer’s disease.^22–25^ The latter suggests that even when impaired mitochondrial function is not the primary event in disease pathogenesis, it is a common outcome and could contribute to disease progression. We sought to test this hypothesis by investigating the importance of common variation within our mitochondrial gene lists in determining the age at onset of PD (AAO). Given the significant lag period between PD pathophysiology and symptoms, AAO was used as an indirect measure of disease progression. This analysis was performed using PRS since it has been consistently found to be the main genetic predictor of AAO^6,26–28^ with higher genetic risk scores being significantly associated with an overall trend for earlier AAO of disease. While the primary mitochondrial gene list was not significantly associated with AAO of disease, the secondary gene list was correlated with AAO. Contrary to expectation, the cumulative burden of common variants within the 1326 genes comprising the PRS for PD risk were positively correlated with AAO of PD. After meta-analyzing, we found that each 1SD increase in PRS, led to a 0.55 year increase in the AAO of disease (summary effect = 0.211, 95% CI (0.141–0.970),|^2 ^= 68.49%, *p*-value = 9.00E-03, Fig. 3). As the forest plots demonstrate, although there was a relatively high heterogeneity across studies, the directionality and magnitude of effect on AAO were in concordance with the meta-analysis with the exception of the Oslo cohort. This finding could suggest that firstly, disease causation and progression are genetically separable processes in PD and that secondly the role of mitochondrial dysfunction in PD is likely to be highly complex with multiple distinct mitochondrial processes likely to be involved at different disease stages.

### Mendelian randomization suggests potential causal association of 14 novel mitochondria function genes with PD risk

Given the robust evidence for the involvement of mitochondrial function in sporadic PD, next we used two-sample MR analysis to identify specific genes likely to be important in PD risk. MR uses genetic variants to identify if an observed association between a risk factor and an outcome is consistent with causal effect.^29^ This method has been implemented in several recent genetic studies to identify association between expression quantitative trait loci (QTL), to more accurately nominate candidate genes within risk loci. Therefore, for this study, in the aim of identifying whether changes in expression of mitochondria function genes are potentially causally related to PD risk, two-sample MR was implemented.

Since we wanted to identify novel associations, we excluded genes already linked to PD through the most recent GWAS meta-analysis.^6^ This resulted in the exclusion of 31 genes linked to mitochondrial function and in linkage disequilibrium with the top PD risk variants. Analysis of the remaining 1432 genes (generated by combining the primary and secondary gene lists) resulted in the identification of 14 novel genes linked to mitochondrial function and causally associated with PD risk (Table 3). Of the 14 genes, the expression of five genes (*CLN8, MPI, LGALS3, CAPRIN2*, and *MUC1)* was positively associated with PD risk in blood. Similarly, in brain PD risk was associated with increased expression of *ATG14, E2F1*, and *EP300* in brain. However, negative associations in brain and blood expression were observed for *MRPS34* and *PTPN1 and LMBRD1*, respectively. Finally, elevated methylation of *FASN* in the brain was found to be positively associated with PD risk but elevated methylation of *CRY2* was found to be inversely correlated.

**Table 3:** Significant functional associations of mitochondrial function genes via two-sample Mendelian randomization

Genes	Beta	SE	*P*, FDR, adjusted	Probe	Data source	Analyte	Top QTL SNP	CHR, top QTL SNP	BP, top QTL SNP	Associated phenotype in OMIM	Neurological phenotypic features	Treatment response
*LMBRD1*	−0.173	0.050	4.71E-04	ENSG00000117245	Expression^57^	Brain	rs7763213	6	70416294	Methylmalonic aciduria and homocystinuria, CBLF type; MAHCF	Hypotonia, lethargy, developmental delay, Impaired coordination	Responsive to vitamin B12 therapy
*ATG14*	0.113	0.028	4.21E-05	ENSG00000171612	Expression^57^	Brain	rs11621265	14	55822095	NA	NA	NA
*E2F1*	0.121	0.032	1.52E-04	ENSG00000159423	Expression^57^	Brain	rs57668191	20	32289763	NA	NA	NA
*EP300*	0.151	0.042	2.80E-04	ENSG00000090432	Expression^57^	Brain	rs139486	22	41627654	Rubinstein-taybi syndrome 2; RTS2	Behavioral difficulties, mental retardation (mild to moderate), low-normal intelligence, Autism spectrum disorder (in some patients), delayed psychomotor development, delayed gross motor development, speech delay	NA
*MRPS34*	−0.353	0.100	4.04E-04	ILMN_2210482	Expression^58^	Blood	rs2575369	16	1817431	Combined oxidative phosphorylation deficiency 32; COXPD32	Delayed psychomotor development, lack of speech, inability to walk, spasticity, hyperreflexia, dystonia, choreoathetoid movements, abnormal T2-weighted signals in the basal ganglia and brainstem	NA
*PTPN1*	−0.090	0.022	5.27E-05	ILMN_1681591	Expression^58^	Blood	rs17788127	20	49166548	NA	NA	NA
*MRPL43*	0.047	0.014	8.78E-04	ILMN_1652147	Expression^58^	Blood	rs2863095	10	102746503	NA	NA	NA
*CLN8*	0.148	0.047	1.54E-03	ILMN_1701094	Expression^58^	Blood	rs3812477	8	1734452	Ceroid lipofuscinosis, neuronal, 8; CLN8	Developmental regression, seizures, ataxia, speech and language difficulties, myoclonus, EEG abnormalities, cerebral atrophy, cerebellar atrophy, autofluorescent l ipopigment in neurons	NA
*MPI*	0.214	0.068	1.52E-03	ILMN_1761262	Expression^58^	Blood	rs4886636	15	75196176	Congenital disorder of glycosylation, type Ib; CDG1B	Hypotonia	Responsive to oral mannose therapy
*LGALS3*	0.307	0.085	3.14E-04	ILMN_1803788	Expression^58^	Blood	rs7157678	14	55548739	NA	NA	NA
*CAPRIN2*	0.337	0.101	8.49E-04	ILMN_2345739	Expression^58^	Blood	rs11051061	12	30914668	NA	NA	NA
*MUC1*	0.487	0.118	3.39E-05	ILMN_1756992	Expression^58^	Blood	rs6427184	1	155122783	Medullary cystic kidney disease 1; MCKD11	NA	NA
*CRY2*	−0.054	0.015	1.82E-04	ch.11.939596F	Methylation^57^	Brain	rs72902436	11	45881792	NA	NA	NA
*FASN*	0.068	0.019	4.47E-04	cg03407524	Methylation^57^	Brain	rs9905991	17	80052073	NA	NA	NA

Multi-SNP eQTL Mendelian randomization results focusing on the mitochondria-associated genes (combining the primary and secondary gene lists). Showing the 14 mitochondria function-associated genes that are significantly associated with PD risk after FDR adjustment.

Six of the fourteen novel PD risk genes we identified (*CLN8*, *EP300*, *LMBRD1*, *MPI*, *MRPS34*, and *MUC1*) are already associated with a monogenic disorder (Table 3). We noted that neurological abnormalities were a feature of the condition in five of the six cases with Combined Oxidative Phosphorylation Deficiency 32 due to biallelic mutations in *MRPS34* being perhaps of particular interest. In common with PD, this condition is associated with abnormalities of movement, including dystonia and choreoathetoid movements. Mutations causing this condition result in decreased levels of *MRPS34* protein causing destablization of the small mitochondrial ribosome subunit and suggesting the involvement of mitochondrial processes distinct from mitophagy and mitochondrial homeostasis in PD. In this context, it is noteworthy that *MRPL43*, another nuclear gene encoding for a component of the large mitochondrial ribosome subunit is also highlighted by the MR analysis. Thus, this analysis not only enabled us to identify specific genes of interest, but also pointed to the role of multiple mitochondrial processes in PD distinct from mitophagy.

### Exploring the expression of the novel mitochondria risk genes provides additional support for their role in PD

We leveraged publicly available cell-specific and tissue-specific gene expression data to investigate the 14 mitochondria genes implicated in PD through MR. First, we used enrichment-weighted cell-type enrichment (EWCE) to determine whether the expression of mitochondrial PD-associated genes (as identified through MR and described above, *n* = 14) was enriched within a specific cell-type class or their subtypes. No significant enrichment of these genes was found in any of the tested neuronal and glial cell-type classes (Supplementary Table 6 and Supplementary Fig. 3). Next, we used co-expression network analysis to identify possible functional interactions between the 14 novel mitochondrial genes identified through MR and genes implicated in monogenic forms of PD. We found that five of the 14 genes assessed, *CLN8*, *FASN*, *MPI*, *MRPL43*, and *MRPS3*, were highly co-expressed with at least one gene already implicated in monogenic PD in multiple brain regions ( > 3 brain regions, Supplementary Table 7). Interestingly, in the case of CLN8, *MRPL43*, and *MRPS4*, our novel genes were co-expressed with a monogenic PD gene already implicated in mitochondrial function such as *PARK7*. Furthermore, with the exception of *CLN8* (*FASN*, *MPI*, *MRPL43* and *MRPS3*), the novel mitochondrial gene was assigned to a co-expression module enriched for neuronal markers (Supplementary Table 8).

## Discussion

We first demonstrate that a proportion of the “missing heritability” of sporadic PD can be explained by additive common genetic variation within genes implicated in mitochondrial function, even after exclusion of genes previously linked to PD through linkage disequilibrium with the top risk variants.^4–6,30–34^ We identify that the overall heritability of PD is approximately 23% (Table 2) and that when heritability is calculated for the secondary gene list regions alone we can attribute around 7% of the overall heritability (23%) to common variation within the mitochondria function associated genes. Although this initially looks like a low estimate, to put this into context, when heritability was calculated in the most recent meta-analysis using the top PD risk hits, this estimate explained only 26–36% of the overall heritability.^6^ In addition, using PRS, which efficiently incorporates information from sub-significant hits, we showed that cumulative small effect variants within only 196 genes linked to monogenic mitochondrial disease significantly associated with increased PD risk (with odds ratios of 1.12 per standard deviation increase in PRS from the population mean). These findings are important for two main reasons. Firstly, given that risk profiling performed in the recent PD meta-analysis did not identify a significant association with mitochondrial function.^4–6,17,30–34^ Secondly, since the primary gene list consisted solely of the 196 genes mutated in monogenic mitochondrial disorders, this analysis highlights the increasingly close relationship between Mendelian and complex disease.^7^

In order to maximize the utility of this study, we used MR, which identified 14 specific mitochondrial genes of interest with putative functional consequences in PD risk. We found that although a number of the genes we identified are clearly linked to known PD-related pathways, such as lysosomal dysfunction in the case of *CLN8* and *LMBRD1* or autophagy in the case of *ATG14*, others appeared to point towards new processes. In particular, this analysis highlighted the mitochondrial ribosome through the identification of the genes, *MRPL43* and *MRPS34*, encoding components of the large and small mitochondrial ribosome subunits, respectively. Interestingly, biallelic mutations in *MRPS34* are known to cause a form of Leigh syndrome, characterized by neurodegeneration in infancy with dystonia and choreoathetoid movements due to basal ganglia dysfunction. Furthermore, we note that a recent study that utilized whole exome sequencing (WES) data from two PD cohorts to investigate^35,36^ rare variation in nuclear genes associated with distinct mitochondrial processes, not only provided support for the involvement of mitochondrial function in sporadic PD, but also identified the gene, *MRPL43*, which encodes a component of the large mitochondrial ribosomal subunit.^37^ Interestingly, *MRPL43* and *MRPS34* were amongst five genes, which were also highly co-expressed in human brain with genes already known to cause monogenic forms of PD. Whereas *MRPL43* and *MRPS34* were highly co-expressed with *PARK7* in modules enriched for neuronal markers, *FASN* and *MPI* were co-expressed with *ATP13A2*, and *CLN8* was located in modules containing *FBXO7* and enriched for oligodendrocyte markers. While this form of analysis does not provide information at the single-cell level, it points to the possibility of pathway interactions between these gene sets. However, most importantly it implicates entirely distinct mitochondrial processes in PD risk.

Finally, and perhaps most remarkably using our mitochondrial gene lists we observe clear differences between disease causation and AAO in PD. Although PRS of the primary mitochondrial gene list was not significantly associated with AAO, the PRS of the secondary mitochondrial gene list was positively correlated (*p*-value = 3.6E-05), indicating association with later age at onset. However, given these findings it seems plausible that some mitochondrial processes may contribute to PD risk. Thus, this analysis is consistent with the findings of the most recent and largest AAO PD GWAS, which reported that not all the well-established risk loci are associated with AAO and suggested a different mechanisms for PD causation and AAO.^38^ This has also been shown in previous studies that have shown no association of risk loci with AAO in sporadic PD.^35,39^

Although in this study we have comprehensively analyzed the largest PD datasets currently available with very specific and inclusive mitochondrial function gene lists, there are a number of limitations to our analyses. Firstly, there was a relative amount of heterogeneity in AAO within the AAO GWAS studies used. This was due to certain cohorts AAO being self-reported and other cohorts specifically recruiting younger onset cases. Nonetheless, the highly significant *p*-value we obtain for the association mitochondrial genes and AAO of PD (*p*-value = 3.56E-05) and the recognized importance of mitochondrial function in aging would suggest that this finding is likely to be robust. Furthermore, it is important to recognize that our understanding of mitochondrial biology is far from complete and this is made evident by the fact that many individuals with probable genetic forms of mitochondrial disease remain undiagnosed. This in conjunction with the fact we also removed the mitochondria function-associated genes that are known risk hits (such as SNCA), suggests that our analysis likely represents an underestimate of the overall contribution of the mitochondria pathway to sporadic PD. This underestimate is illustrated in Supplementary Figs. 1 and 2, which shows that heritability estimates and PRS scores are higher when the PD risk genes are included. However, we note that our ultimate goal for this study was to catalog mitochondria-specific genetic risk outside of known risk loci, which we have subsequently reported.

Another possible limitation of this present study is that the statistical tools we have used in these analyses are currently limited. For example, MR ultimately relies on the availability of sufficient quantities of high-quality eQTL data. However, as there is a future focus to; increase dataset sample size, report and characterize phenotypes such as AAO more accurately and continue to increase the number of identified mitochondrial disease and function genes, we will be able to further explore the role of specific mitochondrial processes in more detail and identify their distinct contribution to disease causation and progression. In regard to the MR nominated genes in particular, further follow-up functional studies will be crucial to validate how these genes contribute to disease risk. Finally, it is possible that our focus on one specific pathway could infer a selection bias to our analyses. However, the fact that our results are consistent across cohorts and the fact that this significant association is observed in multiple tests, adds validity to our data. In light of this, however, a large-scale unbiased approach should be the focus of future pathway related studies to avoid this potential bias, although this will be difficult given the scope of work and sample size needed.

In summary, in this study we provide robust evidence for the involvement of mitochondrial processes in sporadic PD, as opposed to its defined and well-established role in the monogenic forms of the disease. In relation to the 14 novel mitochondrial function genes that we have identified, our data suggests that it is not only mitochondrial quality control and homeostasis, which contributes to PD risk but other key mitochondrial processes, such as the function of mitochondrial ribosomes, mirroring the biological complexity of mitochondrial disorders. Thus, this study opens the way for the identification of novel drug targets in PD causation and progression.

## Methods

### Samples and quality control of IPDGC datasets

All genotyping data was obtained from previously generated IPDGC datasets, consisting of 41,321 individuals (18,869 cases and 22,452 controls) of European ancestry. For the IPDGC datasets all participants donated DNA samples and provided informed consent for participation in genetics studies, which was approved by the local ethic committee for each of the datasets used (National Institutes of Health, Department of Health and Human Services; project ZO1 AG000949). Detailed demographic and clinical characteristics are given in Supplementary Table 1 and are explained in further detail in refs. ^6,38^ along with detailed quality control (QC) methods. For sample QC, in short, individuals with low call rate ( < 95%), discordance between genetic and reported sex, heterozygosity outliers (*F*-statistic cutoff of > −0.15 and < 0.15) and ancestry outliers ( + /− 6 standard deviations from means of eigenvectors 1 and 2 of the 1000 Genomes phase 3 CEU and TSI populations from principal components^40^) were excluded. Further, for genotype QC, variants with a missingness rate of > 5%, minor allele frequency < 0.01, exhibiting Hardy–Weinberg Equilibrium (HWE) < 1E-5 and palindromic SNPs were excluded. Remaining samples were imputed using the Haplotype Reference Consortium (HRC) on the University of Michigan imputation server under default settings with Eagle v2.3 phasing based on reference panel HRC r1.1 2016.^41,42^

### Curation of genes implicated in mitochondrial disorders and associated with mitochondrial function

Gene lists were built to encompass different levels of evidence for involvement of the respective protein products in disease phenotypes that relate to mitochondrial function. The list of genes implicated in genetic mitochondrial disorders (“primary” gene list, *n* = 196) has the most stringent criteria of evidence that the respective genes is related to mitochondrial dysfunction. It consists of 102 nuclear genes listed in MITOMAP (downloaded 2015) and 94 sourced from literature review as containing mutations that cause with mitochondrial disease.

The list of genes implicated in mitochondrial function (“secondary” gene list, *n* = 1487) was constructed using the OMIM API to identify all genes for which the word “mitochondria” (or derivatives) appeared in the free-text description, and by combining this information with MitoCarta v2.0 genes with no OMIM phenotype. This therefore gathered a list of plausible biological candidate genes, i.e., genes that are functionally implicated in mitochondrial function and morphology for which we may lack genetic evidence for mitochondrial disease association.

Next, to identify novel PD-associated genes, the 349 genes identified to be in LD with the PD risk variants of interest in the most recent PD meta-analysis^6^ were removed from both lists (removed genes listed in Supplementary Table 2). The final “primary” and “secondary” gene lists are given in Supplementary Table 3 and Supplementary Table 4 and following the removal the PD-associated genes were *n* = 178 and *n* = 1328, respectively.

### Cohort-level heritability estimates and meta-analysis

Genome-wide complex trait analysis (GCTA) was used to calculate heritability estimates for the four largest IPDGC cohorts (WTCCC PD GWAS (PMID:21044948), Spanish Parkinson’s (IPDGC) part2, NIA PD GWAS (PMID:19915575), Dutch GWAS (PMID:21248740) using non-imputed genotyping data for all variants within both mitochondria gene lists using the same workflow as in ref. ^43^ Genetic relationship matrices were calculated for each dataset to identify the genetic relationship between pairs of individuals. Genetic relationship matrices were then input into restricted maximum likelihood analyses to produce estimates of the proportion of phenotypic variance explained by the SNPs within each subset of data. Principal components (PCs) were generated for each dataset using PLINK (version 1.9). In order to adjust for factors accounting to possible population substructure, the top 20 generated eigenvectors from the PC analysis, age, sex, and prevalence were used as basic covariates. Disease prevalence standardized for age and gender based on epidemiological reports was specified at 0.002.^43–47^ Summary statistics from these estimates were produced for all four datasets and were included in the meta-analyses. Random-effects meta-analysis using the residual maximum likelihood method was performed using R (version 3.5.1) package metafor to calculate *p*-values and generate forest plots.^48^

### Risk profiles versus disease status and age at onset

Previous risk profiling methods have calculated polygenic risk scores (PRS) using only variants that exhibit genome-wide significant associated with disease risk. However, in the most recent PD meta-analysis, it is shown that using variants at thresholds below genome-wide significance improves genetic predictions of disease risk.^6,43^ Mirroring this workflow, but instead using only variants within gene regions outlined in both the primary and secondary gene lists, the R package PRsice2 was used to carry out PRS profiling in the standard weighted allele dose manner. In addition, PRsice2 performs permutation testing and *p*-value aware LD pruning to facilitate identifying the best *p*-value threshold for variant inclusion to construct the PRS. External summary statistics utilized in this phase of analysis included data from leave-one-out meta-analyses (LOOMAs) that exclude the study in which the PRS was being tested, avoiding overfitting/circularity to some degree. LD clumping was implemented under default settings (window size = 250 kb, *r*^2^ > 0.1) and for each dataset 10,000 permutations of phenotype-swapping were used to generate empirical *p*-value estimates for each GWAS derived *p*-value threshold ranging from 5E-08 to 0.5, at a minimum increment of 5E-08. Each permutation test in each dataset provided a Nagelkerke’s pseudo *r*^2^ after adjustment for an estimated prevalence of 0.005 and study-specific PCs 1–5, age and sex as covariates. GWAS derived *p*-value threshold with the highest pseudo *r*^2^ was selected for further analysis. Summary statistics were meta-analyzed using random effects (REML) per study-specific dataset using PRSice2.^49^ For the age at onset risk profiling, the same workflow was followed, however instead, age at onset was used as a continuous variable, as previously reported.^38^ To remove possible confounders that could possibly drive a false association with age we removed *APOE* and *FOXO3*, which are general markers for aging.^50^

### Mendelian randomization to explore possible causal effect of mitochondria function genes

Both mitochondria gene lists were combined, and all genes already associated with PD (i.e., that have been identified to be in LD with PD risk loci in the last meta-analysis) were removed, leaving 1432 unique mitochondria function gene regions.

We utilized four large-scale methylation and expression datasets through the summary-data-based Mendelian randomization (SMR) (http://cnsgenomics.com/software/smr) resource. Summary statistics were compared to PD outcome summary statistics for the mitochondria variants of interest (extracted from refs. ^4–6,30–34^) to identify possible association using the R package TwoSampleMR.

Tissues were selected based on their relevance to the pathobiology of PD, which ultimately consisted of tissues from ten brain regions, whole blood, skeletal muscle, and nerve (a full list of the tissues utilized can be found in Supplementary Table 5). For the methylation QTLs “middle age” data was used, which was the oldest available time point. For each mitochondria function variant of interest (considered here the instrumental variable), wald ratios were generated for each variable that tagged a cis-QTL (probes within each gene and meeting a QTL *p*-value of at least 5E-08 in the original QTL study) and for a methylation or expression probe with a nearby gene. Using the default *SMR* protocols, linkage pruning and clumping were implemented. Finally, for each dataset *p*-values were adjusted by false discovery rate to account for multiple testing.

### Co-expression network analysis

Co-expression network analysis was used to determine whether mitochondrial genes associated with PD using the SMR analysis are co-expressed with genes associated with monogenic forms of PD in human brain. This analysis was performed by using GTEx V6 gene expression data^51^ to generate co-expression networks for each of the 13 brain tissues included within the GTEx study. The raw FPKM (Fragments Per Kilobase of transcript per Million mapped reads) values were corrected for known batch effects, age at death, sex and post-mortem interval, as well as unknown effects. The unknown effects were detected with the Surrogate Variable Analysis (SVA) R Package^52^ and correction was performed using ComBat^53^. The resulting residuals were used to create a signed network using the blockwiseConsensusModules R function from the WGCNA R package^54^ for each of the 13 tissues. Next, the modules obtained in each of the 13 networks were assigned to cell types using the user List Enrichment R function implemented in the WGCNA R package, which measures enrichment between module-assigned genes and defined brain-related lists using a hypergeometric test. The same approach was used to annotate modules with Gene Ontology, REACTOME^55^ and KEGG^56^ terms.

### Expression-weighted cell-type enrichment (EWCE): evaluating enrichment of mitochondrial genes associated with PD risk

Expression-weighted cell-type enrichment (EWCE) (https://github.com/NathanSkene/EWCE) (EWCE) was used to determine whether mitochondrial genes associated with PD using the MR analysis have higher expression within a particular cell type than expected by chance. The input for the analysis was (1) neuronal and glial clusters of the central nervous system (CNS) identified in the Linnarsson single-cell RNA sequencing dataset (amounting to a subset of 114 of the original 265 clusters identified) (http://mousebrain.org/) and (2) our list of mitochondrial genes highlighted through the MR analysis (see Supplementary Table 6 for the full list of Linnarsson CNS neuronal clusters used).

For each gene in the Linnarsson dataset, cell-type specificity was estimated (the proportion of a gene’s total expression in one cell-type compared to all cell types) using the ‘generate.celltype.data’’ function of the EWCE package. EWCE with the target list was run with 100,000 bootstrap replicates, which were sampled from a background list of genes that excluded all genes without a 1:1 mouse:human ortholog. In addition, transcript length and GC-content biases were controlled for by selecting bootstrap lists with comparable properties to the target list. The analysis was performed using major cell-type classes (e.g., “telencephalon inhibitory interneurons”, “telencephalon projecting excitatory neurons”, etc.) and subtypes of these classes (e.g., TEINH6 [“Interneuron-selective interneurons, cortex/hippocampus”], TEINH7 [“Interneuron-selective interneurons, hippocampus”] etc.). Data are displayed as standard deviations from the mean, and any values < 0, which reflect a depletion of expression, are displayed as 0. *p*-values were corrected for multiple testing using the Benjamini–Hochberg method over all cell types and gene lists displayed.

### OMIM data

Phenotype relationships and clinical synopses of all OMIM genes were downloaded from http://api.omim.org on the 29th of May 2018. OMIM genes were filtered to exclude provisional, non-disease and susceptibility phenotypes retaining 2898 unique genes that were confidently associated to 4034 Mendelian diseases. The phenotypic information relating to all genes associated with mitochondrial disorders was collated.

## Supplementary information

Supplementary Figures and Tables

## Data Availability

For all datasets included in this study GWAS summary statistics are available at: https://drive.google.com/file/d/1FZ9UL99LAqyWnyNBxxlx6qOUlfAnublN/view?usp=sharing Underlying participant level IPDGC data is available to potential collaborators, please contact ipdgc.contact@gmail.com.

## Supplementary information

**Supplementary Information** accompanies the paper on the *npj Parkinson’s Disease* website (10.1038/s41531-019-0080-x).

## References

[1] Singleton A, Hardy J (2016). The evolution of genetics: Alzheimer’s and Parkinson’s Diseases. Neuron.

[2] Gasser T (1998). Genetics of parkinson’s disease. Ann. Neurol..

[3] Reeve A, Simcox E, Turnbull D (2014). Ageing and Parkinson’s disease: why is advancing age the biggest risk factor?. Ageing Res. Rev..

[4] Chang D (2017). A meta-analysis of genome-wide association studies identifies 17 new Parkinson’s disease risk loci. Nat. Genet..

[5] Nalls MA (2014). Large-scale meta-analysis of genome-wide association data identifies six new risk loci for Parkinson’s disease. Nat. Genet..

[6] Nalls, M. A. et al. Parkinson’s disease genetics: identifying novel risk loci, providing causal insights and improving estimates of heritable risk (2018). 10.1101/388165

[7] Robak LA (2017). Excessive burden of lysosomal storage disorder gene variants in Parkinson’s disease. Brain.

[8] Hardy J (2010). Genetic analysis of pathways to Parkinson disease. Neuron.

[9] Billingsley KJ, Bandres-Ciga S, Saez-Atienzar S, Singleton AB (2018). Genetic risk factors in Parkinson’s disease. Cell Tissue Res..

[10] Langston JW, Ballard P, Tetrud JW, Irwin I (1983). Chronic Parkinsonism in humans due to a product of meperidine-analog synthesis. Science.

[11] Langston JW, Ballard PA (1983). Parkinson's disease in a chemist working with 1-methyl-4-phenyl-l,2,5,6-tetrahydropyridine. N. Engl. J. Med..

[12] Parker WD, Parks JK, Swerdlow RH (2008). Complex I deficiency in Parkinson's disease frontal cortex. Brain Res..

[13] Canet-Avilés RM (2004). The Parkinson’s disease protein DJ-1 is neuroprotective due to cysteine-sulfinic acid-driven mitochondrial localization. Proc. Natl Acad. Sci. USA.

[14] Funayama M (2015). CHCHD2 mutations in autosomal dominant late-onset Parkinson’s disease: a genome-wide linkage and sequencing study. Lancet Neurol..

[15] Burchell VS (2013). The Parkinson’s disease-linked proteins Fbxo7 and Parkin interact to mediate mitophagy. Nat. Neurosci..

[16] Lesage S (2016). Loss of VPS13C function in autosomal-recessive Parkinsonism causes mitochondrial dysfunction and increases PINK1/Parkin-dependent mitophagy. Am. J. Hum. Genet..

[17] Gorman GS (2016). Mitochondrial diseases. Nat. Rev. Dis. Prim..

[18] Goebel HH, Heipertz R, Scholz W, Iqbal K, Tellez-Nagel I (1978). Juvenile Huntington chorea: Clinical, ultrastructural, and biochemical studies. Neurology.

[19] Carmo, C., Naia, L., Lopes, C. & Rego, A. C. Mitochondrial Dysfunction in Huntington’s Disease. In *Advances in Experimental Medicine and Biology* 59–83 (2018). 10.1007/978-3-319-71779-1_3 29427098

[20] Atsumi T (1981). The ultrastructure of intramuscular nerves in amyotrophic lateral sclerosis. Acta Neuropathol..

[21] Sasaki S, Iwata M (2007). Mitochondrial alterations in the spinal cord of patients with sporadic amyotrophic lateral sclerosis. J. Neuropathol. Exp. Neurol..

[22] Moreira PI, Cardoso SM, Santos MS, Oliveira CR (2006). The key role of mitochondria in Alzheimer’s disease. J. Alzheimers Dis..

[23] Nunomura A (2001). Oxidative damage is the earliest event in Alzheimer disease. J. Neuropathol. Exp. Neurol..

[24] Moreira PI, Duarte AI, Santos MS, Cristina Rego A, Oliveira CR (2009). An integrative view of the role of oxidative stress, mitochondria and insulin in Alzheimer’s disease. J. Alzheimers Dis..

[25] Fang EF (2019). Mitophagy inhibits amyloid-β and tau pathology and reverses cognitive deficits in models of Alzheimer's disease. Nat. Neurosci..

[26] Nalls MA (2015). Genetic risk and age in Parkinson’s disease: Continuum not stratum. Mov. Disord..

[27] Escott-Price V (2015). Polygenic risk of Parkinson disease is correlated with disease age at onset. Ann. Neurol..

[28] Lill CM (2015). Impact of Parkinson’s disease risk loci on age at onset. Mov. Disord..

[29] Lawlor DA, Harbord RM, Sterne JAC, Timpson N, Davey Smith G (2008). Mendelian randomization: using genes as instruments for making causal inferences in epidemiology. Stat. Med..

[30] International Parkinson Disease Genomics Consortium (2011). Imputation of sequence variants for identification of genetic risks for Parkinson’s disease: a meta-analysis of genome-wide association studies. Lancet.

[31] Satake W (2009). Genome-wide association study identifies common variants at four loci as genetic risk factors for Parkinson’s disease. Nat. Genet..

[32] Simón-Sánchez J (2009). Genome-wide association study reveals genetic risk underlying Parkinson’s disease. Nat. Genet..

[33] Pihlstrøm L (2013). Supportive evidence for 11 loci from genome-wide association studies in Parkinson’s disease. Neurobiol. Aging.

[34] Lill CM (2012). Comprehensive research synopsis and systematic meta-analyses in Parkinson’s disease genetics: The PDGene database. PLoS Genet..

[35] Hill-Burns EM (2016). Identification of genetic modifiers of age-at-onset for familial Parkinson’s disease. Hum. Mol. Genet..

[36] Brockmann, K. et. al. SNCA: major genetic modifier of age at onset of Parkinson’s disease. -PubMed- NCBI. Available at: https://www.ncbi.nlm.nih.gov/pubmed/23674386. (Accessed 1 Mar 2019) 10.1002/mds.25469 23674386

[37] Gaare JJ (2018). Rare genetic variation in mitochondrial pathways influences the risk for Parkinson’s disease. Mov. Disord..

[38] Blauwendraat, C. et al. Parkinson disease age of onset GWAS: defining heritability, genetic loci and a-synuclein mechanisms. (2018). 10.1101/424010 10.1002/mds.27659 PMC6579628 30957308

[39] Huang Y (2015). SNCAGene, but NotMAPT, influences onset age of Parkinson’s disease in Chinese and Australians. BioMed. Res. Int..

[40] 1000 Genomes Project Consortium. (2015). A global reference for human genetic variation. Nature.

[41] McCarthy S (2016). A reference panel of 64,976 haplotypes for genotype imputation. Nat. Genet..

[42] Das S (2016). Next-generation genotype imputation service and methods. Nat. Genet..

[43] Keller MF (2012). Using genome-wide complex trait analysis to quantify ‘missing heritability’ in Parkinson’s disease. Hum. Mol. Genet..

[44] Wirdefeldt K, Gatz M, Reynolds CA, Prescott CA, Pedersen NL (2011). Heritability of Parkinson disease in Swedish twins: a longitudinal study. Neurobiol. Aging.

[45] Gasser T (2005). Genetics of Parkinsonʼs disease. Curr. Opin. Neurol..

[46] Wickremaratchi MM (2009). Prevalence and age of onset of Parkinson’s disease in Cardiff: a community based cross sectional study and meta-analysis. J. Neurol. Neurosurg. Psychiatry.

[47] Porter B, Macfarlane R, Unwin N, Walker R (2006). The prevalence of Parkinson’s disease in an area of North Tyneside in the north-east of England. Neuroepidemiology.

[48] Viechtbauer W (2010). Conducting meta-analyses in R with the meta for Package. J. Stat. Softw.

[49] Euesden J, Lewis CM, O’Reilly PF (2014). PRSice: polygenic risk score software. Bioinformatics.

[50] Broer L (2015). GWAS of longevity in CHARGE consortium confirms APOE and FOXO3 candidacy. J. Gerontol. A Biol. Sci. Med. Sci..

[51] Carithers LJ (2015). A Novel Approach to High-Quality Postmortem Tissue Procurement: The GTEx Project. Biopreserv. Biobank.

[52] Leek JT, Storey JD (2007). Capturing heterogeneity in gene expression studies by surrogate variable analysis. PLoS Genet..

[53] Johnson WE, Li C, Rabinovic A (2007). Adjusting batch effects in microarray expression data using empirical Bayes methods. Biostatistics.

[54] Langfelder P, Horvath S (2008). WGCNA: an R package for weighted correlation network analysis. BMC Bioinform.

[55] Fabregat A (2018). The Reactome Pathway Knowledgebase. Nucleic Acids Res.

[56] Kanehisa M, Sato Y, Kawashima M, Furumichi M, Tanabe M (2016). KEGG as a reference resource for gene and protein annotation. Nucleic Acids Res.

[57] Qi T (2018). Identifying gene targets for brain-related traits using transcriptomic and methylomic data from blood. Nat. Commun..

[58] Westra H-J (2013). Systematic identification of trans eQTLs as putative drivers of known disease associations. Nat. Genet..

