# Supplementary material for: Mitochondria function associated genes contribute to Parkinson’s Disease risk and later age at onset
Source: NPJ Parkinsons Dis. 2019 May 22;5:8. doi: 10.1038/s41531-019-0080-x (PMC6531455; doi:10.1038/s41531-019-0080-x)
Supplement: Supplementary file 1 — Supplementary Figures and Tables [file 41531_2019_80_MOESM1_ESM.pdf]

Supplementary Figures:

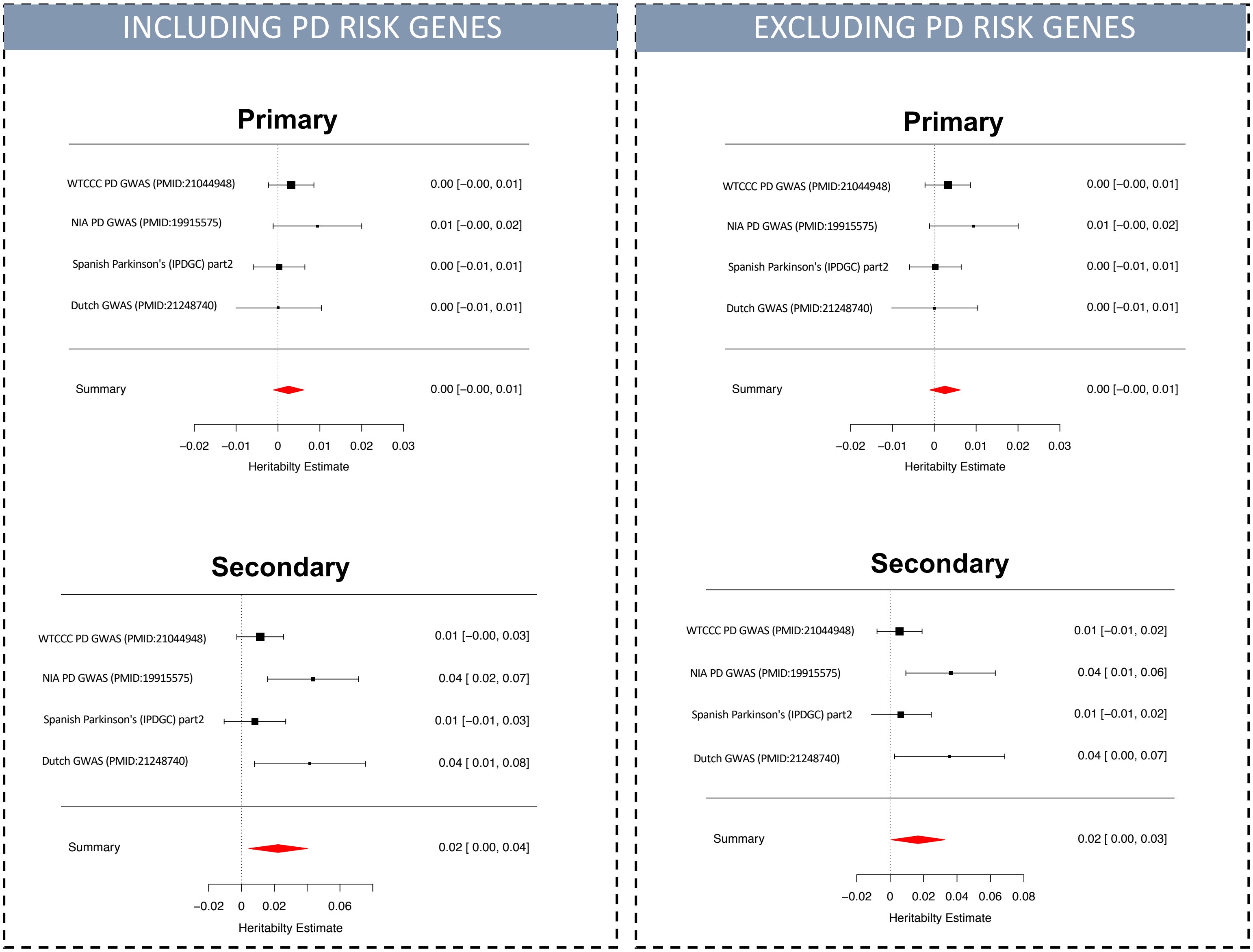

Supplementary Figure 1

Forest plots of heritability estimates across cohorts for comparison of analysis when including or excluding PD risk genes for the primary and secondary gene lists. Cohort-specific heritability estimates are shown, the size of the square is proportional to the size of the study. Confidence intervals of the summary heritability estimates are shown as red diamonds, with the centerline of each diamond representing the summary heritability estimate.

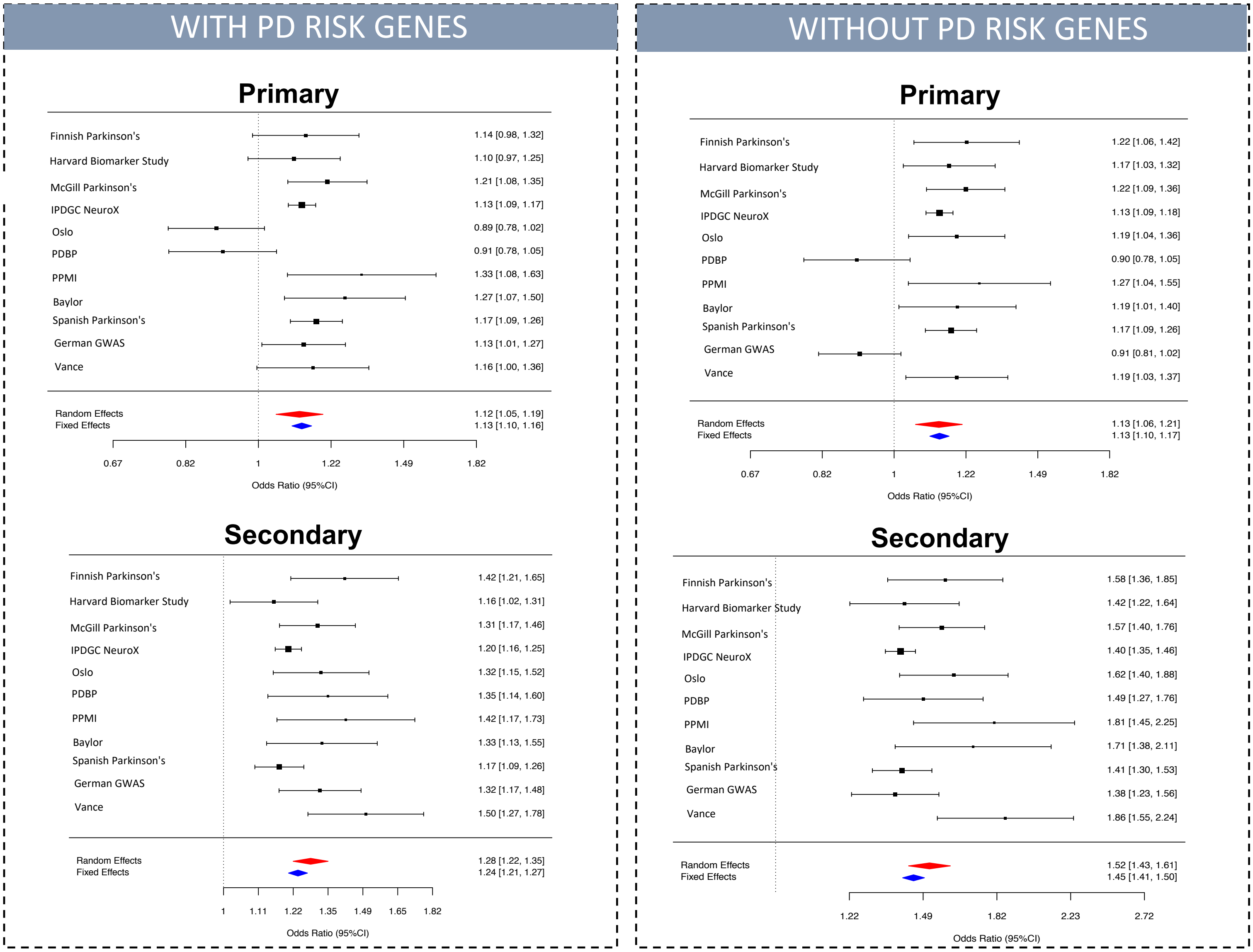

Supplementary Figure 2. Forest plots of PRS across cohorts for comparison of analysis when including or excluding PD risk genes for the primary and secondary gene lists. Random effect meta-analysis results are shown as red diamonds and fixed effects are shown as blue, with the centerline of each diamond representing the summary PRS for that dataset. IPDGC NeuroX= (Nallset al 2015, PMID:25444595), OSLO= Oslo Parkinson's Disease Study, PDBP= Parkinson's Disease Biomarker's Program, PPMI= Parkinson's Progression Markers Initiative, Baylor = Baylor College of Medicine / University of Maryland, German GWAS= (PMID:19915575), VANCE=Vance (dbGapphs000394).

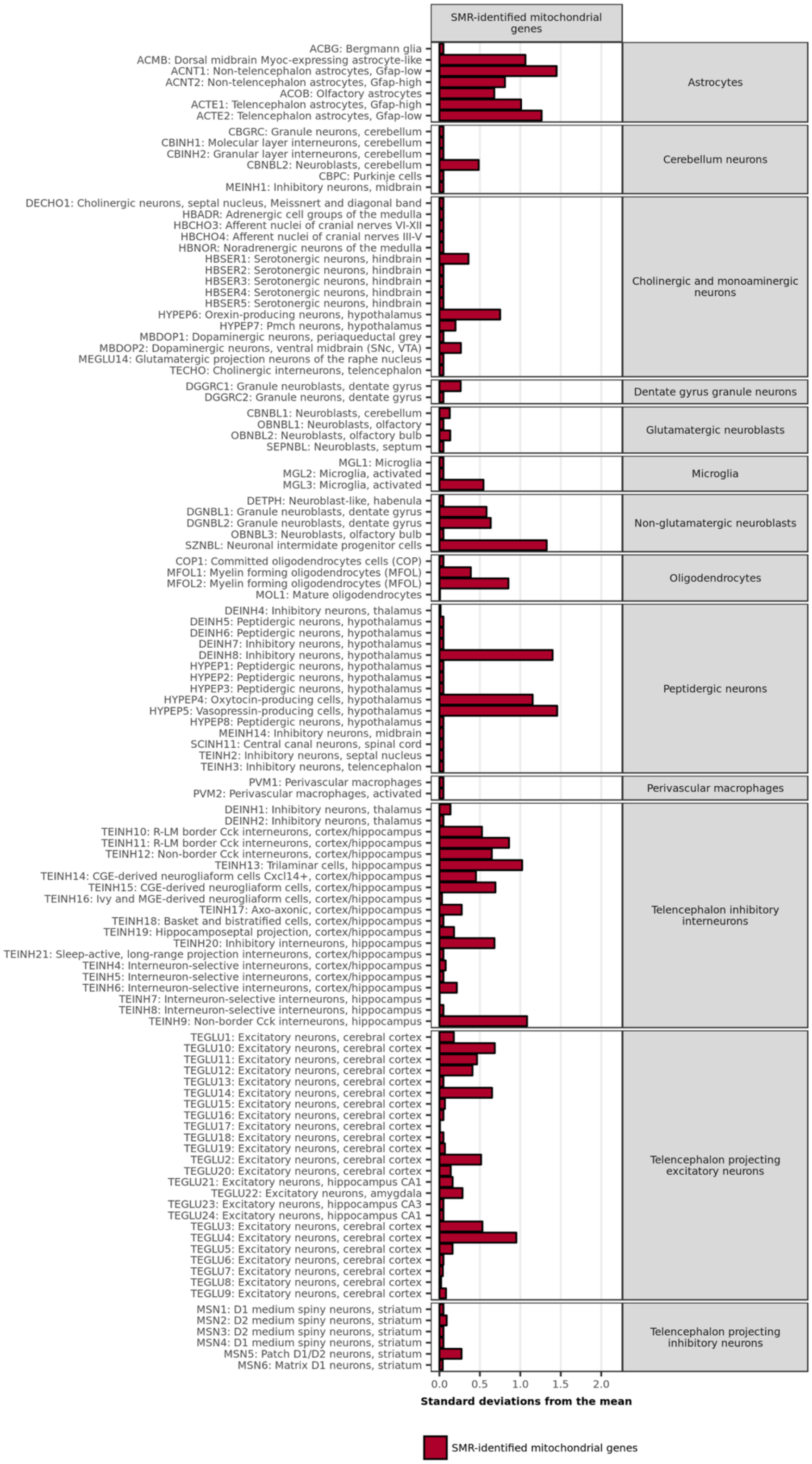

Supplementary Figure 3. Results of enrichment-weighted cell-type enrichment analysis for all genes identified by via two-sample Mendelian randomization.

Supplementary Tables:

Supplementary Table 1. Demographic and clinical characteristics for all IPDGC genotyping data

Study	Cases (n)	Controls (n)	Total (n)	Case age at onset (mean, SD in years)	Control age at last exam (mean, SD in years)
IPDGC NeuroX (Nalls et al 2015, PMID:25444595)	5533	5853	11386	61.22 (12.64)	64.34 (14.82)
WTCCC PD GWAS (PMID:21044948)	1609	5195	6804	64.07 (12.04)	NaN (NA)
NIA PD GWAS (Simón-Sánchez et al 2009, PMID:19915575)	883	3009	3892	58.21 (12.89)	63.29 (10.04)
Spanish Parkinson's (IPDGC) part1	1920	1164	3084	60.07 (12.70)	69.02 (9.95)
Dutch GWAS (PMID:21248740)	768	1987	2755	54.83 (11.1)	53.53(5.98)
PROBAND	1815	NA	1815	66.24 (9.20)	NaN (NA)
Myers-Faroud (PMID:22451204)	873	850	1723	NaN (NA)	NaN (NA)
German GWAS (PMID:19915575)	741	944	1685	55.76 (11.55)	47.42 (12.38)
McGill Parkinson's	583	906	1489	65.71 (9.78)	55.79 (10.69)
Harvard Biomarker Study (HBS)	541	473	1014	66.25 (9.97)	69.93 (9.04)
Baylor College of Medicine / University of Maryland	789	195	984	64.9 (10.11)	65.45 (8.31)
Oslo Parkinson's Disease Study	476	462	938	65.32 (9.28)	61.85 (11.06)
Vance (dbGap phs000394)	621	303	924	77.44 (8.41)	81.88 (12.73)
Finnish Parkinson's	386	493	879	55.27 (5.64)	92.35 (3.86)
Parkinson's Disease Biomarker's Program (PDBP)	543	284	827	64.59 (9.34)	62.23 (10.70)
Parkinson's Progression Markers Initiative (PPMI)	363	165	528	64.24 (9.65)	63.79 (10.59)
Spanish Parkinson's (IPDGC) part2	200	169	369	67.25 (10.55)	58.09 (13.98)
PROPARK	235	NA	235	55.69 (9.96)	NaN (NA)
TOTAL	18879	22452	41331		

Supplementary Table 2. Mitochondria function associated genes identified as in LD with top PD risk variants in the Meta.5

chr	start	stop	gene
1	156104903	156107058	LMNA
1	161475204	161489360	FCGR2A
3	48894355	48936426	SLC25A20
3	49059073	49060926	NDUFAF3
3	49133364	49142562	QARS
3	122078435	122102074	CCDC58
3	182733005	182817365	MCCC1
4	90645249	90759447	SNCA
4	170541671	170644338	CLCN3
5	60240955	60448864	NDUFAF2
5	134240809	134298336	PCBD2
6	31777395	31782835	HSPA1L
6	31783290	31785719	HSPA1A
6	32605182	32611429	HLA-DQA1
7	23338939	23349180	MALSU1
7	23349827	23509995	IGF2BP3
7	65425672	65447301	GUSB
8	11700033	11725646	CTSB
12	40618812	40763086	LRRK2
12	41302158	41466213	CNTN1
14	75127954	75179807	AREL1
14	75348593	75370450	DLST
16	28853731	28857729	TUFM
16	28889808	28915830	ATP2A1
16	28986095	28996838	SPNS1
16	50731049	50766987	NOD2
17	40687950	40696466	NAGLU
17	40714091	40718299	COASY
17	42325757	42345502	SLC4A1
17	76374734	76420639	PGS1
20	3171011	3185295	DDRGK1

Supplementary Table 3. Primary mitochondria gene list hg/19

chr	start	stop	Gene
1	17345224	17380665	SDHB
1	39491966	39500308	NDUFS5
1	53662100	53679869	CPT2
1	100315639	100389579	AGL
1	161171936	161184184	NDUFS2
1	161284165	161334535	SDHC
1	173793796	173827682	DARS2
1	179262848	179327814	SOAT1
1	227127937	227175246	ADCK3
1	235272657	235292256	TOMM20
1	241660856	241683085	FH
1	244998638	245008359	COX20
1	12040237	12073572	MFN2
1	45965855	45976739	MMACHC
1	76190042	76229355	ACADM
1	64088886	64125916	PGM1
2	27532359	27545969	MPV17
2	37458773	37476303	NDUFAF7
2	44113362	44223144	LRPPRC
2	55861197	55921045	PNPT1
2	69623244	69664760	NFU1
2	74153952	74186088	DGUOK
2	74362527	74375039	BOLA3
2	84650646	84686586	SUCLG1
2	86441119	86564777	REEP1
2	99215785	99224981	COA5
2	198351307	198364640	HSPD1
2	201936461	201950473	NDUFB3
2	207630111	207660911	FASTKD2
2	219524378	219528166	BCS1L
2	224822120	224832431	MRPL44
2	228549925	228582745	SLC19A3
2	240896788	240964819	NDUFA10
2	11817704	11967533	LPIN1
2	206987802	207023918	NDUFS1
3	15643254	15687325	BTD

3	45430074	45590328	LARS2
3	58413356	58419579	PDHB
3	128598332	128631957	ACAD9
3	135969166	136049013	PCCB
3	139062797	139075887	MRPS22
3	158362316	158410360	GFM1
3	193310932	193415600	OPA1
3	81538849	81810950	GBE1
3	149235021	149375812	WWTR1
4	6271576	6304992	WFS1
4	84184976	84206067	COQ2
4	103790134	103813963	CISD2
4	146540539	146581187	MMAA
4	159593276	159629841	ETFDH
4	186064416	186071538	SLC25A4
5	218355	256814	SDHA
5	52856464	52979171	NDUFS4
5	70883114	70954530	MCCC2
5	74017030	74063042	GFM2
5	131705400	131731306	SLC22A5
5	132202318	132204536	UQCRQ
5	140024947	140027370	NDUFA2
5	140071017	140078890	HARS2
5	145492588	145562294	LARS
6	5261583	5771816	FARS2
6	49398072	49431041	MUT
6	70385640	70507049	LMBRD1
6	74171453	74211179	MTO1
6	97337186	97345767	NDUFAF4
6	99321600	99395849	FBXL4
6	99817347	99842082	COQ3
6	131894343	131905472	ARG1
6	151725896	151773316	RMND1
6	155577263	155635617	TFB1M
6	158530535	158589312	SERAC1
6	44266462	44281063	AARS2
6	166778407	166796501	MPC1
7	44102325	44105186	PGAM2
7	95749531	95951459	SLC25A13
7	107531585	107561643	DLD

7	144149033	144533146	TPK1
8	15397595	15624158	TUSC3
8	94929082	94938296	PDP1
8	96037213	96070944	NDUFAF6
8	125551342	125562227	NDUFB9
8	97238903	97247862	UQCRB
8	103216728	103251059	RRM2B
9	32553523	32573182	NDUFB6
9	32972603	33001639	APTX
9	71650478	71693993	FXN
9	93976096	94124206	AUH
9	97365420	97401923	FBP1
9	136218665	136223361	SURF1
9	138392476	138396519	MRPS2
10	26986594	27035726	PDSS1
10	30598729	30638267	MTPAP
10	75008600	75012451	MRPS16
10	101468504	101492423	COX15
10	102747292	102754158	C10orf2
10	70173820	70231730	DNA2
11	34937676	35017675	PDHX
11	61197596	61214239	SDHAF2
11	64513860	64528187	PYGM
11	66615996	66675340	PC
11	67374322	67380012	NDUFV1
11	67798083	67804114	NDUFS8
11	68522350	68609399	CPT1A
11	78147006	78285909	NARS2
11	107992257	108018891	ACAT1
11	111895537	111935002	DLAT
11	111957547	111966525	SDHD
11	126138934	126148027	FOXRED1
11	118895060	118901616	SLC37A4
12	4758263	4796720	NDUFA9
12	21689122	21757781	GYS2
12	32832136	32898584	DNM1L
12	32899477	32908887	YARS2
12	48499655	48540187	PFKM
12	58176527	58191370	TSFM
12	95365103	95397489	NDUFA12

12	98987402	98995778	SLC25A3
12	109577201	109706030	ACACB
12	123717843	123742506	C12orf65
12	132413812	132428406	PUS1
12	108956293	108963160	ISCU
12	50505763	50514240	COX14
13	41363546	41386596	SLC25A15
13	48516790	48575462	SUCLA2
14	32030590	32330429	NUBPL
14	51371934	51411248	PYGL
14	74416954	74429813	COQ6
14	96001322	96011055	GLRX5
15	41679546	41694658	NDUFAF1
15	65293849	65321977	MTFMT
15	76508628	76603810	ETFA
15	89859535	89878026	POLG
16	2034149	2037750	GFER
16	8891669	8943194	PMM2
16	21529229	21531765	SLC7A5P2
16	23533333	23568696	EARS2
16	57481336	57495187	COQ9
16	66541905	66584315	TK2
16	75661621	75681585	KARS
16	85833172	85840607	COX4I1
16	89574804	89624174	SPG7
16	30759619	30772497	PHKG2
17	7123149	7128586	ACADVL
17	10583648	10600885	SCO1
17	15902693	15932723	TTC19
17	17921333	17942480	ATPAF2
17	35441926	35766902	ACACA
17	41052813	41066450	G6PC
17	42082031	42086436	NAGS
17	61678230	61685725	TACO1
17	62473901	62493184	POLG2
17	73269060	73285530	SLC25A19
18	9102627	9134343	NDUFV2
18	12328942	12377275	AFG3L2
19	1383706	1395588	NDUFS7
19	5891286	5904024	NDUFA11

19	7991602	8008708	TIMM44
19	8376183	8386280	NDUFA7
19	36486089	36487220	SDHAF1
19	36641823	36643771	COX7A1
19	44010870	44031396	ETHE1
19	51848408	51858096	ETFB
19	39405903	39421536	SARS2
20	13765671	13799067	NDUFAF5
20	17949761	17971762	MGME1
20	18568555	18744560	DTD1
20	30225690	30232800	COX4I2
20	57603732	57607422	ATP5E
21	27107328	27144771	GABPA
21	44313377	44329773	NDUFV3
21	38123188	38353264	HLCS
22	19420035	19423596	MRPL40
22	31003069	31023047	TCN2
22	41865128	41924993	ACO2
22	46731297	46753237	TRMU
22	50961996	50964033	SCO2
22	50964180	50968514	TYMP
22	51007289	51016894	CPT1B

Supplementary Table 4. Secondary mitochondria gene list hg/19

chr	start	stop	Gene
1	1243993	1247057	PUSL1
1	1407163	1431582	ATAD3B
1	2985741	3355185	PRDM16
1	6324331	6453826	ACOT7
1	6694227	6761966	DNAJC11
1	9599527	9642831	SLC25A33
1	10270763	10441661	KIF1B
1	11166587	11322608	MTOR
1	11333254	11348491	UBIAD1
1	11845786	11866160	MTHFR
1	15817895	15850940	CASP9
1	16340522	16345285	HSPB7
1	17312452	17338423	ATP13A2
1	20825940	20834674	MUL1
1	20990506	21044317	KIF17
1	24128366	24151949	HMGCL
1	24683488	24740262	STPG1
1	25071759	25170815	CLIC4
1	26146396	26159433	MTFR1L
1	26377795	26394125	TRIM63
1	27237974	27240567	NR0B2
1	27992571	27998724	IFI6
1	28562601	28564616	ATPIF1
1	31342312	31381480	SDC3
1	31838099	31845923	FABP3
1	33476825	33502512	AK2
1	36921361	36930040	MRPS15
1	39328161	39339050	MYCBP
1	39491966	39500308	NDUFS5
1	40538381	40563142	PPT1
1	40766162	40782981	COL9A2
1	42642209	42800903	FOXJ3
1	46805848	46830824	NSUN4
1	47799468	47844511	CMPK1
1	52254865	52344609	NRD1

1	52485803	52521843	TXNDC12
1	53392900	53517289	SCP2
1	54359860	54376759	DIO1
1	55271735	55307937	C1orf177
1	55315299	55352921	DHCR24
1	58946390	59012446	OMA1
1	59246462	59249785	JUN
1	63906440	63988944	ITGB3BP
1	65613231	65697828	AK4
1	68894506	68915642	RPE65
1	70876900	70905534	CTH
1	74663895	75010116	FPGT-TNNI3K
1	75171171	75199092	CRYZ
1	84864214	84880691	DNASE2B
1	89401455	89458643	CCBL2
1	100652477	100715409	DBT
1	108677343	108742980	SLC25A24
1	109756514	109780804	SARS
1	110943876	110950546	LAMTOR5
1	111991742	112004525	ATP5F1
1	113454469	113498975	SLC16A1
1	145516164	145523732	PEX11B
1	149871154	149872348	BOLA1
1	150039341	150117505	VPS45
1	150459839	150480085	TARS2
1	150547026	150552214	MCL1
1	150618700	150669672	GOLPH3L
1	151732122	151736040	MRPL9
1	151745954	151763010	TDRKH
1	151843342	151882361	THEM4
1	152850797	152857523	SMCP
1	154245038	154248355	HAX1
1	154934773	154943223	SHC1
1	155178489	155183624	MTX1
1	156707093	156710923	MRPL24
1	156830670	156851642	NTRK1
1	160007256	160040051	KCNJ10
1	160085519	160113374	ATP1A2
1	160175124	160185162	PEA15
1	161087861	161090984	NIT1

1	161171936	161184184	NDUFS2
1	161195832	161200407	TOMM40L
1	161274524	161279762	MPZ
1	161284165	161334535	SDHC
1	165693527	165738159	TMCO1
1	167885912	167906307	MPC2
1	169433148	169455208	SLC19A2
1	174982093	174992591	MRPS14
1	181002560	181031074	MR1
1	182350838	182360539	GLUL
1	182992594	183114727	LAMC1
1	185703682	186160085	HMCN1
1	186640943	186649559	PTGS2
1	193065594	193074608	GLRX2
1	196743929	196763203	CFHR3
1	201328135	201346828	TNNT2
1	202909959	202927524	ADIPOR1
1	203148058	203155922	CHI3L1
1	203595914	203713209	ATP2B4
1	206138910	206155074	FAM72A
1	209848669	209849735	G0S2
1	209958967	209979520	IRF6
1	211649863	211666259	RD3
1	216676587	216896814	ESRRG
1	219347191	219386207	LYPLAL1
1	220141941	220220000	EPRS
1	220267454	220321383	IARS2
1	220701567	220837799	MARK1
1	220921675	220957596	MARK2
1	224370909	224381142	DEGS1
1	225589203	225615815	LBR
1	226107576	226112040	PYCR2
1	226548391	226595801	PARP1
1	228294379	228297013	MRPL55
1	228395830	228566575	OBSCN
1	229566992	229569843	ACTA1
1	231950371	231954263	DISC2
1	237205701	237997288	RYR2
1	241660856	241683085	FH
1	244998638	245008359	COX20

1	246703862	246729565	TFB2M
1	247579457	247612406	NLRP3
1	1309109	1310562	AURKAIP1
1	45794913	45806142	MUTYH
1	45965855	45976739	MMACHC
1	76190042	76229355	ACADM
1	115247084	115259515	NRAS
1	20301923	20306932	PLA2G2A
1	26210676	26232993	STMN1
1	160160284	160171676	CASQ1
1	231762560	232177019	DISC1
1	19923470	19956315	MINOS1
1	26126666	26144713	SEPN1
1	40306705	40349177	TRIT1
1	151819576	151826173	THEM5
1	153920147	153931132	CRTC2
1	228327928	228336655	GUK1
1	155158299	155162706	MUC1
2	3592690	3605940	RNASEH1
2	6988439	7005950	CMPK2
2	7017795	7038363	RSAD2
2	10580507	10588453	ODC1
2	25383721	25391559	POMC
2	26413503	26467594	HADHA
2	27532359	27545969	MPV17
2	30670136	30867091	LCLAT1
2	32288679	32382706	SPAST
2	32390909	32449181	SLC30A6
2	37458773	37476303	NDUFAF7
2	42577644	42588988	COX7A2L
2	44395999	44458611	PPM1B
2	44502596	44547962	SLC3A1
2	44544747	44589001	PREPL
2	45879042	46415129	PRKCE
2	46524540	46613842	EPAS1
2	48010220	48034092	MSH6
2	54342409	54532435	ACYP2
2	55463755	55496384	MTIF2
2	55861197	55921045	PNPT1
2	69623244	69664760	NFU1

2	71336805	71357394	MCEE
2	71558884	71662191	ZNF638
2	73114511	73119289	SPR
2	73300509	73307147	RAB11FIP5
2	73461363	73480150	CCT7
2	73612885	73837046	ALMS1
2	73867849	73869537	NAT8
2	74425689	74442424	MTHFD2
2	74448560	74542152	SLC4A5
2	74588280	74607482	DCTN1
2	86066270	86116157	ST3GAL5
2	86333304	86369280	PTCD3
2	86371054	86422893	IMMT
2	86441119	86564777	REEP1
2	88422507	88427650	FABP1
2	88991175	89050452	RPIA
2	96068447	96078879	FAHD2A
2	96915945	96931751	TMEM127
2	98262520	98264657	COX5B
2	99215785	99224981	COA5
2	99771417	99779613	LIPT1
2	99797541	99816020	MRPL30
2	109335936	109402267	RANBP2
2	111878490	111926022	BCL2L11
2	113587336	113594356	IL1B
2	119981383	120023227	STEAP3
2	120517206	120742474	PTPN4
2	128175995	128186822	PROC
2	150426146	150444330	MMADHC
2	157292899	157442915	GPD2
2	172290760	172341562	DCAF17
2	176040985	176046391	ATP5G3
2	177134122	177202753	MTX2
2	182540832	182545392	NEUROD1
2	191069359	191184771	HIBCH
2	191745546	191830270	GLS
2	198256697	198299771	SF3B1
2	198318230	198339851	COQ10B
2	198570027	198573114	MARS2
2	200820039	200828847	C2orf47

2	201754049	201768655	NIF3L1
2	202047620	202086383	CASP10
2	202122753	202152434	CASP8
2	211052715	211090215	ACADL
2	216903110	216946539	PECR
2	219135114	219211516	PNKD
2	220283098	220291461	DES
2	224822120	224832431	MRPL44
2	227596032	227663506	IRS1
2	228192227	228222552	MFF
2	228549925	228582745	SLC19A3
2	232573234	232578250	PTMA
2	238232654	238322850	COL6A3
2	241808161	241818536	AGXT
2	242615156	242626383	DTYMK
2	242674029	242708231	D2HGDH
2	152341852	152591001	NEB
2	242045513	242088919	PASK
2	242750159	242758739	NEU4
2	96455401	96463594	GPAT2
2	200775978	200792996	C2orf69
2	179390716	179672150	TTN
3	3168599	3190706	TRNT1
3	3191316	3221401	CRBN
3	4402828	4508966	SUMF1
3	4535031	4889524	ITPR1
3	8775485	8788451	CAV3
3	9791627	9799089	OGG1
3	10183318	10195354	VHL
3	11314009	11599139	ATG7
3	11831918	11888352	TAMM41
3	12045861	12233532	SYN2
3	12393000	12475855	PPARG
3	12625099	12705700	RAF1
3	15090018	15106816	MRPS25
3	15643254	15687325	BTD
3	16306666	16347594	OXNAD1
3	33038099	33138694	GLB1
3	37284681	37408370	GOLGA4
3	38080695	38164228	DLEC1

3	38537762	38567796	EXOG
3	41240941	41281939	CTNNB1
3	42132745	42267382	TRAK1
3	43732374	43764217	ABHD5
3	44379943	44450940	TCAIM
3	45430074	45590328	LARS2
3	45636322	45722755	LIMD1
3	46411632	46417697	CCR5
3	46899356	46904973	MYL3
3	47021172	47051194	NBEAL2
3	49454210	49460111	AMT
3	50367216	50378367	RASSF1
3	50606908	50622421	HEMK1
3	52109248	52188706	POC1A
3	52255095	52265247	TLR9
3	52558384	52567793	NT5DC2
3	57231943	57234280	HESX1
3	58413356	58419579	PDHB
3	59735035	61237133	FHIT
3	69068977	69101484	TMF1
3	93776765	93782067	DHFRL1
3	98298289	98312455	CPOX
3	100053561	100074478	NIT2
3	100082302	100120242	TOMM70A
3	100428385	100467811	TFG
3	107761940	107809935	CD47
3	110790605	110856407	PVRL3
3	111697722	111712215	ABHD10
3	113847556	113897899	DRD3
3	119421868	119485949	MAATS1
3	119540801	119582452	GSK3B
3	120315127	120321258	NDUFB4
3	128198264	128212030	GATA2
3	128444978	128533641	RAB7A
3	138153414	138197256	ESYT3
3	138663065	138665982	FOXL2
3	140660661	140698785	SLC25A36
3	141043054	141168632	ZBTB38
3	148709194	148745456	GYG1
3	155480400	155524055	C3orf33

3	155544300	155572248	SLC33A1
3	169482397	169482848	TERC
3	170714136	170744768	SLC2A2
3	172223297	172241297	TNFSF10
3	172607146	172859058	SPATA16
3	178866310	178952497	PIK3CA
3	179065479	179111008	MFN1
3	179306254	179322434	MRPL47
3	180701497	180707562	DNAJC19
3	184908411	184971886	EHHADH
3	185361526	185542827	IGF2BP2
3	186560462	186576252	ADIPOQ
3	195776154	195809032	TFRC
3	197677051	197682721	RPL35A
3	10327433	10334631	GHRL
3	48506918	48509044	TREX1
3	184032282	184053146	EIF4G1
3	9879532	9885702	RPUSD3
3	9908393	9921938	CIDEC
3	38674525	38687267	SCN5A
3	63884074	63989136	ATXN7
3	149235021	149375812	WWTR1
4	1795038	1810599	FGFR3
4	1813205	1857974	LETM1
4	2061238	2070816	NAT8L
4	6271576	6304992	WFS1
4	7061779	7069800	GRPEL1
4	8368008	8442452	ACOX3
4	17488015	17513857	QDPR
4	23793643	23891700	PPARGC1A
4	24797084	24802467	SOD3
4	38797875	38806412	TLR1
4	39460664	39479271	LIAS
4	40812043	41216635	APBB2
4	41992522	42089551	SLC30A9
4	57302268	57327534	PAICS
4	70894129	70902255	HTN3
4	71859264	71896629	DCK
4	74301932	74321492	AFP
4	74437266	74486348	RASSF6

4	78783804	78873944	MRPL1
4	86936275	87374283	MAPK10
4	88343727	88380606	NUDT9
4	89011415	89080011	ABCG2
4	89178760	89205983	PPM1K
4	89442128	89444952	PYURF
4	103790134	103813963	CISD2
4	103946651	103998170	SLC9B2
4	106067841	106200960	TET2
4	108852716	108874613	CYP2U1
4	123747862	123819390	FGF2
4	123844224	124240604	SPATA5
4	128651554	128695447	SLC25A31
4	146540539	146581187	MMAA
4	152591808	152682175	PET112
4	154605440	154627242	TLR2
4	158141735	158287226	GRIA2
4	159593276	159629841	ETFDH
4	159630278	159644552	PPID
4	169908741	169931468	CBR4
4	170981372	171011183	AADAT
4	185548849	185570629	CASP3
4	185676748	185747215	ACSL1
4	48833322	48863834	OCIAD1
4	108910869	108956331	HADH
4	101944586	102268628	PPP3CA
4	110481354	110608872	CCDC109B
4	113569029	113569713	MIR302B
4	3076407	3245687	HTT
4	140211070	140223705	NDUFC1
4	100257648	100273917	ADH1C
5	204874	218297	CCDC127
5	218355	256814	SDHA
5	271735	315089	PDCD6
5	1253286	1295162	TERT
5	1798498	1799956	MRPL36
5	1801495	1816167	NDUFS6
5	33987090	34008220	AMACR
5	34998205	35048240	AGXT2
5	36876860	37065921	NIPBL

5	38475064	38595507	LIFR
5	41730166	41870791	OXCT1
5	43287571	43313614	HMGCS1
5	54398473	54406080	GZMA
5	54526980	54529508	CCNO
5	56205086	56213165	SETD9
5	65018022	65125111	NLN
5	68513572	68525985	MRPS36
5	70220767	70248842	SMN2
5	71014989	71016875	CARTPT
5	71515235	71616084	MRPS27
5	71616199	71655180	PTCD2
5	74017030	74063042	GFM2
5	78293428	78365449	DMGDH
5	78985658	79096049	CMYA5
5	79950466	80172634	MSH3
5	110074753	110098484	SLC25A46
5	110559946	110820748	CAMK4
5	112073555	112181936	APC
5	112357795	112824527	MCC
5	119800018	120022964	PRR16
5	126626455	126796910	MEGF10
5	131285666	131347355	ACSL6
5	131630144	131679899	SLC22A4
5	131705400	131731306	SLC22A5
5	133307565	133340824	VDAC1
5	135170364	135224326	SLC25A48
5	135364583	135399507	TGFBI
5	137620958	137667516	CDC25C
5	137890570	137911318	HSPA9
5	138855112	138862343	TMEM173
5	140024947	140027370	NDUFA2
5	140071017	140078890	HARS2
5	140682195	140683612	SLC25A2
5	141000442	141016423	HDAC3
5	145138581	145214899	PRELID2
5	145492588	145562294	LARS
5	148206155	148208197	ADRB2
5	148724976	148734146	GRPEL2
5	149109814	149234585	PPARGC1B

5	149599053	149669403	CAMK2A
5	154320632	154348971	MRPL22
5	155753766	156194798	SGCD
5	159436179	159492550	TTC1
5	167913462	167946309	RARS
5	171636649	171710795	UBTD2
5	174905513	174955621	SFXN1
5	176730762	176733960	PRELID1
5	179660594	179719071	MAPK9
5	33440881	33468196	TARS
5	37812778	37835929	GDNF
5	42799981	42812024	SEPP1
6	3076997	3115421	RIPK1
6	3118925	3153432	BPHL
6	4115926	4135831	ECI2
6	5261583	5771816	FARS2
6	6144310	6320924	F13A1
6	7541869	7586946	DSP
6	13574858	13615390	SIRT5
6	13786780	13814792	MCUR1
6	15523031	15663289	DTNBP1
6	18120717	18122851	NHLRC1
6	20534687	21232634	CDKAL1
6	24403152	24425816	MRS2
6	26055967	26056699	HIST1H1C
6	28870778	28891768	TRIM27
6	31496738	31498008	MCCD1
6	31543343	31546112	TNF
6	31745296	31763712	VARS
6	31795511	31798031	HSPA1B
6	33239851	33244281	RPS18
6	35310334	35395968	PPARD
6	35436177	35438558	RPL10A
6	35911290	35992305	SLC26A8
6	39872033	39895455	MOCS1
6	42192668	42419783	TRERF1
6	42664332	42690358	PRPH2
6	43737945	43754223	VEGFA
6	44081372	44095191	MRPL14
6	49398072	49431041	MUT

6	49572889	49604587	RHAG
6	51480144	51952423	PKHD1
6	71276624	71298606	C6orf57
6	74134855	74162043	MB21D1
6	75947390	75953644	COX7A2
6	80816343	81055987	BCKDHB
6	83920109	84140938	ME1
6	84569369	84670146	CYB5R4
6	86323692	86353043	SYNCRIP
6	88224095	88299735	RARS2
6	88849584	88875767	CNR1
6	100836749	100911811	SIM1
6	106534194	106557814	PRDM1
6	106632351	106773695	ATG5
6	107018902	107077373	RTN4IP1
6	107077440	107116292	QRSL1
6	107473760	107780779	PDSS2
6	108616097	108844251	LACE1
6	108882068	109005971	FOXO3
6	109307639	109415708	SESN1
6	110421021	110501207	WASF1
6	123537483	123958238	TRDN
6	126277860	126301389	HINT3
6	127609856	127663552	ECHDC1
6	129204285	129837710	LAMA2
6	131894343	131905472	ARG1
6	136878186	137113656	MAP3K5
6	137518620	137540567	IFNGR1
6	143072603	143266338	HIVEP2
6	145946439	146056991	EPM2A
6	146864827	146876086	RAB32
6	152442818	152958534	SYNE1
6	153308399	153323925	MTRF1L
6	155577263	155635617	TFB1M
6	158402887	158520207	SYNJ2
6	160211491	160219461	MRPL18
6	161123224	161175085	PLG
6	161551056	161695107	AGPAT4
6	161768589	163148834	PARK2
6	170863420	170881958	TBP

6	26096614	26097056	HFE
6	160182988	160200087	ACAT2
6	30882107	30894235	VARS2
6	34204576	34214008	HMGA1
6	43005354	43021683	CUL7
6	45296053	45518819	RUNX2
6	132129155	132216295	ENPP1
6	10723147	10731362	TMEM14C
6	99968869	99981059	TSTD3
6	151186814	151423023	MTHFD1L
6	32146161	32151930	RNF5
6	44213902	44221625	HSP90AB1
6	166778407	166796501	MPC1
7	588833	767313	PRKAR1B
7	1004485	1015235	COX19
7	1022834	1029276	CYP2W1
7	2281856	2290780	NUDT1
7	5632435	5646287	FSCN1
7	10971579	10979813	NDUFA4
7	19155090	19157295	TWIST1
7	24323806	24331484	NPY
7	27565058	27702620	HIBADH
7	30464142	30518393	NOD1
7	30536236	30544457	GGCT
7	30634180	30673648	GARS
7	38299243	38313253	TARP
7	39663151	39747723	RALA
7	40174574	40900366	C7orf10
7	42971938	42977453	MRPL32
7	43678845	43769140	COA1
7	44183869	44198887	GCK
7	44646120	44748669	OGDH
7	45927958	45933267	IGFBP1
7	48128354	48148330	UPP1
7	48211056	48687091	ABCA13
7	56019610	56023033	MRPS17
7	56032269	56067875	GBAS
7	56169265	56174187	CHCHD2
7	66093867	66108216	KCTD7
7	73095247	73097781	DNAJC30

7	73150424	73153190	ABHD11
7	75625654	75677321	STYXL1
7	75677392	75695930	MDH2
7	75931874	75933614	HSPB1
7	77166772	77269388	PTPN12
7	86974950	87029112	CROT
7	91502020	91510016	MTERF
7	92116336	92157845	PEX1
7	95749531	95951459	SLC25A13
7	96745904	96811075	ACN9
7	98625057	98741743	SMURF1
7	100882892	100888371	FIS1
7	102715327	102740210	ARMC10
7	102937872	102955133	PMPCB
7	107531585	107561643	DLD
7	108110865	108166762	PNPLA8
7	116164838	116201239	CAV1
7	117120016	117308718	CFTR
7	121713597	121784344	AASS
7	127220681	127225654	GCC1
7	128470482	128499328	FLNC
7	128594233	128695227	TNPO3
7	129251554	129396922	NRF1
7	129414532	129414609	MIR96
7	132469622	132766828	CHCHD3
7	134212343	134226166	AKR1B10
7	140433812	140624564	BRAF
7	142960521	142966222	GSTK1
7	142985307	143004789	CASP2
7	144149033	144533146	TPK1
7	150688143	150711687	NOS3
7	150778171	150780620	TMUB1
7	158523688	158622319	ESYT2
7	74765875	74789332	WBSCR16
7	127250345	127255780	PAX4
7	42000547	42276618	GLI3
7	45139698	45151346	TBRG4
7	99014361	99063824	ATP5J2-PTCD1
7	102389398	102449672	FAM185A

7	134233848	134264592	AKR1B15
7	150909575	150912320	ABCF2
8	1711869	1734736	CLN8
8	6357174	6420784	ANGPT2
8	11197145	11225961	TDH
8	12940871	13372429	DLC1
8	16884746	16980148	MICU3
8	17433941	17500642	PDGFRL
8	19796581	19824770	LPL
8	24808468	24814383	NEFL
8	27182995	27316908	PTK2B
8	27454433	27472328	CLU
8	29920630	29940649	TMEM66
8	33448853	33457439	DUSP26
8	37620100	37637286	PROSC
8	37820513	37824184	ADRB3
8	38000217	38008600	STAR
8	38034105	38070819	BAG4
8	38457692	38458775	RNF5P1
8	42249278	42263455	VDAC3
8	53535017	53627026	RB1CC1
8	57124314	57131176	CHCHD7
8	66582107	66622798	MTFR1
8	71549500	71581447	LACTB2
8	75146938	75233562	JPH1
8	80831094	80942506	MRPS28
8	86240457	86290342	CA1
8	91013579	91064227	DECR1
8	94929082	94938296	PDP1
8	95384187	95487343	RAD54B
8	96145948	96168913	PLEKHF2
8	97274166	97346774	PTDSS1
8	99114566	99129418	HRSP12
8	109455852	109499136	EMC2
8	109795345	109799770	TMEM74
8	110586404	110657051	SYBU
8	118147336	118188953	SLC30A8
8	121408082	121457647	MRPL13
8	128748314	128753680	MYC
8	140623579	140715299	KCNK9

8	143953772	143961236	CYP11B1
8	143991974	143999259	CYP11B2
8	144391496	144417061	TOP1MT
8	144686082	144691784	PYCRL
8	144989320	145025044	PLEC
8	145064225	145067583	GRINA
8	145149959	145152428	CYC1
8	145597730	145618453	ADCK5
8	145729464	145732555	GPT
8	125323158	125384940	TMEM65
9	4709556	4741309	AK3
9	19115758	19127604	PLIN2
9	21967750	21994490	CDKN2A
9	34458810	34520982	DNAI1
9	34634718	34637768	SIGMAR1
9	35099888	35103154	STOML2
9	35657747	35658015	RMRP
9	35681989	35690053	TPM2
9	37422706	37436986	GRHPR
9	37887593	37904350	SLC25A51
9	38392660	38398662	ALDH1B1
9	75515577	75568233	ALDH1A1
9	79000432	79009444	RFK
9	88879462	88897490	ISCA1
9	90112755	90323549	DAPK1
9	93976096	94124206	AUH
9	97847489	97848370	MIR23B
9	98637899	98731122	ERCC6L2
9	100174301	100258405	TDRD7
9	101978706	101984246	ALG2
9	104182841	104198062	ALDOB
9	107526450	107536291	NIPSNAP3B
9	113006091	113018920	TXN
9	116135697	116138341	HDHD3
9	117781853	117880536	TNC
9	120466452	120479769	TLR4
9	124062078	124095120	GSN
9	124906337	124922098	NDUFA8
9	127640572	127703386	GOLGA1
9	130548304	130553052	CDK9

9	130565153	130576556	FPGS
9	130823511	130829599	NAIF1
9	130830478	130871537	SLC25A25
9	131071395	131084697	TRUB2
9	131580778	131584955	ENDOG
9	135600964	135754198	AK8
9	136528683	136603479	SARDH
9	138392476	138396519	MRPS2
9	139256351	139258263	DNLZ
9	139305115	139318213	PMPCA
9	139388895	139440238	NOTCH1
9	140033608	140063214	GRIN1
9	140446308	140447007	MRPL41
9	36214438	36277053	GNE
9	125026881	125085742	MRRF
9	37588411	37592636	TOMM5
9	131018107	131038268	GOLGA2
9	34329503	34343711	NUDT2
10	1085963	1095061	IDI1
10	12209572	12238143	NUDT5
10	13142081	13180276	OPTN
10	13319795	13342130	PHYH
10	23384426	23410942	MSRB2
10	25305507	25315593	THNSL1
10	27399039	27443349	YME1L1
10	30598729	30638267	MTPAP
10	32297937	32345371	KIF5B
10	43572516	43625797	RET
10	45471708	45474330	C10orf10
10	49609654	49643183	MAPK8
10	60144902	60158990	TFAM
10	62538211	62554610	CDK1
10	63661012	63856707	ARID5B
10	69644426	69678147	SIRT1
10	69865873	69971773	MYPN
10	70242089	70287280	SLC25A16
10	70748476	70776739	KIAA1279
10	70939992	70968849	SUPV3L1
10	71872022	71892690	AIFM2
10	71962585	71993190	PPA1

10	74127083	74385949	MICU1
10	75183336	75193319	MSS51
10	75196562	75255782	PPP3CB
10	75541807	75543406	CHCHD1
10	75572258	75634349	CAMK2G
10	75670861	75677258	PLAU
10	75757871	75879914	VCL
10	75910942	76469061	ADK
10	79734906	79789298	POLR3A
10	81107219	81115089	PPIF
10	82031575	82049434	MAT1A
10	82173574	82192753	FAM213A
10	88809958	88854776	GLUD1
10	89264222	89313218	MINPP1
10	89512874	89577917	ATAD1
10	89623194	89728532	PTEN
10	94211440	94333852	IDE
10	99258767	99330960	UBTD1
10	101156626	101190530	GOT1
10	101370274	101380221	SLC25A28
10	101909846	101945814	ERLIN1
10	102741133	102747272	MRPL43
10	102747292	102754158	C10orf2
10	104590287	104597290	CYP17A1
10	105148808	105156270	USMG5
10	113909621	113943537	GPAM
10	114710008	114927436	TCF7L2
10	120900424	120925204	SFXN4
10	124134093	124191871	PLEKHA1
10	124221040	124274424	HTRA1
10	124739555	124749923	PSTK
10	124768428	124817806	ACADSB
10	126085871	126107545	OAT
10	131934638	131977932	GLRX3
10	133781203	133795435	BNIP3
10	3179918	3215033	PITRM1
10	22605311	22620414	COMMD3-BMI1
10	51565107	51590734	NCOA4
10	51623416	51631894	PARG

10	51371394	51734610	TIMM23
10	115439427	115490668	CASP7
10	76969911	76991207	VDAC2
11	215029	236362	SIRT3
11	450279	491387	PTDSS2
11	494511	506821	RNH1
11	747431	765024	TALDO1
11	787109	790126	CEND1
11	818900	825571	PNPLA2
11	1968501	1977839	MRPL23
11	5246695	5248301	HBB
11	6411643	6416228	SMPD1
11	6502676	6505911	TIMM10B
11	7686325	7694821	CYB5R2
11	8413417	8615503	STK33
11	17298285	17353070	NUCB2
11	17406795	17410206	KCNJ11
11	17414431	17498449	ABCC8
11	17515441	17565963	USH1C
11	17809596	18034637	SERGEF
11	20385288	20405329	HTATIP2
11	27062508	27149354	BBOX1
11	28129797	28355054	METTL15
11	31453948	31531169	IMMP1L
11	34460471	34493607	CAT
11	34903842	34937939	APIP
11	34937676	35017675	PDHX
11	36505316	36531863	TRAF6
11	43902356	43941825	ALKBH3
11	45907046	45928016	MAPK8IP1
11	47600561	47606115	NDUFS3
11	48002109	48192394	PTPRJ
11	58476229	58499447	GLYAT
11	63742078	63744015	COX8A
11	63997752	64001753	DNAJC4
11	64002055	64006736	VEGFB
11	64037299	64052176	BAD
11	64073043	64084210	ESRRA
11	64556608	64570713	MAP4K2
11	64781584	64789657	ARL2

11	65292547	65306182	SCYL1
11	65651210	65656010	FIBP
11	65837823	66012218	PACS1
11	66202549	66206310	MRPL11
11	66615996	66675340	PC
11	67165651	67169376	PPP1CA
11	67351065	67354124	GSTP1
11	67820325	67888858	CHKA
11	68658745	68671303	MRPL21
11	69455872	69469242	CCND1
11	70049268	70053508	FADD
11	73498916	73575656	MRPL48
11	73583712	73587890	COA4
11	73685715	73693889	UCP2
11	73711325	73720282	UCP3
11	78147006	78285909	NARS2
11	82972501	82997377	CCDC90B
11	85358962	85367597	TMEM126A
11	86152149	86383240	ME3
11	92702788	92715948	MTNR1B
11	104896236	104905884	CASP1
11	108093558	108239826	ATM
11	111955538	111957522	TIMM8B
11	111957547	111966525	SDHD
11	112046207	112089649	BCO2
11	112097087	112104695	PTS
11	116706468	116708338	APOA1
11	118955586	118964259	HMBS
11	121322911	121504471	SORL1
11	121986061	121986923	BLID
11	122928199	122932901	HSPA8
11	124617369	124622109	VSIG2
11	124735304	124751370	ROBO3
11	125774260	125793006	DDX25
11	126071988	126081587	RPUSD4
11	134123433	134135746	ACAD8
11	44087728	44105569	ACCS
11	532241	535550	HRAS
11	45868956	45904799	CRY2
11	102217912	102249401	BIRC2

11	118895060	118901616	SLC37A4
11	3876932	4114440	STIM1
11	128805269	128807789	TP53AIP1
11	66313865	66330799	ACTN3
12	389222	498620	KDM5A
12	4758263	4796720	NDUFA9
12	6571403	6579843	VAMP1
12	6976583	6980110	TPI1
12	7023613	7032859	ENO2
12	7801995	7818502	APOBEC1
12	9220303	9268558	A2M
12	12223877	12252627	BCL2L14
12	12870301	12875305	CDKN1B
12	13127798	13153243	HEBP1
12	13714409	14133022	GRIN2B
12	14765567	14849519	GUCY2C
12	21788274	21810789	LDHB
12	25348149	25357949	LYRM5
12	25358179	25403854	KRAS
12	27863705	27909237	MRPS35
12	30862485	30907448	CAPRIN2
12	49688908	49692481	PRPH
12	50497601	50505103	GPD1
12	52908358	52914243	KRT5
12	53497273	53518323	SOAT2
12	53693469	53700965	C12orf10
12	53701239	53715412	AAAS
12	54058943	54070109	ATP5G2
12	55342086	55375622	TESPA1
12	56521985	56538460	ESYT1
12	56660641	56664750	COQ10A
12	56735381	56754037	STAT2
12	56862300	56864767	SPRYD4
12	57623355	57628718	SHMT2
12	58213709	58240747	CTDSP2
12	58335444	58351052	XRCC6BP1
12	59265936	59314319	LRIG3
12	59989820	60183635	SLC16A7
12	66218239	66360071	HMGA2
12	66530716	66563807	TMBIM4

12	68548549	68553521	IFNG
12	69201970	69239320	MDM2
12	70002344	70004942	LRRC10
12	79985744	80084790	PAWR
12	81101407	81103256	MYF6
12	81471808	81649582	ACSS3
12	93861265	93897548	MRPL42
12	98987402	98995778	SLC25A3
12	99039077	99129211	APAF1
12	101549993	101604016	SLC5A8
12	104324111	104341708	HSP90B1
12	105413561	105478341	ALDH1L2
12	106457124	106533811	NUAK1
12	107371068	107380944	MTERFD3
12	109273338	109294710	DAO
12	109535398	109548798	UNG
12	110338078	110355874	TCHP
12	110972236	111021064	PPTC7
12	112204690	112247789	ALDH2
12	112464492	112546635	NAA25
12	113736570	113772925	SLC24A6
12	120881763	120884215	TRIAP1
12	120933858	120936298	DYNLL1
12	120941081	120966964	COQ5
12	121163570	121177811	ACADS
12	121416548	121440314	HNF1A
12	122277432	122296769	HPD
12	123413538	123451056	ABCB9
12	123717843	123742506	C12orf65
12	125262173	125348519	SCARB1
12	125478193	125510349	BRI3BP
12	132379278	132407707	ULK1
12	133264191	133281577	PXMP2
12	51442081	51454207	LETMD1
12	52450277	52453291	NR4A1
12	120532898	120554643	RAB35
12	133287392	133299323	PGAM5
12	7342281	7364079	PEX5
12	24962957	25056009	BCAT1
12	64238540	64541613	SRGAP1

12	108956293	108963160	ISCU
12	109490379	109525831	USP30
12	122692208	122712068	DIABLO
12	7074514	7079916	PHB2
12	58159068	58159468	CYP27B1
12	104609556	104744085	TXNRD1
12	122064454	122079946	ORAI1
12	16500576	16517344	MGST1
13	20761603	20767114	GJB2
13	21750371	21753220	MRP63
13	22066827	22178355	MICU2
13	23902961	24007867	SACS
13	24304327	24463587	MIPEP
13	28494167	28500451	PDX1
13	33590570	33640282	KL
13	41129800	41240734	FOXO1
13	41790515	41837713	MTRF1
13	42140960	42535221	VWA8
13	43787665	44203613	ENOX1
13	46354415	46425846	SIAH3
13	47345390	47371367	ESD
13	48877882	49056026	RB1
13	52506805	52585630	ATP7B
13	52638899	52703214	NEK5
13	80055258	80130212	NDFIP2
13	100258918	100549388	CLYBL
13	110406183	110438914	IRS2
13	111267930	111292342	CARKD
13	111293756	111358480	CARS2
13	47405676	47471211	HTR2A
13	106118564	106143383	DAOA
14	23235730	23240998	OXA1L
14	23299091	23304246	MRPL52
14	23586514	23588820	CEBPE
14	23775970	23795394	BCL2L2-PABPN1
14	23881946	23904870	MYH7
14	24105572	24114848	DHRS2
14	24805226	24809242	RIPK3
14	24908971	24912007	SDR39U1

14	30045686	30396899	PRKD1
14	32030590	32330429	NUBPL
14	35591526	35743497	KIAA0391
14	50709151	50778947	L2HGDH
14	50779046	50792946	ATP5S
14	53503457	53620046	DDHD1
14	55595934	55612148	LGALS3
14	55833108	55878576	ATG14
14	56046924	56151302	KTN1
14	58875369	58894232	TIMM9
14	61438166	61447782	TRMT5
14	62162118	62214977	HIF1A
14	64319682	64693167	SYNE2
14	64699746	64761128	ESR2
14	65541841	65569262	MAX
14	68143518	68162510	RDH11
14	74960422	74962271	ISCA2
14	75480466	75518235	MLH3
14	77731833	77737655	NGB
14	77787229	77797940	GSTZ1
14	92434242	92506403	TRIP11
14	92582467	92588153	NDUFB1
14	93260649	93306304	GOLGA5
14	93648540	93651249	MOAP1
14	96176303	96180533	TCL1A
14	96858447	96955764	AK7
14	97263683	97347951	VRK1
14	100705101	100745371	YY1
14	100757452	100772860	SLC25A29
14	100789678	100796715	SLC25A47
14	102430864	102517135	DYNC1H1
14	102547074	102606086	HSP90AA1
14	103985994	103989196	CKB
14	104163953	104181823	XRCC3
14	105155942	105185947	INF2
14	105235686	105262080	AKT1
14	105780751	105864484	PACS2
14	24836144	24848810	NFATC4
14	101495970	101496051	MIR494
14	94577078	94583033	IFI27

15	25101697	25223729	SNRPN
15	40453209	40513337	BUB1B
15	40697685	40713512	IVD
15	41028085	41047458	RMDN3
15	42651697	42704515	CAPN3
15	43885251	43891604	CKMT1B
15	43985083	43991420	CKMT1A
15	44854893	44955876	SPG11
15	48623620	48635570	DUT
15	50474392	50528589	SLC27A2
15	50569388	50647605	GABPB1
15	50999736	51057910	SPPL2A
15	52401821	52404972	BCL2L10
15	58724174	58861073	LIPC
15	59499014	59500785	LDHAL6B
15	64657210	64673702	KIAA0101
15	65293849	65321977	MTFMT
15	65442783	65477563	CLPX
15	66161796	66184329	RAB11A
15	68499329	68522080	CLN6
15	69070874	69113261	ANP32A
15	72635777	72668520	HEXA
15	74287013	74340155	PML
15	74630102	74660081	CYP11A1
15	75182409	75190565	MPI
15	75212616	75230495	COX5A
15	75639959	75647592	NEIL1
15	75648132	75660968	MAN2C1
15	76508628	76603810	ETFA
15	77223961	77242601	RCN2
15	78441718	78462884	IDH3A
15	80445232	80478924	FAH
15	83211950	83316728	CPEB1
15	89002708	89010633	MRPL46
15	89010683	89021861	MRPS11
15	91073117	91188577	CRTC3
15	99192760	99507759	IGF1R
15	100106132	100256629	MEF2A
15	40987326	41024356	RAD51
15	75931425	75932664	IMP3

15	72491369	72523727	PKM
15	83659370	83680393	C15orf40
15	89346673	89418585	ACAN
15	99797729	99800481	HSP90B2P
16	417383	420569	MRPL28
16	447191	450754	NME4
16	691848	698474	FAM195A
16	772581	776473	CCDC78
16	1494933	1525085	CLCN7
16	1821895	1823140	MRPS34
16	1859103	1877195	HAGH
16	2009516	2011976	NDUFB10
16	2089815	2097867	NTHL1
16	3708037	3767598	TRAP1
16	7382750	7763340	RBFOX1
16	8889036	8891505	TMEM186
16	9847264	10276263	GRIN2A
16	15489610	15503543	MPV17L
16	18794276	18801656	RPS15A
16	20420855	20452281	ACSM5
16	20548082	20587695	ACSM2B
16	20634558	20702578	ACSM1
16	20775311	20808479	ACSM3
16	21529229	21531765	SLC7A5P2
16	23533333	23568696	EARS2
16	23592334	23607639	NDUFAB1
16	23847299	24231932	PRKCB
16	28488599	28503623	CLN3
16	30194730	30200397	CORO1A
16	30435018	30441373	DCTPP1
16	31119614	31124112	BCKDK
16	31439051	31439749	COX6A2
16	46918307	46965201	GPT2
16	48278210	48387407	LONP2
16	53737874	54148379	FTO
16	54317211	54320378	IRX3
16	54965110	54968395	IRX5
16	56485423	56511407	OGFOD1
16	57481336	57495187	COQ9
16	67516473	67517716	AGRP

16	68055176	68057770	DDX28
16	68056846	68113226	DUS2L
16	68119268	68263162	NFATC3
16	68771194	68869444	CDH1
16	69362523	69364498	PDF
16	70147528	70195184	PDPR
16	70286296	70323412	AARS
16	71600753	71610998	TAT
16	74705752	74734789	MLKL
16	78133326	79246564	WWOX
16	81115551	81129980	GCSH
16	83932729	83949787	MLYCD
16	84155743	84178800	HSDL1
16	85812230	85833148	EMC8
16	85833172	85840607	COX4I1
16	86612114	86615304	FOXL1
16	88709696	88717492	CYBA
16	89574804	89624174	SPG7
16	20462858	20498991	ACSM2A
16	20912074	20936328	LYRM1
16	31212806	31214097	PYCARD
16	89160216	89222171	ACSF3
16	781564	782746	NARFL
16	67207755	67209640	NOL3
17	685512	695741	RNMTL1
17	900356	905390	TIMM22
17	1628124	1641893	WDR81
17	2496922	2588909	PAFAH1B1
17	2592679	2614927	CLUH
17	3379295	3402700	ASPA
17	4840425	4843462	SLC25A11
17	6659155	6678964	XAF1
17	7338761	7340998	TMEM102
17	7571719	7590868	TP53
17	7905987	7923658	GUCY2D
17	8191081	8198170	SLC25A35
17	10583648	10600885	SCO1
17	13972718	14111996	COX10
17	15133095	15164093	PMP22
17	16284366	16286054	UBB

17	17104308	17109646	PLD6
17	17115526	17140502	FLCN
17	17206679	17250977	NT5M
17	17408876	17495017	PEMT
17	17584786	17714765	RAI1
17	18231186	18266856	SHMT1
17	19552063	19580908	ALDH3A2
17	21030240	21095285	DHRS7B
17	26205339	26220409	LYRM9
17	27182042	27188072	ERAL1
17	28521336	28562986	SLC6A4
17	28575212	28619184	BLMH
17	29226000	29233286	TEFM
17	29421944	29704695	NF1
17	30469472	30552746	RHOT1
17	33307516	33332088	LIG3
17	34198495	34207377	CCL5
17	35441926	35766902	ACACA
17	36046433	36105096	HNF1B
17	37092684	37123655	FBXO47
17	37793332	37820454	STARD3
17	37856253	37884915	ERBB2
17	39968961	39979469	FKBP10
17	40465342	40540513	STAT3
17	42982993	42992920	GFAP
17	45900637	45908907	MRPL10
17	46125685	46138907	NFE2L1
17	46970147	46973232	ATP5G1
17	47481419	47492242	PHB
17	48172638	48188733	PDK2
17	48445227	48450562	MRPL27
17	48556189	48563336	RSAD1
17	48624428	48633213	SPATA20
17	49230896	49249105	NME1-NME2
17	56347216	56358296	MPO
17	56378587	56406152	BZRAP1
17	56597610	56606778	4-Sep
17	56769962	56811692	RAD51C
17	61554421	61575741	ACE
17	61678230	61685725	TACO1

17	62120389	62207502	ERN1
17	63524682	63557740	AXIN2
17	64298925	64806862	PRKCA
17	66970772	67057136	ABCA9
17	72838167	72856007	GRIN2C
17	73008779	73017356	ICT1
17	73126319	73127890	NT5C
17	73257748	73262457	MRPS7
17	73269060	73285530	SLC25A19
17	73937588	73975515	ACOX1
17	78183078	78194199	SGSH
17	78518624	78940173	RPTOR
17	79213110	79215098	C17orf89
17	79801033	79818544	P4HB
17	79869814	79876058	SIRT7
17	79890268	79894968	PYCR1
17	79993756	79995573	DCXR
17	80036213	80056106	FASN
17	6920933	6921341	MIR497
17	12894928	12921381	ELAC2
17	36886509	36891858	CISD3
17	40947164	40950743	COA3
17	53038479	53046064	COX11
17	79762009	79771889	GCGR
17	1421282	1466110	PITPNA
17	18012019	18083116	MYO15A
17	78234659	78372581	RNF213
18	2655885	2805015	SMCHD1
18	9475529	9538106	RALBP1
18	9708227	9862553	RAB31
18	12254317	12277594	CIDEA
18	12328942	12377275	AFG3L2
18	19321544	19450912	MIB1
18	21111462	21166581	NPC1
18	29339658	29340843	SLC25A52
18	43427573	43547305	EPG5
18	48494386	48514490	ELAC1
18	49866541	51062273	DCC
18	55212072	55253969	FECH
18	55313658	55470327	ATP8B1

18	57567191	57571538	PMAIP1
18	58038563	58040001	MC4R
18	71920526	71959251	CYB5A
18	72909277	72921281	ZADH2
18	13663345	13726591	FAM210A
18	71815745	71826204	TIMM21
18	48556582	48611411	SMAD4
19	852290	856246	ELANE
19	1205797	1228434	STK11
19	1241748	1244824	ATP5D
19	2476122	2478257	GADD45B
19	3762664	3767563	MRPL54
19	4522543	4535208	PLIN5
19	5678432	5680911	C19orf70
19	6361462	6368915	CLPP
19	6372443	6375261	ALKBH7
19	7112265	7294011	INSR
19	7587495	7598895	MCOLN1
19	7733971	7735340	RETN
19	9945882	9960365	PIN1
19	10420890	10426691	FDX1L
19	10812111	10824043	QTRT1
19	10828728	10942586	DNM2
19	11039423	11040916	C19orf52
19	11453451	11456981	TMEM205
19	11616730	11639987	ECSIT
19	12848305	12859137	ASNA1
19	12907633	12912724	PRDX2
19	13215713	13227563	TRMT1
19	13988062	13991571	NANOS3
19	18496967	18499986	GDF15
19	19174802	19223841	SLC25A42
19	29698166	29704136	UQCRFS1
19	30156326	30166383	PLEKHF1
19	30189792	30205963	C19orf12
19	33369903	33462869	CEP89
19	33877854	34012799	PEPD
19	34856031	34893318	GPI
19	36014268	36019253	SBSN
19	36024313	36036221	GAPDHS

19	36316273	36342895	NPHS1
19	38924339	39078204	RYR1
19	39421593	39423659	MRPS12
19	40736223	40791302	AKT2
19	41197433	41222790	ADCK4
19	41305333	41314346	EGLN2
19	41836811	41859831	TGFB1
19	42905665	42931578	LIPE
19	44047463	44079730	XRCC1
19	45394476	45406946	TOMM40
19	45409038	45412650	APOE
19	45809670	45826235	CKM
19	46031024	46088122	OPA3
19	46272975	46285815	DMPK
19	47724078	47734451	BBC3
19	48898131	48948188	GRIN2D
19	49259147	49261582	FGF21
19	49298318	49314320	BCAT2
19	49468565	49470136	FTL
19	49471381	49496610	GYS1
19	50168398	50177173	BCL2L12
19	50410083	50432799	NUP62
19	51461886	51472047	KLK6
19	52249022	52255150	FPR1
19	55555691	55574585	RDH13
19	55663135	55668957	TNNI3
19	55861069	55866463	COX6B2
19	56511091	56573174	NLRP5
19	3522953	3536755	FZR1
19	18794424	18893143	CRTC1
19	50706884	50813801	MYH14
19	10244021	10305755	DNMT1
19	13317255	13617274	CACNA1A
19	39405903	39421536	SARS2
19	1104648	1106787	GPX4
20	2639040	2644865	IDH3B
20	3026674	3028896	MRPS26
20	3127164	3140556	FASTKD5
20	4666796	4682234	PRNP
20	6748744	6760910	BMP2

20	17949761	17971762	MGME1
20	18568555	18744560	DTD1
20	23614293	23618574	CST3
20	25056071	25063015	VSX1
20	30252260	30310656	BCL2L1
20	30407177	30422500	MYLK2
20	30946146	31027122	ASXL1
20	32263291	32274210	E2F1
20	32290549	32308136	PXMP4
20	34203808	34208965	SPAG4
20	34287231	34288902	ROMO1
20	35520226	35580246	SAMHD1
20	43248162	43280376	ADA
20	43570770	43589114	TOMM34
20	43595119	43708593	STK4
20	43850009	43853099	SEMG2
20	45338278	45364985	SLC2A10
20	49126890	49201086	PTPN1
20	52769987	52790516	CYP24A1
20	54933982	54943718	FAM210B
20	54944444	54967351	AURKA
20	56136136	56141513	PCK1
20	56964174	57026156	VAPB
20	57608199	57617901	SLMO2
20	58438617	58507209	SYCP2
20	60758080	60777810	MTG2
20	61867275	61871854	BIRC7
20	62612430	62664453	PRPF6
20	33464327	33515769	ACSS2
20	55743808	55841707	BMP7
20	42984440	43036115	HNF4A
21	16333555	16437126	NRIP1
21	17962556	17962645	MIR125B2
21	27096790	27107965	ATP5J
21	27107328	27144771	GABPA
21	27252860	27543138	APP
21	30300465	30365277	LTN1
21	34602230	34636820	IFNAR2
21	34860237	34864023	DNAJC28
21	35445822	35515334	MRPS6

21	37507262	37518860	CBR3
21	43919741	44001550	SLC37A1
21	44313377	44329773	NDUFV3
21	45345278	45407475	AGPAT3
21	45553493	45565605	C21orf33
21	47401662	47424963	COL6A1
21	47518032	47552763	COL6A2
21	47744035	47865682	PCNT
21	38123188	38353264	HLCS
22	17618409	17640169	CECR5
22	18043182	18073647	SLC25A18
22	18121349	18213621	BCL2L13
22	18216905	18256808	BID
22	18560685	18573797	PEX26
22	18900286	18924066	PRODH
22	19163087	19166338	SLC25A1
22	19929262	19957498	COMT
22	20228937	20255816	RTN4R
22	21061978	21213100	PI4KA
22	24108020	24110141	CHCHD10
22	29138042	29153496	HSCB
22	29663997	29696515	EWSR1
22	29876180	29887277	NEFH
22	29950797	29977326	NIPSNAP1
22	30163357	30166402	UQCR10
22	31721789	31742249	PATZ1
22	36002810	36013384	MB
22	36585175	36600879	APOL4
22	36622254	36636000	APOL2
22	37309674	37336479	CSF2RB
22	37406899	37415494	TST
22	38203911	38213183	GCAT
22	39077953	39080766	TOMM22
22	41165638	41215392	SLC25A17
22	41253084	41328823	XPNPEP3
22	41488613	41576081	EP300
22	41865128	41924993	ACO2
22	42017294	42060052	XRCC6
22	42229105	42302375	SREBF2
22	42481529	42486888	NDUFA6

22	42949924	42970388	SERHL2
22	43528211	43539403	MCAT
22	44351260	44392412	SAMM50
22	46546498	46639653	PPARA
22	46731297	46753237	TRMU
22	47022647	47075688	GRAMD4
22	50964180	50968514	TYMP
22	51007289	51016894	CPT1B
22	51017386	51021428	CHKB
22	51061181	51066601	ARSA
22	37415790	37425863	MPST
22	37447778	37459430	KCTD17
22	43013845	43040640	CYB5R3
22	19863039	19929359	TXNRD2
22	50639407	50656045	SELO

Supplementary Table 5. Tissues and data-sets included in Mendelian Randomization analysis

**Tissues**	**QTL sources**
Brain Anterior cingulate cortex BA24	GTEx
Brain Caudate basal ganglia	ARIES methylation
Brain Cerebellar Hemisphere	Lloyd-Jones et al. expression
Brain Cerebellum	Westra et al. expression
Brain Cortex	
Brain Frontal Cortex BA9	
Brain Hippocampus	
Brain Hypothalamus	
Brain Nucleus accumbens basal ganglia	
Brain Putamen basal ganglia	
Muscle Skeletal	
Nerve Tibial	
Stomach	
Whole Blood	

Supplementary Table 6

**Novel Mitochondrial gene**	**Gene causing monogenic PD with consistent (>3 CNS regions) co-expression**	**CNS tissues where co-expression observed**	**Associated cell type based enrichment p-values**
**CLN8**	FBXO7	Amygdala, Anterior cingulate cortex (BA24), Caudate (basal ganglia), Cortex, Frontal Cortex (BA9), Hippocampus, Hypothalamus, Nucleus accumbens (basal ganglia)	Oligodendrocytes
**FASN**	ATP13A2	Anterior cingulate cortex (BA24), Caudate (basal ganglia), Cortex, Hypothalamus	Neuron
**MPI**	ATP13A2	Anterior cingulate cortex (BA24), Frontal Cortex (BA9), Nucleus accumbens (basal ganglia), Substantia nigra	Neuron
**MRPL43**	PARK7	Amygdala, Anterior cingulate cortex (BA24), Caudate (basal ganglia), Hypothalamus, Nucleus accumbens (basal ganglia), Putamen (basal ganglia)	Neuron
**MRPS34**	PARK7	Anterior cingulate cortex (BA24), Cortex, Frontal Cortex (BA9), Hippocampus, Hypothalamus, Nucleus accumbens (basal ganglia), Putamen (basal ganglia), Spinal cord (cervical c-1), Substantia nigra	Neuron

Sheet: EWCE Results	
**Column Name**	**Description**
CellType	Cell type name (as per level1class or level2class, depending on which were used for the EWCE analysis).
annotLevel	Identifies whether the cell type is considered a level 1 or 2 class.
p	Probability of cellular enrichment.
fold_change	Expression in the target gene list divided by the mean level of expression in the bootstrap samples.
sd_from_mean	The distance (in standard deviations) of the target list from the mean of the bootstrap samples.
GeneSet	Negative or positive t-statistic.
level1class	Major cell-type classes, corresponding to Taxonomy Rank 4 in the Linnarsson data (Zeisel et al., 2018).
Description	Description of the cluster.
Neurotransmitter	Neurotransmitter associated with level2class cell type, as determined by Zeisel *et al.* using triniarisaiton of the expression of genes coding for neurotransmitter transporters or enzymes crucial to neurotransmitter synthesis.

Supplementary Table 7a.

CellType	annotLevel	p	fold_change	sd_from_mean	GeneSet	level1class	OverallClass	Description
DEINH8	2	0.03829	2.211575484	1.399305763	SMR-identified mitochondrial genes	Peptidergic neurons	Neurons	Inhibitory neurons, hypothalamus
HYPEP5	2	0.04613	2.230031325	1.455143782	SMR-identified mitochondrial genes	Peptidergic neurons	Neurons	Vasopressin-producing cells, hypothalamus
HYPEP6	2	0.05312	1.79688472	0.749793325	SMR-identified mitochondrial genes	Cholinergic and monoaminergic neurons	Neurons	Orexin-producing neurons, hypothalamus
HYPEP4	2	0.05317	1.945787766	1.152850489	SMR-identified mitochondrial genes	Peptidergic neurons	Neurons	Oxytocin-producing cells, hypothalamus
ACNT1	2	0.08102	1.793572178	1.448638316	SMR-identified mitochondrial genes	Astrocytes	Astrocytes	Non-telencephalon astrocytes, Gfap-low
ACMB	2	0.09119	1.853098523	1.062922829	SMR-identified mitochondrial genes	Astrocytes	Astrocytes	Dorsal midbrain Myoc-expressing astrocyte-like
SZNBL	2	0.09339	2.518204251	1.32591765	SMR-identified mitochondrial genes	Non-glutamatergic neuroblasts	Neurons	Neuronal intermidate progenitor cells
CBNBL2	2	0.09404	1.524628521	0.486409594	SMR-identified mitochondrial genes	Cerebellum neurons	Neurons	Neuroblasts, cerebellum
ACTE2	2	0.09665	1.671997458	1.26273775	SMR-identified mitochondrial genes	Astrocytes	Astrocytes	Telencephalon astrocytes, Gfap-low
TEGLU2	2	0.09861	1.354366063	0.514964683	SMR-identified mitochondrial genes	Telencephalon projecting excitatory neurons	Neurons	Excitatory neurons, cerebral cortex
ACTE1	2	0.1077	1.606396726	1.009529447	SMR-identified mitochondrial genes	Astrocytes	Astrocytes	Telencephalon astrocytes, Gfap-high

TEINH15	2	0.111	1.321521782	0.692444903	SMR-identified mitochondrial genes	Telencephalon inhibitory interneurons	Neurons	CGE-derived neurogliaform cells, cortex/hippocampus
TEINH11	2	0.11166	1.351807065	0.860333828	SMR-identified mitochondrial genes	Telencephalon inhibitory interneurons	Neurons	R-LM border Cck interneurons, cortex/hippocampus
TEINH9	2	0.11393	1.378967363	1.083319564	SMR-identified mitochondrial genes	Telencephalon inhibitory interneurons	Neurons	Non-border Cck interneurons, hippocampus
TEINH13	2	0.11946	1.352629249	1.022402522	SMR-identified mitochondrial genes	Telencephalon inhibitory interneurons	Neurons	Trilaminar cells, hippocampus
TEGLU4	2	0.13346	1.290368691	0.950671554	SMR-identified mitochondrial genes	Telencephalon projecting excitatory neurons	Neurons	Excitatory neurons, cerebral cortex
DGNBL1	2	0.13978	1.403008142	0.58270304	SMR-identified mitochondrial genes	Non-glutamatergic neuroblasts	Neurons	Granule neuroblasts, dentate gyrus
MFOL2	2	0.14515	1.698095609	0.852759429	SMR-identified mitochondrial genes	Oligodendrocytes	Oligos	Myelin forming oligodendrocytes (MFOL)
TEINH12	2	0.14641	1.272544336	0.64728303	SMR-identified mitochondrial genes	Telencephalon inhibitory interneurons	Neurons	Non-border Cck interneurons, cortex/hippocampus
TEGLU14	2	0.15931	1.331601852	0.650027817	SMR-identified mitochondrial genes	Telencephalon projecting excitatory neurons	Neurons	Excitatory neurons, cerebral cortex
ACNT2	2	0.16074	1.460394715	0.810316833	SMR-identified mitochondrial genes	Astrocytes	Astrocytes	Non-telencephalon astrocytes, Gfap-high
ACOB	2	0.16979	1.410927711	0.678082107	SMR-identified mitochondrial genes	Astrocytes	Astrocytes	Olfactory astrocytes
TEGLU10	2	0.18029	1.219548915	0.684592362	SMR-identified mitochondrial genes	Telencephalon projecting excitatory neurons	Neurons	Excitatory neurons, cerebral cortex
TEINH10	2	0.18064	1.22937185	0.525696978	SMR-identified mitochondrial genes	Telencephalon inhibitory interneurons	Neurons	R-LM border Cck interneurons, cortex/hippocampus
TEGLU3	2	0.18811	1.20115327	0.531387325	SMR-identified mitochondrial genes	Telencephalon projecting excitatory neurons	Neurons	Excitatory neurons, cerebral cortex
TEGLU11	2	0.19015	1.190716325	0.465440058	SMR-identified mitochondrial genes	Telencephalon projecting excitatory neurons	Neurons	Excitatory neurons, cerebral cortex
TEINH20	2	0.19115	1.237696688	0.680434404	SMR-identified mitochondrial genes	Telencephalon inhibitory interneurons	Neurons	Inhibitory interneurons, hippocampus
MBDOP2	2	0.19259	1.176670372	0.265888015	SMR-identified mitochondrial genes	Cholinergic and monoaminergic neurons	Neurons	Dopaminergic neurons, ventral midbrain (SNc, VTA)
HBSER1	2	0.20713	1.197909842	0.358819517	SMR-identified mitochondrial genes	Cholinergic and monoaminergic neurons	Neurons	Serotonergic neurons, hindbrain
DGNBL2	2	0.20825	1.228138681	0.634138081	SMR-identified mitochondrial genes	Non-glutamatergic neuroblasts	Neurons	Granule neuroblasts, dentate gyrus
TEINH14	2	0.20854	1.181760571	0.451639008	SMR-identified mitochondrial genes	Telencephalon inhibitory interneurons	Neurons	CGE-derived neurogliaform cells Cxcl14+, cortex/hippocampus
TEGLU12	2	0.21348	1.19332642	0.408895945	SMR-identified mitochondrial genes	Telencephalon projecting excitatory neurons	Neurons	Excitatory neurons, cerebral cortex

HYPEP8	2	0.21634	0.973987925	-0.017452239	SMR-identified mitochondrial genes	Peptidergic neurons	Neurons	Peptidergic neurons, hypothalamus
SCINH11	2	0.21659	0.933802186	-0.044288925	SMR-identified mitochondrial genes	Peptidergic neurons	Neurons	Central canal neurons, spinal cord
MFOL1	2	0.22754	1.281237567	0.388210754	SMR-identified mitochondrial genes	Oligodendrocytes	Oligos	Myelin forming oligodendrocytes (MFOL)
TEGLU19	2	0.22774	1.066223688	0.068971813	SMR-identified mitochondrial genes	Telencephalon projecting excitatory neurons	Neurons	Excitatory neurons, cerebral cortex
TEGLU22	2	0.22927	1.140199128	0.285707861	SMR-identified mitochondrial genes	Telencephalon projecting excitatory neurons	Neurons	Excitatory neurons, amygdala
TEGLU1	2	0.23436	1.112078852	0.178042989	SMR-identified mitochondrial genes	Telencephalon projecting excitatory neurons	Neurons	Excitatory neurons, cerebral cortex
MGL3	2	0.23519	1.441281704	0.544679375	SMR-identified mitochondrial genes	Microglia	Immune	Microglia, activated
TEGLU20	2	0.23974	1.103100699	0.140271328	SMR-identified mitochondrial genes	Telencephalon projecting excitatory neurons	Neurons	Excitatory neurons, cerebral cortex
DGGRC1	2	0.24455	1.126473744	0.263001065	SMR-identified mitochondrial genes	Dentate gyrus granule neurons	Neurons	Granule neuroblasts, dentate gyrus
OBNBL2	2	0.24907	1.088366101	0.132805654	SMR-identified mitochondrial genes	Glutamatergic neuroblasts	Neurons	Neuroblasts, olfactory bulb
TEGLU21	2	0.25906	1.099026304	0.163389587	SMR-identified mitochondrial genes	Telencephalon projecting excitatory neurons	Neurons	Excitatory neurons, hippocampus CA1
CBNBL1	2	0.26186	1.081580703	0.12769313	SMR-identified mitochondrial genes	Glutamatergic neuroblasts	Neurons	Neuroblasts, cerebellum
HYPEP7	2	0.26498	1.139325983	0.198360101	SMR-identified mitochondrial genes	Cholinergic and monoaminergic neurons	Neurons	Pmch neurons, hypothalamus
MSN5	2	0.26699	1.122589622	0.273367076	SMR-identified mitochondrial genes	Telencephalon projecting inhibitory neurons	Neurons	Patch D1/D2 neurons, striatum
DEINH1	2	0.27212	1.074107634	0.136308675	SMR-identified mitochondrial genes	Telencephalon inhibitory interneurons	Neurons	Inhibitory neurons, thalamus
TEGLU5	2	0.2953	1.075116867	0.162761882	SMR-identified mitochondrial genes	Telencephalon projecting excitatory neurons	Neurons	Excitatory neurons, cerebral cortex
TEINH19	2	0.29664	1.07353964	0.180356352	SMR-identified mitochondrial genes	Telencephalon inhibitory interneurons	Neurons	Hippocamposeptal projection, cortex/hippocampus
TEGLU9	2	0.30781	1.04270189	0.080850437	SMR-identified mitochondrial genes	Telencephalon projecting excitatory neurons	Neurons	Excitatory neurons, cerebral cortex
MOL1	2	0.31202	1.004939034	0.006477728	SMR-identified mitochondrial genes	Oligodendrocytes	Oligos	Mature oligodendrocytes
TEGLU15	2	0.31743	1.041250914	0.068835327	SMR-identified mitochondrial genes	Telencephalon projecting excitatory neurons	Neurons	Excitatory neurons, cerebral cortex
TEINH17	2	0.32056	1.085299597	0.275858644	SMR-identified mitochondrial genes	Telencephalon inhibitory interneurons	Neurons	Axo-axonic, cortex/hippocampus

DEINH4	2	0.32364	1.009574794	0.014378276	SMR-identified mitochondrial genes	Peptidergic neurons	Neurons	Inhibitory neurons, thalamus
TEINH16	2	0.32657	1.017850095	0.03180881	SMR-identified mitochondrial genes	Telencephalon inhibitory interneurons	Neurons	Ivy and MGE-derived neurogliaform cells, cortex/hippocampus
TEINH2	2	0.32671	0.976617707	-0.033633381	SMR-identified mitochondrial genes	Peptidergic neurons	Neurons	Inhibitory neurons, septal nucleus
MSN2	2	0.33241	1.043995906	0.090312264	SMR-identified mitochondrial genes	Telencephalon projecting inhibitory neurons	Neurons	D2 medium spiny neurons, striatum
TEINH6	2	0.33423	1.082729417	0.214971479	SMR-identified mitochondrial genes	Telencephalon inhibitory interneurons	Neurons	Interneuron-selective interneurons, cortex/hippocampus
TEINH4	2	0.34999	1.030326726	0.077191816	SMR-identified mitochondrial genes	Telencephalon inhibitory interneurons	Neurons	Interneuron-selective interneurons, cortex/hippocampus
HBCHO4	2	0.36647	0.931689732	-0.100939195	SMR-identified mitochondrial genes	Cholinergic and monoaminergic neurons	Neurons	Afferent nuclei of cranial nerves III-V
CBGRC	2	0.36808	0.951132948	-0.06876803	SMR-identified mitochondrial genes	Cerebellum neurons	Neurons	Granule neurons, cerebellum
TEGLU7	2	0.36875	1.017712049	0.042612295	SMR-identified mitochondrial genes	Telencephalon projecting excitatory neurons	Neurons	Excitatory neurons, cerebral cortex
HBADR	2	0.37058	0.910760122	-0.115988014	SMR-identified mitochondrial genes	Cholinergic and monoaminergic neurons	Neurons	Adrenergic cell groups of the medulla
MEGLU14	2	0.37139	0.915130657	-0.104136425	SMR-identified mitochondrial genes	Cholinergic and monoaminergic neurons	Neurons	Glutamatergic projection neurons of the raphe nucleus
MSN6	2	0.37563	1.017093217	0.041300447	SMR-identified mitochondrial genes	Telencephalon projecting inhibitory neurons	Neurons	Matrix D1 neurons, striatum
TEGLU24	2	0.38025	0.993698326	-0.0138154	SMR-identified mitochondrial genes	Telencephalon projecting excitatory neurons	Neurons	Excitatory neurons, hippocampus CA1
TEGLU17	2	0.39315	1.002171288	0.005886392	SMR-identified mitochondrial genes	Telencephalon projecting excitatory neurons	Neurons	Excitatory neurons, cerebral cortex
HBSER2	2	0.4075	0.958338463	-0.081073983	SMR-identified mitochondrial genes	Cholinergic and monoaminergic neurons	Neurons	Serotonergic neurons, hindbrain
TEGLU8	2	0.40856	1.006712909	0.021120047	SMR-identified mitochondrial genes	Telencephalon projecting excitatory neurons	Neurons	Excitatory neurons, cerebral cortex
TEINH7	2	0.41161	1.001427361	0.003140507	SMR-identified mitochondrial genes	Telencephalon inhibitory interneurons	Neurons	Interneuron-selective interneurons, hippocampus
TEGLU23	2	0.4128	0.894105571	-0.116761063	SMR-identified mitochondrial genes	Telencephalon projecting excitatory neurons	Neurons	Excitatory neurons, hippocampus CA3
DEINH6	2	0.41745	0.858131599	-0.158888639	SMR-identified mitochondrial genes	Peptidergic neurons	Neurons	Peptidergic neurons, hypothalamus
TEINH21	2	0.42023	0.812649325	-0.175428425	SMR-identified mitochondrial genes	Telencephalon inhibitory interneurons	Neurons	Sleep-active, long-range projection interneurons, cortex/hippocampus
HBCHO3	2	0.42083	0.890836172	-0.161385846	SMR-identified mitochondrial genes	Cholinergic and monoaminergic neurons	Neurons	Afferent nuclei of cranial nerves VI-XII

MSN1	2	0.43278	0.957804498	-0.092728367	SMR-identified mitochondrial genes	Telencephalon projecting inhibitory neurons	Neurons	D1 medium spiny neurons, striatum
TECHO	2	0.43303	0.713832207	-0.243551454	SMR-identified mitochondrial genes	Cholinergic and monoaminergic neurons	Neurons	Cholinergic interneurons, telencephalon
ACBG	2	0.43563	0.899200737	-0.164953588	SMR-identified mitochondrial genes	Astrocytes	Astrocytes	Bergmann glia
OBNBL3	2	0.43712	0.92530285	-0.131862792	SMR-identified mitochondrial genes	Non-glutamatergic neuroblasts	Neurons	Neuroblasts, olfactory bulb
TEGLU13	2	0.44596	0.907937803	-0.132771495	SMR-identified mitochondrial genes	Telencephalon projecting excitatory neurons	Neurons	Excitatory neurons, cerebral cortex
HBSER3	2	0.44737	0.919081882	-0.111449791	SMR-identified mitochondrial genes	Cholinergic and monoaminergic neurons	Neurons	Serotonergic neurons, hindbrain
TEGLU16	2	0.45335	0.941917377	-0.120329118	SMR-identified mitochondrial genes	Telencephalon projecting excitatory neurons	Neurons	Excitatory neurons, cerebral cortex
MSN3	2	0.45839	0.95403706	-0.110413201	SMR-identified mitochondrial genes	Telencephalon projecting inhibitory neurons	Neurons	D2 medium spiny neurons, striatum
MSN4	2	0.45857	0.968499413	-0.087196747	SMR-identified mitochondrial genes	Telencephalon projecting inhibitory neurons	Neurons	D1 medium spiny neurons, striatum
DEINH2	2	0.46311	0.872446034	-0.182228414	SMR-identified mitochondrial genes	Telencephalon inhibitory interneurons	Neurons	Inhibitory neurons, thalamus
HYPEP3	2	0.4653	0.932889827	-0.156558154	SMR-identified mitochondrial genes	Peptidergic neurons	Neurons	Peptidergic neurons, hypothalamus
PVM1	2	0.4659	0.72116429	-0.316181603	SMR-identified mitochondrial genes	Perivascular macrophages	Immune	Perivascular macrophages
CBPC	2	0.46644	0.663333335	-0.312883011	SMR-identified mitochondrial genes	Cerebellum neurons	Neurons	Purkinje cells
TEGLU6	2	0.46776	0.937854803	-0.159875664	SMR-identified mitochondrial genes	Telencephalon projecting excitatory neurons	Neurons	Excitatory neurons, cerebral cortex
DGGRC2	2	0.47162	0.909230351	-0.163934712	SMR-identified mitochondrial genes	Dentate gyrus granule neurons	Neurons	Granule neurons, dentate gyrus
CBINH2	2	0.47592	0.752230107	-0.254115373	SMR-identified mitochondrial genes	Cerebellum neurons	Neurons	Granular layer interneurons, cerebellum
DECHO1	2	0.47821	0.752146893	-0.305169515	SMR-identified mitochondrial genes	Cholinergic and monoaminergic neurons	Neurons	Cholinergic neurons, septal nucleus, Meissnert and diagonal band
TEGLU18	2	0.47913	0.924814458	-0.157116675	SMR-identified mitochondrial genes	Telencephalon projecting excitatory neurons	Neurons	Excitatory neurons, cerebral cortex
TEINH18	2	0.49799	0.936034834	-0.179070293	SMR-identified mitochondrial genes	Telencephalon inhibitory interneurons	Neurons	Basket and bistratified cells, cortex/hippocampus
MGL1	2	0.50332	0.695299639	-0.361998951	SMR-identified mitochondrial genes	Microglia	Immune	Microglia
PVM2	2	0.50662	0.656274526	-0.38889461	SMR-identified mitochondrial genes	Perivascular macrophages	Immune	Perivascular macrophages, activated

TEINH5	2	0.51433	0.925812704	-0.194299737	SMR-identified mitochondrial genes	Telencephalon inhibitory interneurons	Neurons	Interneuron-selective interneurons, cortex/hippocampus
CBINH1	2	0.51828	0.891724976	-0.221113908	SMR-identified mitochondrial genes	Cerebellum neurons	Neurons	Molecular layer interneurons, cerebellum
MGL2	2	0.52131	0.705624065	-0.38591238	SMR-identified mitochondrial genes	Microglia	Immune	Microglia, activated
HBSER4	2	0.52858	0.768890048	-0.251590277	SMR-identified mitochondrial genes	Cholinergic and monoaminergic neurons	Neurons	Serotonergic neurons, hindbrain
OBNBL1	2	0.52961	0.700923302	-0.267261884	SMR-identified mitochondrial genes	Glutamatergic neuroblasts	Neurons	Neuroblasts, olfactory
MBDOP1	2	0.54567	0.699884666	-0.260654002	SMR-identified mitochondrial genes	Cholinergic and monoaminergic neurons	Neurons	Dopaminergic neurons, periaqueductal grey
TEINH3	2	0.55715	0.85601552	-0.286740977	SMR-identified mitochondrial genes	Peptidergic neurons	Neurons	Inhibitory neurons, telencephalon
HYPEP2	2	0.55759	0.78277142	-0.213186963	SMR-identified mitochondrial genes	Peptidergic neurons	Neurons	Peptidergic neurons, hypothalamus
DEINH7	2	0.56366	0.754101256	-0.34180187	SMR-identified mitochondrial genes	Peptidergic neurons	Neurons	Inhibitory neurons, hypothalamus
SEPNBL	2	0.56456	0.812327733	-0.246755612	SMR-identified mitochondrial genes	Glutamatergic neuroblasts	Neurons	Neuroblasts, septum
TEINH8	2	0.57926	0.795422715	-0.31649145	SMR-identified mitochondrial genes	Telencephalon inhibitory interneurons	Neurons	Interneuron-selective interneurons, hippocampus
DETPH	2	0.59282	0.407884552	-0.359960619	SMR-identified mitochondrial genes	Non-glutamatergic neuroblasts	Neurons	Neuroblast-like, habenula
MEINH1	2	0.61718	0.730044681	-0.406344951	SMR-identified mitochondrial genes	Cerebellum neurons	Neurons	Inhibitory neurons, midbrain
HYPEP1	2	0.68203	0.781217841	-0.47434819	SMR-identified mitochondrial genes	Peptidergic neurons	Neurons	Peptidergic neurons, hypothalamus
COP1	2	0.69794	0.587163893	-0.49823522	SMR-identified mitochondrial genes	Oligodendrocytes	Oligos	Committed oligodendrocytes cells (COP)
HBSER5	2	0.72572	0.670935472	-0.377068254	SMR-identified mitochondrial genes	Cholinergic and monoaminergic neurons	Neurons	Serotonergic neurons, hindbrain
DEINH5	2	0.74563	0.792705969	-0.661303102	SMR-identified mitochondrial genes	Peptidergic neurons	Neurons	Peptidergic neurons, hypothalamus
HBNOR	2	0.77549	0.464989151	-0.492407622	SMR-identified mitochondrial genes	Cholinergic and monoaminergic neurons	Neurons	Noradrenergic neurons of the medulla
MEINH14	2	0.77884	0.599535305	-0.714461693	SMR-identified mitochondrial genes	Peptidergic neurons	Neurons	Inhibitory neurons, midbrain

Supplementary Table 8.

ENSG ID	Module Membership	Module colour	Module size	GO enrichment (FDR-corrected p-values)	Cell-specific enrichment (FDR-corrected p-values)
ENSG00000126775	0.8824	black	769	regulation of cellular macromolecule biosynthetic process GO:2000112 (p-value 3.67e-12), regulation of gene expression GO:0010468 (p-value 1.11e-11), regulation of nitrogen compound metabolic process GO:0051171 (p-value 1.23e-11), regulation of macromolecule biosynthetic process GO:0010556 (p-value 2.67e-11), regulation of cellular biosynthetic process GO:0031326 (p-value 8.18e-11)	void
ENSG00000126775	0.7403	tan	1983	histone modification GO:0016570 (p-value 0.000544), covalent chromatin modification GO:0016569 (p-value 0.00055), peptidyl-lysine modification GO:0018205 (p-value 0.00433), mRNA-containing ribonucleoprotein complex export from nucleus GO:0071427 (p-value 0.00575), mRNA export from nucleus GO:0006406 (p-value 0.00575)	void
ENSG00000126775	0.9325	magenta	480	void	void
ENSG00000126775	0.8144	lightsteelblue1	443	protein modification by small protein conjugation or removal GO:0070647 (p-value 0.000437), proteolysis involved in cellular protein catabolic process GO:0051603 (p-value 0.0115), regulation of nitrogen compound metabolic process GO:0051171 (p-value 0.0161), transcription, DNA-templated GO:0006351 (p-value 0.0209), regulation of gene expression GO:0010468 (p-value 0.0214)	void
ENSG00000126775	0.7918	lightcoral	329	RNA processing GO:0006396 (p-value 8.28e-06), regulation of gene expression GO:0010468 (p-value 2e-04), regulation of nitrogen compound metabolic process GO:0051171 (p-value 0.00185), aromatic compound biosynthetic process GO:0019438 (p-value 0.00207), heterocycle biosynthetic process GO:0018130 (p-value 0.00401)	void
ENSG00000126775	0.8435	maroon	589	regulation of RNA metabolic process GO:0051252 (p-value 3.15e-23), regulation of cellular macromolecule biosynthetic process GO:2000112 (p-value 8.81e-23), transcription, DNA-templated GO:0006351 (p-value 4.21e-22), nucleic acid-templated transcription GO:0097659 (p-value	void

				6.75e-22), regulation of nucleobase-containing compound metabolic process GO:0019219 (p-value 1.88e-21)	
ENSG00000126775	0.8181	lightcyan	954	cell cycle process GO:0022402 (p-value 0.0013), cell cycle GO:0007049 (p-value 0.012), vesicle organization GO:0016050 (p-value 0.0178), Golgi vesicle transport GO:0048193 (p-value 0.0209), sister chromatid segregation GO:0000819 (p-value 0.0268)	Oligodendrocytes in Human brain Module (Geschwind,2010) (p-value 1.217e-07). Oligodendrocytes in Cortex (p-value 6.931e-05).
ENSG00000126775	0.8905	lightgreen	1361	regulation of nitrogen compound metabolic process GO:0051171 (p-value 7.4e-13), regulation of gene expression GO:0010468 (p-value 2.85e-12), regulation of cellular macromolecule biosynthetic process GO:2000112 (p-value 3.55e-12), regulation of cellular biosynthetic process GO:0031326 (p-value 5.62e-12), regulation of macromolecule biosynthetic process GO:0010556 (p-value 6.76e-12)	void
ENSG00000126775	0.8166	black	759	regulation of cellular macromolecule biosynthetic process GO:2000112 (p-value 5.07e-08), aromatic compound biosynthetic process GO:0019438 (p-value 7.87e-08), transcription, DNA-templated GO:0006351 (p-value 9.42e-08), nucleic acid-templated transcription GO:0097659 (p-value 1.32e-07), nucleobase-containing compound biosynthetic process GO:0034654 (p-value 1.46e-07)	void
ENSG00000126775	0.8232	magenta	1143	chromosome segregation GO:0007059 (p-value 0.0177)	void
ENSG00000126775	0.907	plum2	625	tRNA modification GO:0006400 (p-value 4.6e-05), tRNA methylation GO:0030488 (p-value 0.0014), RNA modification GO:0009451 (p-value 0.00348), tRNA processing GO:0008033 (p-value 0.0123)	void
ENSG00000126775	0.8868	royalblue	889	regulation of cellular macromolecule biosynthetic process GO:2000112 (p-value 5.1e-30), regulation of nitrogen compound metabolic process GO:0051171 (p-value 1.06e-28), regulation of macromolecule biosynthetic process GO:0010556 (p-value 2.44e-28), regulation of nucleobase-containing compound metabolic process GO:0019219 (p-value 1.42e-27), transcription, DNA-templated GO:0006351 (p-value 8.01e-27)	void
ENSG00000126775	0.8848	brown	787	nucleobase-containing compound biosynthetic process GO:0034654 (p-value 9.57e-18), aromatic compound biosynthetic process	void

				GO:0019438 (p-value 1.18e-17), regulation of cellular macromolecule biosynthetic process GO:2000112 (p-value 1.6e-17), regulation of nucleobase-containing compound metabolic process GO:0019219 (p-value 2.59e-17), heterocycle biosynthetic process GO:0018130 (p-value 2.66e-17)	
ENSG00000152234	0.8183	red	773	mitochondrion organization GO:0007005 (p-value 4.59e-20), organonitrogen compound metabolic process GO:1901564 (p-value 2.86e-13), mitochondrial translation GO:0032543 (p-value 5.97e-12), positive regulation of ubiquitin-protein ligase activity involved in regulation of mitotic cell cycle transition GO:0051437 (p-value 9.85e-12), negative regulation of ubiquitin-protein ligase activity involved in mitotic cell cycle GO:0051436 (p-value 2.59e-11)	Neuron in Human brain Module (Geschwind,2010) (p-value 1.052e-53). Neuron module in Cortex (p-value 1.239e-09).
ENSG00000152234	0.8757	turquoise	3916	organelle organization GO:0043933 (p-value 5.35e-10), chemical synaptic transmission GO:0099536 (p-value 6.68e-10), mitochondrion organization GO:0061024 (p-value 7.25e-09), protein ubiquitination GO:0006464 (p-value 1.01e-08), protein modification by small protein conjugation or removal GO:0036211 (p-value 1.49e-08)	Neuron in Human brain Module (Geschwind,2010) (p-value 2.599e-108). Neuron module in Cortex (p-value 6.091e-72). Neuron, pyramidal in network from Sugino/Winden (p-value 1.578e-11). Neuron, definite (Cahoy, 2008) (p-value 3.405e-35). Neuron, probably (Cahoy, 2008) (p-value 2.007e-138). Neurons-Cahoy (p-value 0.006484). Neuron_Pyramidal_CA1-External (p-value 1.343e-12). Neuron_Interneuron-External (p-value 2.68e-15). Neuron_Pyramidal_S1-External (p-value 1.828e-07). Neuron_Dopaminergic_SNigra-External (p-value 3.798e-06). Neuron.In1-External (p-value 0.002993). Neuron.Ex2-External (p-value 0.01224). Neuron.Ex4-External (p-value 0.00388). Neuron.Ex7-External (p-value 0.008385).
ENSG00000152234	0.8364	honeydew1	511	regulation of autophagy GO:0010506 (p-value 0.0119), protein modification process GO:0036211 (p-value 0.0191), cellular protein modification process GO:0006464 (p-value 0.0191), macromolecule localization GO:0033036 (p-value 0.0318), cellular localization GO:0051641 (p-value 0.0383)	Neuron, probably (Cahoy, 2008) (p-value 0.0493).
ENSG00000152234	0.9306	darkmagenta	635	small molecule metabolic process GO:0044281 (p-value 2.46e-10), small molecule catabolic process GO:0044282 (p-value 7.81e-09), purine ribonucleoside metabolic process GO:0046128 (p-value 1.19e-08), purine nucleoside metabolic process GO:0042278 (p-value 1.52e-08), ribonucleoside metabolic process GO:0009119 (p-value 1.16e-07)	Neuron_Pyramidal_CA1-External (p-value 0.003974).

ENSG00000152234	0.9419	steelblue	248	organonitrogen compound metabolic process GO:1901564 (p-value 1.22e-09), carboxylic acid metabolic process GO:0019752 (p-value 2.63e-07), purine ribonucleoside monophosphate metabolic process GO:0009167 (p-value 2.53e-06), purine nucleoside monophosphate metabolic process GO:0009126 (p-value 2.68e-06), small molecule metabolic process GO:0044281 (p-value 4.32e-06)	Neuron in Human brain Module (Geschwind,2010) (p-value 0.002428).
ENSG00000152234	0.8609	skyblue2	229	organonitrogen compound metabolic process GO:0006807 (p-value 3.23e-09), ATP metabolic process GO:0046128 (p-value 1.78e-08), nucleoside monophosphate metabolic process GO:0009205 (p-value 2.04e-08), regulation of cellular amino acid metabolic process GO:0010565 (p-value 4.96e-08), nucleoside triphosphate metabolic process GO:0006753 (p-value 6.13e-08)	Astrocytes module in Cortex (p-value 0.02388). Neuron in Human brain Module (Geschwind,2010) (p-value 1.914e-18).
ENSG00000152234	0.9339	steelblue	533	mitochondrion organization GO:0007005 (p-value 1.34e-36), organonitrogen compound metabolic process GO:1901564 (p-value 1.88e-26), purine ribonucleoside triphosphate metabolic process GO:0009205 (p-value 4.45e-23), nucleoside triphosphate metabolic process GO:0009141 (p-value 4.74e-23), ribonucleoside monophosphate metabolic process GO:0009161 (p-value 7.56e-23)	Astrocytes module in Cortex (p-value 0.006839). Neuron in Human brain Module (Geschwind,2010) (p-value 3.415e-58).
ENSG00000152234	0.8818	grey60	1255	mitochondrion organization GO:0007005 (p-value 1.45e-23), organelle organization GO:0006996 (p-value 3.33e-17), cellular component biogenesis GO:0044085 (p-value 1.09e-12), organonitrogen compound metabolic process GO:1901564 (p-value 1.31e-11), ncRNA metabolic process GO:0034660 (p-value 1.54e-11)	Neuron in Human brain Module (Geschwind,2010) (p-value 3.32e-68). Neuron module in Cortex (p-value 1.247e-33). Neuron, pyramidal in network from Sugino/Winden (p-value 2.207e-10).
ENSG00000152234	0.8951	salmon	943	mitochondrion organization GO:0007005 (p-value 4.27e-43), oxidative phosphorylation GO:0006119 (p-value 4.46e-35), mitochondrial ATP synthesis coupled electron transport GO:0042775 (p-value 1.1e-34), ATP synthesis coupled electron transport GO:0042773 (p-value 2.04e-34), purine nucleoside triphosphate metabolic process GO:0009144 (p-value 1.7e-33)	Neuron in Human brain Module (Geschwind,2010) (p-value 0.0464). Neuron_Dopaminergic-External (p-value 1.99e-06).
ENSG00000152234	0.87	darkred	1288	peptide biosynthetic process GO:0043043 (p-value 4.43e-59), translation GO:0006412 (p-value 5.71e-59), organonitrogen compound metabolic process GO:1901564 (p-value 5.38e-56),	Neuron in Human brain Module (Geschwind,2010) (p-value 2.25e-07). Neuron_Dopaminergic-External (p-value 0.008645).

				amide biosynthetic process GO:0043604 (p-value 1.07e-54), protein targeting to ER GO:0045047 (p-value 1.81e-54)	
ENSG00000152234	0.7583	turquoise	1003	chemical synaptic transmission GO:0099536 (p-value 6.38e-19), behavior GO:0007610 (p-value 7.54e-12), single-organism behavior GO:0044708 (p-value 5.04e-11), modulation of synaptic transmission GO:0007268 (p-value 1.35e-09), neuron-neuron synaptic transmission GO:0099537 (p-value 2.55e-09)	Neuron in Human brain Module (Geschwind,2010) (p-value 5.791e-31). Neuron module in Cortex (p-value 1.021e-13). Neuron, definite (Cahoy, 2008) (p-value 1.187e-33). Neuron, probably (Cahoy, 2008) (p-value 9.997e-78). Neurons-Cahoy (p-value 0.001408). Neuron_Interneuron-External (p-value 3.092e-05). Neuron_Pyramidal_S1-External (p-value 1.869e-05). Neuron_Dopaminergic_SNigra-External (p-value 4.159e-05).
ENSG00000152234	0.9105	lightgreen	850	mitochondrion organization GO:0007005 (p-value 3.24e-12), organonitrogen compound metabolic process GO:1901564 (p-value 3.53e-08), translation GO:0006412 (p-value 9.54e-08), organonitrogen compound biosynthetic process GO:1901566 (p-value 1.43e-07), peptide biosynthetic process GO:0043043 (p-value 1.71e-07)	Astrocytes module in Cortex (p-value 0.007316). Neuron in Human brain Module (Geschwind,2010) (p-value 8.846e-63). Neuron module in Cortex (p-value 1.738e-22). Neuron, pyramidal in network from Sugino/Winden (p-value 1.245e-08). Neuron, probably (Cahoy, 2008) (p-value 2.658e-09).
ENSG00000152234	0.7856	brown	2305	organelle organization GO:0061024 (p-value 3.12e-10), protein ubiquitination GO:0032446 (p-value 1.36e-08), protein ubiquitination involved in ubiquitin-dependent protein catabolic process GO:0043161 (p-value 2.25e-08), modification-dependent macromolecule catabolic process GO:0016236 (p-value 2.47e-07), protein modification by small protein conjugation GO:0070647 (p-value 2.84e-07)	Neuron in Human brain Module (Geschwind,2010) (p-value 1.355e-111). Neuron module in Cortex (p-value 5.02e-95). Neuron, pyramidal in network from Sugino/Winden (p-value 3.037e-12). Neuron, definite (Cahoy, 2008) (p-value 8.668e-21). Neuron, probably (Cahoy, 2008) (p-value 6.443e-115). Neuron_Pyramidal_CA1-External (p-value 5.12e-05). Neuron_Interneuron-External (p-value 9.764e-14). Neuron_Pyramidal_S1-External (p-value 0.0003522). Neuron_Dopaminergic_SNigra-External (p-value 5.707e-07). Neuron.In2-External (p-value 0.01071).
ENSG00000110888	0.8482	grey60	512	void	void
ENSG00000110888	0.9253	turquoise	3916	organelle organization GO:0043933 (p-value 5.35e-10), chemical synaptic transmission GO:0099536 (p-value 6.68e-10), mitochondrion organization GO:0061024 (p-value 7.25e-09), protein ubiquitination GO:0006464 (p-value 1.01e-08), protein modification by small protein conjugation or removal GO:0036211 (p-value 1.49e-08)	Neuron in Human brain Module (Geschwind,2010) (p-value 2.599e-108). Neuron module in Cortex (p-value 6.091e-72). Neuron, pyramidal in network from Sugino/Winden (p-value 1.578e-11). Neuron, definite (Cahoy, 2008) (p-value 3.405e-35). Neuron, probably (Cahoy, 2008) (p-value 2.007e-138). Neurons-Cahoy (p-value 0.006484). Neuron_Pyramidal_CA1-External (p-value 1.343e-12). Neuron_Interneuron-External (p-value 2.68e-15). Neuron_Pyramidal_S1-External (p-value 1.828e-07). Neuron_Dopaminergic_SNigra-External (p-value 3.798e-06). Neuron.In1-External (p-value 0.002993). Neuron.Ex2-External (p-value 0.01224). Neuron.Ex4-External (p-value 0.00388). Neuron.Ex7-External (p-value 0.008385).

ENSG00000110888	0.8925	honeydew1	511	regulation of autophagy GO:0010506 (p-value 0.0119), protein modification process GO:0036211 (p-value 0.0191), cellular protein modification process GO:0006464 (p-value 0.0191), macromolecule localization GO:0033036 (p-value 0.0318), cellular localization GO:0051641 (p-value 0.0383)	Neuron, probably (Cahoy, 2008) (p-value 0.0493).
ENSG00000110888	0.8427	lightsteelblue1	443	protein modification by small protein conjugation or removal GO:0070647 (p-value 0.000437), proteolysis involved in cellular protein catabolic process GO:0051603 (p-value 0.0115), regulation of nitrogen compound metabolic process GO:0051171 (p-value 0.0161), transcription, DNA-templated GO:0006351 (p-value 0.0209), regulation of gene expression GO:0010468 (p-value 0.0214)	void
ENSG00000110888	0.766	lightcoral	329	RNA processing GO:0006396 (p-value 8.28e-06), regulation of gene expression GO:0010468 (p-value 2e-04), regulation of nitrogen compound metabolic process GO:0051171 (p-value 0.00185), aromatic compound biosynthetic process GO:0019438 (p-value 0.00207), heterocycle biosynthetic process GO:0018130 (p-value 0.00401)	void
ENSG00000110888	0.8296	darkorange2	537	chemical synaptic transmission GO:0099536 (p-value 2.02e-10), neuron projection development GO:0048699 (p-value 2.32e-06), neuron development GO:0022008 (p-value 8.11e-06), cell projection organization GO:0044763 (p-value 1.18e-05), modulation of synaptic transmission GO:0099537 (p-value 1.3e-05)	Neuron in Human brain Module (Geschwind,2010) (p-value 1.365e-23). Neuron module in Cortex (p-value 1.849e-06). Neuron, definite (Cahoy, 2008) (p-value 1.128e-05). Neuron, probably (Cahoy, 2008) (p-value 2.03e-70). Neuron_Pyramidal_CA1-External (p-value 0.001925). Neuron_Interneuron-External (p-value 8.203e-07). Neuron_Dopaminergic_SNigra-External (p-value 0.000637).
ENSG00000110888	0.7161	darkgreen	1362	neuropeptide signaling pathway GO:0007218 (p-value 2.42e-16), chemical synaptic transmission GO:0099536 (p-value 1.51e-14), cell-cell signaling GO:0007267 (p-value 1.29e-13), nervous system development GO:0007275 (p-value 1.13e-12), forebrain development GO:0035270 (p-value 4.07e-12)	Neuron, definite (Cahoy, 2008) (p-value 0.002389). Neuron, probably (Cahoy, 2008) (p-value 3.179e-11). Neuron_Pyramidal_CA1-External (p-value 0.007086). Neuron_Interneuron-External (p-value 8.569e-13). Neuron_Pyramidal_S1-External (p-value 6.695e-05).
ENSG00000110888	0.8714	lightgreen	1361	regulation of nitrogen compound metabolic process GO:0051171 (p-value 7.4e-13), regulation of gene expression GO:0010468 (p-value 2.85e-12), regulation of cellular macromolecule biosynthetic process GO:2000112 (p-value 3.55e-12), regulation of cellular biosynthetic process GO:0031326 (p-value 5.62e-12), regulation of macromolecule biosynthetic	void

				process GO:0010556 (p-value 6.76e-12)	
ENSG00000110888	0.7956	skyblue	693	protein modification by small protein conjugation or removal GO:0070647 (p-value 1.34e-11), mitotic cell cycle GO:0000278 (p-value 1.09e-10), mitotic cell cycle process GO:1903047 (p-value 2.44e-10), protein modification by small protein conjugation GO:0032446 (p-value 5.05e-10), cell cycle GO:0007049 (p-value 1.45e-08)	Neuron in Human brain Module (Geschwind,2010) (p-value 0.03882). Neuron module in Cortex (p-value 3.379e-11).
ENSG00000110888	0.8526	red	1464	covalent chromatin modification GO:0016569 (p-value 0.000148), organelle organization GO:0006996 (p-value 0.000699), nucleic acid-templated transcription GO:0097659 (p-value 0.00114), nucleobase-containing compound biosynthetic process GO:0034654 (p-value 0.00129), aromatic compound biosynthetic process GO:0019438 (p-value 0.0015)	void
ENSG00000110888	0.9558	darkseagreen4	631	regulation of transcription, DNA-templated GO:0006355 (p-value 0.00521), regulation of cellular macromolecule biosynthetic process GO:2000112 (p-value 0.00583), regulation of nucleic acid-templated transcription GO:1903506 (p-value 0.0067), regulation of RNA biosynthetic process GO:2001141 (p-value 0.0083), regulation of nucleobase-containing compound metabolic process GO:0019219 (p-value 0.0105)	void
ENSG00000110888	0.9064	darkred	1031	covalent chromatin modification GO:0016569 (p-value 2.87e-09), autophagy GO:0006914 (p-value 2.54e-08), vacuole organization GO:0007033 (p-value 1.26e-07), nucleic acid-templated transcription GO:0097659 (p-value 6.26e-07), histone modification GO:0016570 (p-value 7.15e-07)	void
ENSG00000110888	0.9027	ivory	704	endomembrane system organization GO:0010256 (p-value 4.03e-05), intracellular transport GO:0046907 (p-value 0.000101), modification-dependent protein catabolic process GO:0019941 (p-value 0.00012), modification-dependent macromolecule catabolic process GO:0043632 (p-value 0.000179), protein modification by small protein conjugation or removal GO:0070647 (p-value 0.000198)	Neuron in Human brain Module (Geschwind,2010) (p-value 1.295e-32). Neuron module in Cortex (p-value 1.82e-17). Neuron, pyramidal in network from Sugino/Winden (p-value 0.00132). Neuron, probably (Cahoy, 2008) (p-value 6.198e-08).
ENSG00000182372	0.7513	black	945	nervous system development GO:0007399 (p-value 6.02e-13), neurogenesis GO:0022008 (p-value 8.88e-12), ensheathment of neurons GO:0007272 (p-value	Oligodendrocytes in Human brain Module (Geschwind,2010) (p-value 1.442e-183). Oligodendrocytes in Cortex (p-value 2.687e-97). Oligodendrocytes, definite (Cahoy, 2008) (p-value 1.284e-39).

				1.87e-11), axon ensheathment GO:0008366 (p-value 1.87e-11), myelination GO:0042552 (p-value 8.78e-11)	Oligodendrocytes from conservative data set (Lein, 2007) (p-value 3.706e-25). Oligodendrocytes-Cahoy (p-value 1.285e-05). Oligodendrocyte-External (p-value 8.934e-59).
ENSG00000182372	0.8131	brown	862	ensheathment of neurons GO:0007272 (p-value 2.8e-09), axon ensheathment GO:0008366 (p-value 2.8e-09), myelination GO:0042552 (p-value 1.45e-08), neurogenesis GO:0022008 (p-value 3.35e-06), oligodendrocyte differentiation GO:0048709 (p-value 6.76e-06)	Oligodendrocytes in Human brain Module (Geschwind,2010) (p-value 3.216e-141). Oligodendrocytes in Cortex (p-value 3.19e-72). Oligodendrocytes, definite (Cahoy, 2008) (p-value 7.64e-39). Oligodendrocytes from conservative data set (Lein, 2007) (p-value 1.04e-16). Oligodendrocytes-Cahoy (p-value 1.207e-05). Oligodendrocyte-External (p-value 3.665e-46).
ENSG00000182372	0.8432	black	966	ensheathment of neurons GO:0007272 (p-value 5.83e-10), axon ensheathment GO:0008366 (p-value 5.83e-10), myelination GO:0042552 (p-value 2.76e-09), oligodendrocyte differentiation GO:0048709 (p-value 6.78e-09), glial cell differentiation GO:0010001 (p-value 1.57e-07)	Oligodendrocytes in Human brain Module (Geschwind,2010) (p-value 5.044e-145). Oligodendrocytes in Cortex (p-value 6.65e-78). Oligodendrocytes, definite (Cahoy, 2008) (p-value 7.242e-42). Oligodendrocytes from conservative data set (Lein, 2007) (p-value 1.316e-20). Oligodendrocytes-Cahoy (p-value 1.525e-05). Oligodendrocyte-External (p-value 9.772e-49).
ENSG00000182372	0.7903	magenta	576	neurogenesis GO:0022008 (p-value 1.96e-07), nervous system development GO:0007399 (p-value 3.15e-06), generation of neurons GO:0048699 (p-value 1.19e-05), oligodendrocyte differentiation GO:0048709 (p-value 2.55e-05), gliogenesis GO:0042063 (p-value 9.91e-05)	Oligodendrocytes in Human brain Module (Geschwind,2010) (p-value 1.197e-113). Oligodendrocytes in Cortex (p-value 3.52e-56). Oligodendrocytes, definite (Cahoy, 2008) (p-value 3.394e-36). Oligodendrocytes from conservative data set (Lein, 2007) (p-value 1.672e-11). Oligodendrocytes-Cahoy (p-value 0.003876). Oligodendrocyte-External (p-value 5.682e-46).
ENSG00000182372	0.7727	darkgreen	529	oligodendrocyte differentiation GO:0048709 (p-value 2.61e-06), glial cell differentiation GO:0010001 (p-value 6.64e-06), gliogenesis GO:0042063 (p-value 2.22e-05), regulation of gliogenesis GO:0014013 (p-value 0.000256), glial cell development GO:0021782 (p-value 0.00369)	Oligodendrocytes in Human brain Module (Geschwind,2010) (p-value 4.267e-74). Oligodendrocytes in Cortex (p-value 8.137e-33). Oligodendrocytes, definite (Cahoy, 2008) (p-value 2.02e-14). Oligodendrocytes from conservative data set (Lein, 2007) (p-value 4.81e-06). Oligodendrocyte-External (p-value 1.195e-23).
ENSG00000182372	0.6311	red	927	ensheathment of neurons GO:0007272 (p-value 2.64e-09), axon ensheathment GO:0008366 (p-value 2.64e-09), myelination GO:0042552 (p-value 1.26e-08), glial cell differentiation GO:0010001 (p-value 0.000288), glial cell development GO:0021782 (p-value 0.000319)	Oligodendrocytes in Human brain Module (Geschwind,2010) (p-value 2.412e-138). Oligodendrocytes in Cortex (p-value 9.065e-72). Oligodendrocytes, definite (Cahoy, 2008) (p-value 2.822e-33). Oligodendrocytes from conservative data set (Lein, 2007) (p-value 8.197e-15). Oligodendrocytes-Cahoy (p-value 7.322e-06). Oligodendrocyte-External (p-value 2.353e-39).
ENSG00000182372	0.8564	plum	556	ensheathment of neurons GO:0007272 (p-value 0.00018), axon ensheathment GO:0008366 (p-value 0.00018), myelination GO:0042552 (p-value 0.00102)	Oligodendrocytes in Human brain Module (Geschwind,2010) (p-value 2.535e-33). Oligodendrocytes in Cortex (p-value 7.784e-13). Oligodendrocytes from conservative data set (Lein, 2007) (p-value 0.01577). Oligodendrocyte-External (p-value 0.001345).
ENSG00000182372	0.6529	darkgrey	350	homophilic cell adhesion via plasma membrane adhesion molecules GO:0007156 (p-value 0.00286)	void

ENSG00000182372	0.4387	thistle3	181	protein folding GO:0080135 (p-value 3.71e-21), response to unfolded protein GO:1903405 (p-value 2.83e-12), response to topologically incorrect protein GO:1990173 (p-value 1.2e-11), response to heat GO:1904869 (p-value 1.05e-07), response to temperature stimulus GO:1904867 (p-value 4.33e-07)	void
ENSG00000182372	0.8859	yellow	712	ensheathment of neurons GO:0007272 (p-value 1.07e-09), axon ensheathment GO:0008366 (p-value 1.07e-09), myelination GO:0042552 (p-value 5.81e-09), oligodendrocyte differentiation GO:0048709 (p-value 3.2e-08), glial cell differentiation GO:0010001 (p-value 5.67e-07)	Oligodendrocytes in Human brain Module (Geschwind,2010) (p-value 6.413e-167). Oligodendrocytes in Cortex (p-value 2.234e-87). Oligodendrocytes, definite (Cahoy, 2008) (p-value 9.082e-48). Oligodendrocytes from conservative data set (Lein, 2007) (p-value 7.58e-21). Oligodendrocytes-Cahoy (p-value 0.0003963). Oligodendrocyte-External (p-value 2.639e-64).
ENSG00000182372	0.9061	yellow	1198	ensheathment of neurons GO:0007272 (p-value 3.05e-12), axon ensheathment GO:0008366 (p-value 3.05e-12), myelination GO:0042552 (p-value 1.23e-11), glial cell differentiation GO:0010001 (p-value 5.83e-08), oligodendrocyte differentiation GO:0048709 (p-value 5.89e-08)	Oligodendrocytes in Human brain Module (Geschwind,2010) (p-value 9.252e-197). Oligodendrocytes in Cortex (p-value 1.885e-99). Oligodendrocytes, definite (Cahoy, 2008) (p-value 9.783e-41). Oligodendrocytes from conservative data set (Lein, 2007) (p-value 3.077e-27). Oligodendrocytes-Cahoy (p-value 1.204e-06). Oligodendrocyte-External (p-value 3.063e-67).
ENSG00000182372	0.7571	ivory	452	response to stress GO:0006950 (p-value 7.99e-10), negative regulation of cellular process GO:0048523 (p-value 2.17e-09), regulation of gene expression GO:0010468 (p-value 4.73e-09), negative regulation of macromolecule metabolic process GO:0010605 (p-value 1.39e-08), negative regulation of metabolic process GO:0009892 (p-value 4.38e-08)	void
ENSG00000182372	0.9053	magenta	827	ensheathment of neurons GO:0007272 (p-value 6.16e-08), axon ensheathment GO:0008366 (p-value 6.16e-08), myelination GO:0042552 (p-value 2.95e-07), neurogenesis GO:0022008 (p-value 1.76e-05), glial cell differentiation GO:0010001 (p-value 2.08e-05)	Oligodendrocytes in Human brain Module (Geschwind,2010) (p-value 1.711e-145). Oligodendrocytes in Cortex (p-value 2.135e-72). Oligodendrocytes, definite (Cahoy, 2008) (p-value 5.532e-30). Oligodendrocytes from conservative data set (Lein, 2007) (p-value 3.016e-15). Oligodendrocytes-Cahoy (p-value 0.0007593). Oligodendrocyte-External (p-value 1.684e-56).
ENSG00000121671	0.6388	brown	1562	chemical synaptic transmission GO:0007267 (p-value 3.01e-32), cell-cell signaling GO:0007154 (p-value 3.36e-24), modulation of synaptic transmission GO:0097479 (p-value 1.13e-20), regulation of synaptic plasticity GO:0050890 (p-value 1.08e-14), neuron projection development GO:0007399 (p-value 2.8e-14)	Neuron, definite (Cahoy, 2008) (p-value 2.234e-14). Neuron, probably (Cahoy, 2008) (p-value 2.044e-59). Neuron_Pyramidal_CA1-External (p-value 6.516e-08). Neuron_Interneuron-External (p-value 2.097e-06). Neuron_Pyramidal_S1-External (p-value 1.804e-14).
ENSG00000121671	0.7482	white	821	chemical synaptic transmission GO:0099536 (p-value 6.38e-24), cell-cell signaling GO:0007267 (p-	Neuron, definite (Cahoy, 2008) (p-value 1.354e-21). Neuron, probably (Cahoy, 2008) (p-value 3.21e-53).

				value 2.78e-11), regulation of ion transport GO:0043269 (p-value 1.53e-09), regulation of ion transmembrane transport GO:0034765 (p-value 9.23e-09), regulation of transmembrane transport GO:0034762 (p-value 3.01e-08)	Neuron_Pyramidal_CA1-External (p-value 5.863e-10). Neuron_Interneuron-External (p-value 1.714e-06). Neuron_Pyramidal_S1-External (p-value 0.002686). Neuron.Ex1-External (p-value 0.0005357). Neuron.Ex7-External (p-value 0.00153).
ENSG00000121671	0.8489	darkmagenta	635	small molecule metabolic process GO:0044281 (p-value 2.46e-10), small molecule catabolic process GO:0044282 (p-value 7.81e-09), purine ribonucleoside metabolic process GO:0046128 (p-value 1.19e-08), purine nucleoside metabolic process GO:0042278 (p-value 1.52e-08), ribonucleoside metabolic process GO:0009119 (p-value 1.16e-07)	Neuron_Pyramidal_CA1-External (p-value 0.003974).
ENSG00000121671	0.7219	mediumpurple3	510	homophilic cell adhesion via plasma membrane adhesion molecules GO:0007156 (p-value 3.97e-11), cell-cell adhesion via plasma-membrane adhesion molecules GO:0098742 (p-value 1.39e-08), synapse assembly GO:0007416 (p-value 2.5e-08), synapse organization GO:0050808 (p-value 1.37e-06), nervous system development GO:0007399 (p-value 7.14e-06)	Astrocyte-External (p-value 1.132e-07).
ENSG00000121671	0.7543	green	3107	chemical synaptic transmission GO:0010646 (p-value 5.76e-29), cell-cell signaling GO:0098662 (p-value 5.55e-21), modulation of synaptic transmission GO:0099536 (p-value 5.82e-19), nervous system development GO:0048168 (p-value 1.27e-13), neuron projection development GO:0022008 (p-value 2.86e-13)	Neuron, definite (Cahoy, 2008) (p-value 2.335e-08). Neuron, probably (Cahoy, 2008) (p-value 3.502e-44). Neuron_Pyramidal_CA1-External (p-value 3.467e-08). Neuron_Interneuron-External (p-value 1.383e-09). Neuron_Pyramidal_S1-External (p-value 1.147e-11). Neuron_Dopaminergic-External (p-value 6.679e-07).
ENSG00000121671	0.7751	floralwhite	509	regulation of cell communication GO:0010646 (p-value 2.36e-05), regulation of signaling GO:0023051 (p-value 3.01e-05), small GTPase mediated signal transduction GO:0007264 (p-value 3.83e-05), regulation of small GTPase mediated signal transduction GO:0051056 (p-value 4.98e-05), neurogenesis GO:0022008 (p-value 5.73e-05)	Neuron, probably (Cahoy, 2008) (p-value 6.81e-10). Neuron_Pyramidal_CA1-External (p-value 0.02027). Neuron_Interneuron-External (p-value 0.02196). Neuron_Pyramidal_S1-External (p-value 0.03544).
ENSG00000121671	0.7225	brown4	257	RNA processing GO:0006396 (p-value 1.72e-05), mRNA metabolic process GO:0016071 (p-value 2.11e-05), regulation of gene expression GO:0010468 (p-value 2.27e-05), RNA biosynthetic process GO:0032774 (p-value 3.35e-05), transcription, DNA-templated GO:0006351 (p-value 4.08e-05)	void
ENSG00000121671	0.7256	lightpink4	266	covalent chromatin modification GO:0016569 (p-value 1.77e-05), chromatin organization	void

				GO:0006325 (p-value 4.13e-05), RNA biosynthetic process GO:0032774 (p-value 0.000169), transcription, DNA-templated GO:0006351 (p-value 0.000323), nucleic acid-templated transcription GO:0097659 (p-value 0.000376)	
ENSG00000121671	0.8612	orangered4	377	void	Neuron_Pyramidal_CA1-External (p-value 0.009451).
ENSG00000121671	0.6438	royalblue	1684	chemical synaptic transmission GO:0071804 (p-value 1e-12), neuron projection development GO:0098662 (p-value 2.82e-09), neuron development GO:0098655 (p-value 2.02e-08), nervous system development GO:0051650 (p-value 2.17e-08), modulation of synaptic transmission GO:0032990 (p-value 7.47e-08)	Neuron, definite (Cahoy, 2008) (p-value 8.77e-05). Neuron, probably (Cahoy, 2008) (p-value 4.431e-51). Neuron_Pyramidal_CA1-External (p-value 0.0002999). Neuron_Interneuron-External (p-value 0.003266). Neuron_Pyramidal_S1-External (p-value 1.302e-12).
ENSG00000121671	0.6386	darkgreen	519	regulation of cellular amino acid metabolic process GO:0031396 (p-value 3.65e-11), regulation of cellular amine metabolic process GO:0044764 (p-value 2.59e-09), positive regulation of protein modification by small protein conjugation or removal GO:1903050 (p-value 3.95e-09), positive regulation of protein ubiquitination GO:2000058 (p-value 3.98e-09), positive regulation of canonical Wnt signaling pathway GO:0060828 (p-value 6.23e-09)	void
ENSG00000121671	0.7384	magenta	1143	chromosome segregation GO:0007059 (p-value 0.0177)	void
ENSG00000121671	0.7904	darkred	1031	covalent chromatin modification GO:0016569 (p-value 2.87e-09), autophagy GO:0006914 (p-value 2.54e-08), vacuole organization GO:0007033 (p-value 1.26e-07), nucleic acid-templated transcription GO:0097659 (p-value 6.26e-07), histone modification GO:0016570 (p-value 7.15e-07)	void
ENSG00000101412	0.5905	skyblue3	338	void	void
ENSG00000101412	0.5038	lightcyan	835	vesicle organization GO:0016050 (p-value 0.0202), single-organism organelle organization GO:1902589 (p-value 0.0208)	Neuron module in Cortex (p-value 0.04768). Oligodendrocytes in Human brain Module (Geschwind,2010) (p-value 2.635e-10). Oligodendrocytes in Cortex (p-value 0.0001135). Oligodendrocyte-External (p-value 2.487e-09).
ENSG00000101412	0.5639	darkred	1288	peptide biosynthetic process GO:0043043 (p-value 4.43e-59), translation GO:0006412 (p-value 5.71e-59), organonitrogen compound metabolic process GO:1901564 (p-value 5.38e-56), amide biosynthetic process GO:0043604 (p-value 1.07e-54), protein targeting to ER GO:0045047 (p-value 1.81e-54)	Neuron in Human brain Module (Geschwind,2010) (p-value 2.25e-07). Neuron_Dopaminergic-External (p-value 0.008645).
ENSG00000101412	0.5651	lightgreen	850	mitochondrion organization GO:0007005 (p-value 3.24e-12), organonitrogen compound	Astrocytes module in Cortex (p-value 0.007316). Neuron in Human brain Module (Geschwind,2010) (p-value

				metabolic process GO:1901564 (p-value 3.53e-08), translation GO:0006412 (p-value 9.54e-08), organonitrogen compound biosynthetic process GO:1901566 (p-value 1.43e-07), peptide biosynthetic process GO:0043043 (p-value 1.71e-07)	8.846e-63). Neuron module in Cortex (p-value 1.738e-22). Neuron, pyramidal in network from Sugino/Winden (p-value 1.245e-08). Neuron, probably (Cahoy, 2008) (p-value 2.658e-09).
ENSG00000101412	0.7059	plum1	250	anatomical structure development NA (p-value 0.0482)	Oligodendrocytes in Human brain Module (Geschwind,2010) (p-value 2.831e-18). Oligodendrocytes in Cortex (p-value 2.424e-07).
ENSG00000101412	0.6361	green	3107	chemical synaptic transmission GO:0010646 (p-value 5.76e-29), cell-cell signaling GO:0098662 (p-value 5.55e-21), modulation of synaptic transmission GO:0099536 (p-value 5.82e-19), nervous system development GO:0048168 (p-value 1.27e-13), neuron projection development GO:0022008 (p-value 2.86e-13)	Neuron, definite (Cahoy, 2008) (p-value 2.335e-08). Neuron, probably (Cahoy, 2008) (p-value 3.502e-44). Neuron_Pyramidal_CA1-External (p-value 3.467e-08). Neuron_Interneuron-External (p-value 1.383e-09). Neuron_Pyramidal_S1-External (p-value 1.147e-11). Neuron_Dopaminergic-External (p-value 6.679e-07).
ENSG00000101412	0.3875	mediumpurple3	392	mitochondrion organization GO:0007005 (p-value 1.02e-05), mitochondrial respiratory chain complex assembly GO:0033108 (p-value 0.0286)	void
ENSG00000101412	0.7629	bisque4	360	cellular amino acid metabolic process GO:0006520 (p-value 0.0223), oxoacid metabolic process GO:0043436 (p-value 0.0272), organic acid metabolic process GO:0006082 (p-value 0.035)	void
ENSG00000101412	0.5889	antiquewhite4	339	void	Oligodendrocytes in Human brain Module (Geschwind,2010) (p-value 6.053e-07). Oligodendrocytes in Cortex (p-value 0.008201). Oligodendrocyte-External (p-value 5.488e-10).
ENSG00000101412	0.7631	yellow	1198	ensheathment of neurons GO:0007272 (p-value 3.05e-12), axon ensheathment GO:0008366 (p-value 3.05e-12), myelination GO:0042552 (p-value 1.23e-11), glial cell differentiation GO:0010001 (p-value 5.83e-08), oligodendrocyte differentiation GO:0048709 (p-value 5.89e-08)	Oligodendrocytes in Human brain Module (Geschwind,2010) (p-value 9.252e-197). Oligodendrocytes in Cortex (p-value 1.885e-99). Oligodendrocytes, definite (Cahoy, 2008) (p-value 9.783e-41). Oligodendrocytes from conservative data set (Lein, 2007) (p-value 3.077e-27). Oligodendrocytes-Cahoy (p-value 1.204e-06). Oligodendrocyte-External (p-value 3.063e-67).
ENSG00000101412	0.5507	lightsteelblue1	642	RNA processing GO:0006396 (p-value 0.000166), response to mitochondrial depolarisation GO:0098780 (p-value 0.00633), mitophagy in response to mitochondrial depolarization GO:0098779 (p-value 0.00633), ncRNA processing GO:0034470 (p-value 0.00655), macromitophagy GO:0000423 (p-value 0.0186)	void
ENSG00000101412	0.5642	darkgreen	314	SRP-dependent cotranslational protein targeting to membrane GO:0006614 (p-value 3.88e-153), protein targeting to ER GO:0045047 (p-value 4.3e-151), establishment of protein localization to endoplasmic reticulum	void

				GO:0072599 (p-value 8e-149), cotranslational protein targeting to membrane GO:0006613 (p-value 4.06e-148), protein localization to endoplasmic reticulum GO:0070972 (p-value 1.62e-139)	
ENSG00000101412	0.4345	violet	389	void	Oligodendrocytes in Human brain Module (Geschwind,2010) (p-value 5.551e-12). Oligodendrocytes in Cortex (p-value 0.005957).
ENSG00000100393	0.8864	royalblue	1082	regulation of nucleobase-containing compound metabolic process GO:0019219 (p-value 3.47e-11), regulation of RNA metabolic process GO:0051252 (p-value 4.39e-11), regulation of cellular macromolecule biosynthetic process GO:2000112 (p-value 9.71e-11), transcription, DNA-templated GO:0006351 (p-value 1.08e-10), regulation of nitrogen compound metabolic process GO:0051171 (p-value 1.5e-10)	void
ENSG00000100393	0.8869	magenta	623	covalent chromatin modification GO:0016569 (p-value 5.34e-14), histone modification GO:0016570 (p-value 3.44e-13), chromatin organization GO:0006325 (p-value 1.24e-11), nucleic acid-templated transcription GO:0097659 (p-value 6.72e-11), RNA biosynthetic process GO:0032774 (p-value 1.53e-10)	void
ENSG00000100393	0.6454	pink	2223	regulation of nitrogen compound metabolic process GO:0051171 (p-value 7.37e-37), regulation of cellular macromolecule biosynthetic process GO:2000112 (p-value 8.12e-37), regulation of macromolecule biosynthetic process GO:0010556 (p-value 2.08e-36), regulation of nucleobase-containing compound metabolic process GO:0019219 (p-value 5.66e-36), regulation of RNA metabolic process GO:0051252 (p-value 8.12e-36)	Neuron, pyramidal in network from Sugino/Winden (p-value 8.231e-07).
ENSG00000100393	0.9097	red	748	transcription, DNA-templated GO:0006351 (p-value 1.98e-14), nucleic acid-templated transcription GO:0097659 (p-value 3.06e-14), regulation of RNA metabolic process GO:0051252 (p-value 2.37e-13), RNA biosynthetic process GO:0032774 (p-value 3.09e-13), regulation of gene expression GO:0010468 (p-value 6.38e-13)	void
ENSG00000100393	0.8513	lavenderblush3	425	homophilic cell adhesion via plasma membrane adhesion molecules GO:0007156 (p-value 0.00648)	void
ENSG00000100393	0.7476	lightsteelblue1	302	regulation of nucleobase-containing compound metabolic process GO:0019219 (p-value	void

				2.99e-07), regulation of RNA metabolic process GO:0051252 (p-value 6.3e-07), transcription, DNA-templated GO:0006351 (p-value 1.71e-06), regulation of RNA biosynthetic process GO:2001141 (p-value 2e-06), nucleic acid-templated transcription GO:0097659 (p-value 2.13e-06)	
ENSG00000100393	0.8762	royalblue	523	covalent chromatin modification GO:0016569 (p-value 0.00041), peptidyl-lysine modification GO:0018205 (p-value 0.000434), histone modification GO:0016570 (p-value 0.00153)	void
ENSG00000100393	0.8252	cyan	960	regulation of RNA metabolic process GO:0051252 (p-value 9.37e-35), regulation of nucleic acid-templated transcription GO:1903506 (p-value 3.52e-34), nucleic acid-templated transcription GO:0097659 (p-value 8.38e-34), regulation of RNA biosynthetic process GO:2001141 (p-value 8.57e-34), regulation of transcription, DNA-templated GO:0006355 (p-value 1.16e-33)	void
ENSG00000100393	0.7469	royalblue	747	transcription, DNA-templated GO:0006351 (p-value 1.69e-11), RNA biosynthetic process GO:0032774 (p-value 2.21e-11), nucleic acid-templated transcription GO:0097659 (p-value 2.4e-11), regulation of RNA metabolic process GO:0051252 (p-value 2.13e-10), regulation of nucleobase-containing compound metabolic process GO:0019219 (p-value 2.6e-10)	void
ENSG00000100393	0.5984	grey60	1272	homophilic cell adhesion via plasma membrane adhesion molecules GO:0007156 (p-value 0.00516)	void
ENSG00000100393	0.9113	sienna3	789	regulation of gene expression GO:0010468 (p-value 9.02e-11), regulation of RNA metabolic process GO:0051252 (p-value 6.55e-10), RNA biosynthetic process GO:0032774 (p-value 7.16e-10), nucleic acid-templated transcription GO:0097659 (p-value 2.34e-09), regulation of nucleobase-containing compound metabolic process GO:0019219 (p-value 2.8e-09)	void
ENSG00000100393	0.9151	lightgreen	685	regulation of nucleobase-containing compound metabolic process GO:0019219 (p-value 7.09e-08), regulation of RNA metabolic process GO:0051252 (p-value 7.78e-08), regulation of gene expression GO:0010468 (p-value 5.41e-07), nucleic acid-templated transcription GO:0097659 (p-value 1.06e-06), regulation of nucleic	void

				acid-templated transcription GO:1903506 (p-value 1.07e-06)	
ENSG00000100393	0.8184	darkorange	694	regulation of gene expression GO:0010468 (p-value 1.11e-12), regulation of nitrogen compound metabolic process GO:0051171 (p-value 2.35e-10), regulation of macromolecule biosynthetic process GO:0010556 (p-value 9.61e-10), regulation of cellular biosynthetic process GO:0031326 (p-value 1.44e-09), regulation of cellular macromolecule biosynthetic process GO:2000112 (p-value 1.62e-09)	void
ENSG00000169710	0.8856	brown	1562	chemical synaptic transmission GO:0007267 (p-value 3.01e-32), cell-cell signaling GO:0007154 (p-value 3.36e-24), modulation of synaptic transmission GO:0097479 (p-value 1.13e-20), regulation of synaptic plasticity GO:0050890 (p-value 1.08e-14), neuron projection development GO:0007399 (p-value 2.8e-14)	Neuron, definite (Cahoy, 2008) (p-value 2.234e-14). Neuron, probably (Cahoy, 2008) (p-value 2.044e-59). Neuron_Pyramidal_CA1-External (p-value 6.516e-08). Neuron_Interneuron-External (p-value 2.097e-06). Neuron_Pyramidal_S1-External (p-value 1.804e-14).
ENSG00000169710	0.6994	blue	880	chemical synaptic transmission GO:0006836 (p-value 2.82e-11), neuron projection development GO:0030182 (p-value 9.67e-09), neuron development GO:0048699 (p-value 9.68e-08), nervous system development GO:0006996 (p-value 7.18e-07), neuron differentiation GO:0022008 (p-value 3.55e-06)	Astrocytes module in Cortex (p-value 0.0007829). Neuron, definite (Cahoy, 2008) (p-value 1.989e-06). Neuron, probably (Cahoy, 2008) (p-value 3.12e-37). Neuron_Pyramidal_CA1-External (p-value 0.03901). Neuron_Pyramidal_S1-External (p-value 8.846e-09).
ENSG00000169710	0.8463	darkgrey	830	ncRNA metabolic process GO:0034660 (p-value 3.2e-11), RNA processing GO:0006396 (p-value 2.78e-09), ncRNA processing GO:0034470 (p-value 2e-07), tRNA metabolic process GO:0006399 (p-value 2.51e-07), ribonucleoprotein complex biogenesis GO:0022613 (p-value 2.56e-07)	void
ENSG00000169710	0.8575	green	3107	chemical synaptic transmission GO:0010646 (p-value 5.76e-29), cell-cell signaling GO:0098662 (p-value 5.55e-21), modulation of synaptic transmission GO:0099536 (p-value 5.82e-19), nervous system development GO:0048168 (p-value 1.27e-13), neuron projection development GO:0022008 (p-value 2.86e-13)	Neuron, definite (Cahoy, 2008) (p-value 2.335e-08). Neuron, probably (Cahoy, 2008) (p-value 3.502e-44). Neuron_Pyramidal_CA1-External (p-value 3.467e-08). Neuron_Interneuron-External (p-value 1.383e-09). Neuron_Pyramidal_S1-External (p-value 1.147e-11). Neuron_Dopaminergic-External (p-value 6.679e-07).
ENSG00000169710	0.7401	sienna3	509	lipid biosynthetic process GO:0008610 (p-value 0.00232), cholesterol biosynthetic process GO:0006695 (p-value 0.00271), secondary alcohol biosynthetic process GO:1902653 (p-value 0.00323), sterol metabolic process GO:0016125 (p-value 0.00346), cholesterol metabolic process GO:0008203 (p-value 0.00567)	Oligodendrocytes in Human brain Module (Geschwind,2010) (p-value 3.833e-05). Oligodendrocyte-External (p-value 1.247e-12).
ENSG00000169710	0.5796	lightcyan1	338	cytoskeleton organization GO:0007010 (p-value 0.0368)	void

ENSG00000169710	0.6089	black	301	cellular component organization NA (p-value 0.0152), cellular component organization or biogenesis NA (p-value 0.0264)	void
ENSG00000169710	0.8619	pink	476	covalent chromatin modification GO:0016569 (p-value 1.1e-07), chromatin organization GO:0006325 (p-value 3.31e-05), transcription, DNA-templated GO:0006351 (p-value 3.41e-05), heterocycle biosynthetic process GO:0018130 (p-value 4.39e-05), nucleic acid-templated transcription GO:0097659 (p-value 4.41e-05)	void
ENSG00000169710	0.8418	royalblue	1684	chemical synaptic transmission GO:0071804 (p-value 1e-12), neuron projection development GO:0098662 (p-value 2.82e-09), neuron development GO:0098655 (p-value 2.02e-08), nervous system development GO:0051650 (p-value 2.17e-08), modulation of synaptic transmission GO:0032990 (p-value 7.47e-08)	Neuron, definite (Cahoy, 2008) (p-value 8.77e-05). Neuron, probably (Cahoy, 2008) (p-value 4.431e-51). Neuron_Pyramidal_CA1-External (p-value 0.0002999). Neuron_Interneuron-External (p-value 0.003266). Neuron_Pyramidal_S1-External (p-value 1.302e-12).
ENSG00000169710	0.8041	red	1464	covalent chromatin modification GO:0016569 (p-value 0.000148), organelle organization GO:0006996 (p-value 0.000699), nucleic acid-templated transcription GO:0097659 (p-value 0.00114), nucleobase-containing compound biosynthetic process GO:0034654 (p-value 0.00129), aromatic compound biosynthetic process GO:0019438 (p-value 0.0015)	void
ENSG00000169710	0.8887	darkorange2	800	regulation of cellular macromolecule biosynthetic process GO:2000112 (p-value 5.73e-07), regulation of gene expression GO:0010468 (p-value 7.8e-07), intracellular transport GO:0046907 (p-value 9.92e-07), covalent chromatin modification GO:0016569 (p-value 1.63e-06), regulation of macromolecule biosynthetic process GO:0010556 (p-value 2.39e-06)	void
ENSG00000169710	0.8894	darkred	1031	covalent chromatin modification GO:0016569 (p-value 2.87e-09), autophagy GO:0006914 (p-value 2.54e-08), vacuole organization GO:0007033 (p-value 1.26e-07), nucleic acid-templated transcription GO:0097659 (p-value 6.26e-07), histone modification GO:0016570 (p-value 7.15e-07)	void
ENSG00000169710	0.8271	floralwhite	853	autophagy GO:0006914 (p-value 0.000232), vacuole organization GO:0007033 (p-value 0.000435), macromolecule modification GO:0043412 (p-value 0.000958), organelle organization GO:0006996 (p-value 0.00151), ncRNA	void

				metabolic process GO:0034660 (p-value 0.00529)	
ENSG00000131981	0.7161	pink	1225	response to stress GO:0006950 (p-value 9.91e-35), immune system process GO:0002376 (p-value 9.17e-32), response to cytokine GO:0034097 (p-value 8.86e-30), cell surface receptor signaling pathway GO:0007166 (p-value 4.1e-28), response to organic substance GO:0010033 (p-value 1.12e-27)	Microglia in Human brain Module (Geschwind, 2010) (p-value 2.326e-68). Microglia (Type2) module in Cortex (p-value 7.295e-33). Microglia-External (p-value 0.01838). Endothelial-External (p-value 3.821e-08).
ENSG00000131981	0.8198	green	670	small molecule catabolic process GO:0044282 (p-value 3.83e-11), organic acid catabolic process GO:0016054 (p-value 1.86e-10), carboxylic acid catabolic process GO:0046395 (p-value 1.86e-10), oxidation-reduction process GO:0055114 (p-value 6.15e-09), monocarboxylic acid catabolic process GO:0072329 (p-value 5.87e-08)	Astrocytes, highly probable (Cahoy, 2008) (p-value 7.828e-26). Astrocytes module in Cortex (p-value 1.749e-58). Ependymal-External (p-value 0.001523). Astrocyte-External (p-value 5.63e-10).
ENSG00000131981	0.6286	paleturquoise	470	regulation of response to stimulus GO:0048583 (p-value 0.000591), regulation of signal transduction GO:0009966 (p-value 0.000629), anatomical structure morphogenesis GO:0009653 (p-value 0.000825), cell surface receptor signaling pathway GO:0007166 (p-value 0.00121), Ras protein signal transduction GO:0007265 (p-value 0.00125)	Astrocytes, highly probable (Cahoy, 2008) (p-value 0.0001791). Astrocytes module in Cortex (p-value 2.891e-12). Astrocyte-External (p-value 0.0001091).
ENSG00000131981	0.7921	black	1351	anatomical structure morphogenesis GO:0009653 (p-value 1.34e-08), cell surface receptor signaling pathway GO:0007166 (p-value 2.71e-08), regulation of signal transduction GO:0009966 (p-value 3.78e-07), cellular response to chemical stimulus GO:0070887 (p-value 7.16e-07), cellular response to organic substance GO:0071310 (p-value 7.38e-07)	Astrocytes module in Cortex (p-value 9.342e-17). Microglia (Type2) module in Cortex (p-value 0.01187). Oligodendrocytes in Human brain Module (Geschwind,2010) (p-value 5.864e-08). Endothelial-External (p-value 1.748e-05).
ENSG00000131981	0.6549	darkslateblue	237	tissue development GO:0009888 (p-value 1.11e-05), negative regulation of cellular process GO:0048523 (p-value 2.11e-05), animal organ development GO:0048513 (p-value 4.01e-05), regulation of developmental process GO:0050793 (p-value 9.89e-05), cell differentiation GO:0030154 (p-value 0.000143)	Astrocytes, highly probable (Cahoy, 2008) (p-value 0.0003201). Astrocytes module in Cortex (p-value 7.059e-09). Microglia in Human brain Module (Geschwind, 2010) (p-value 0.0001961).
ENSG00000131981	0.7416	darkmagenta	269	regulation of dendritic cell differentiation GO:2001198 (p-value 0.000227), regulation of developmental process GO:0050793 (p-value 0.00114), dendritic cell differentiation GO:0097028 (p-value 0.00124), response to stress GO:0006950 (p-value 0.00189), intrinsic apoptotic	Microglia in Human brain Module (Geschwind, 2010) (p-value 0.007345). Microglia (Type2) module in Cortex (p-value 0.044).

				signaling pathway in response to DNA damage GO:0008630 (p-value 0.00844)	
ENSG00000131981	0.8046	darkseagreen3	262	cell death GO:0008219 (p-value 4.41e-06), localization of cell GO:0051674 (p-value 5e-06), cell motility GO:0048870 (p-value 5e-06), programmed cell death GO:0012501 (p-value 1.63e-05), apoptotic process GO:0006915 (p-value 3.35e-05)	Astrocytes, highly probable (Cahoy, 2008) (p-value 5.841e-05). Astrocytes module in Cortex (p-value 4.554e-09).
ENSG00000131981	0.704	darkgreen	348	small molecule catabolic process GO:0044282 (p-value 4.38e-08), single-organism catabolic process GO:0044712 (p-value 9.91e-07), organonitrogen compound catabolic process GO:1901565 (p-value 4.49e-06), organic acid catabolic process GO:0016054 (p-value 1.77e-05), carboxylic acid catabolic process GO:0046395 (p-value 1.77e-05)	Astrocytes, highly probable (Cahoy, 2008) (p-value 1.702e-41). Astrocytes module in Cortex (p-value 2.355e-29). Astrocytes-Cahoy (p-value 4.791e-11). Astrocyte-External (p-value 3.509e-13).
ENSG00000131981	0.7297	pink	829	response to organic substance GO:0010033 (p-value 1.44e-34), response to stress GO:0006950 (p-value 5.04e-34), cellular response to chemical stimulus GO:0070887 (p-value 6.35e-31), cellular response to organic substance GO:0071310 (p-value 1.6e-30), response to cytokine GO:0034097 (p-value 2.71e-28)	Astrocytes, highly probable (Cahoy, 2008) (p-value 0.001327). Astrocytes module in Cortex (p-value 0.003138). Microglia in Human brain Module (Geschwind, 2010) (p-value 1.779e-68). Microglia (Type2) module in Cortex (p-value 2.723e-37). Microglia-External (p-value 0.04953). Neutrophils-External (p-value 0.0373).
ENSG00000131981	0.8386	pink	973	response to stress GO:0006950 (p-value 5.95e-13), regulation of response to stimulus GO:0048583 (p-value 9.29e-13), regulation of signal transduction GO:0009966 (p-value 5.63e-12), regulation of signaling GO:0023051 (p-value 9.44e-12), regulation of cell communication GO:0010646 (p-value 5.04e-11)	Astrocytes module in Cortex (p-value 4.393e-13). Microglia in Human brain Module (Geschwind, 2010) (p-value 1.388e-14). Microglia (Type2) module in Cortex (p-value 3.484e-13). Oligodendrocytes in Human brain Module (Geschwind,2010) (p-value 0.0001253). Endothelial-External (p-value 1.459e-07).
ENSG00000131981	0.6829	skyblue	361	response to stress GO:0006950 (p-value 0.000223), cellular response to zinc ion GO:0071294 (p-value 0.000694), cellular response to chemical stimulus GO:0070887 (p-value 0.000844), defense response GO:0006952 (p-value 0.00179), regulation of immune system process GO:0002682 (p-value 0.00276)	Astrocytes module in Cortex (p-value 0.005335). Microglia in Human brain Module (Geschwind, 2010) (p-value 0.000206). Microglia (Type2) module in Cortex (p-value 3.227e-14).
ENSG00000131981	0.7819	darkorange2	408	response to stress GO:0006950 (p-value 3.72e-05), immune system process GO:0002376 (p-value 0.0114), response to cytokine GO:0034097 (p-value 0.0131), dendritic cell differentiation GO:0097028 (p-value 0.0157), response to external stimulus GO:0009605 (p-value 0.025)	Astrocytes module in Cortex (p-value 0.0001686). Microglia (Type2) module in Cortex (p-value 2.449e-09).
ENSG00000131981	0.7744	lightgreen	339	response to stress GO:0006950 (p-value 1.85e-14), defense response GO:0006952 (p-value 5.25e-13), inflammatory response	Microglia in Human brain Module (Geschwind, 2010) (p-value 4.155e-51). Microglia (Type2) module in Cortex (p-

				GO:0006954 (p-value 7.65e-13), cellular response to chemical stimulus GO:0070887 (p-value 3.52e-12), response to cytokine GO:0034097 (p-value 5.18e-12)	value 3.852e-18). Endothelial-External (p-value 2.451e-06).
ENSG00000168216	0.8892	ivory	768	RNA splicing, via transesterification reactions GO:0000375 (p-value 3.06e-12), RNA splicing GO:0008380 (p-value 7.79e-12), RNA splicing, via transesterification reactions with bulged adenosine as nucleophile GO:0000377 (p-value 9.69e-12), mRNA splicing, via spliceosome GO:0000398 (p-value 9.69e-12), mRNA processing GO:0006397 (p-value 1.23e-08)	Neuron in Human brain Module (Geschwind,2010) (p-value 2.589e-13). Neuron module in Cortex (p-value 1.705e-11). Neuron, pyramidal in network from Sugino/Winden (p-value 0.001125).
ENSG00000168216	0.8983	lightcyan	972	response to unfolded protein GO:0006986 (p-value 0.000121), protein folding GO:0006457 (p-value 0.000571), response to topologically incorrect protein GO:0035966 (p-value 0.00074), protein transport GO:0015031 (p-value 0.00437), macromolecule modification GO:0043412 (p-value 0.0169)	void
ENSG00000168216	0.7122	plum1	433	cellular catabolic process GO:0044248 (p-value 0.025)	Neuron module in Cortex (p-value 4.525e-09). Neuron, pyramidal in network from Sugino/Winden (p-value 0.02417).
ENSG00000168216	0.8513	yellowgreen	625	regulation of nucleobase-containing compound metabolic process GO:0019219 (p-value 3.16e-17), nucleobase-containing compound biosynthetic process GO:0034654 (p-value 1.24e-16), heterocycle biosynthetic process GO:0018130 (p-value 3.74e-16), aromatic compound biosynthetic process GO:0019438 (p-value 4.09e-16), regulation of cellular macromolecule biosynthetic process GO:2000112 (p-value 2.6e-15)	void
ENSG00000168216	0.8935	pink	2223	regulation of nitrogen compound metabolic process GO:0051171 (p-value 7.37e-37), regulation of cellular macromolecule biosynthetic process GO:2000112 (p-value 8.12e-37), regulation of macromolecule biosynthetic process GO:0010556 (p-value 2.08e-36), regulation of nucleobase-containing compound metabolic process GO:0019219 (p-value 5.66e-36), regulation of RNA metabolic process GO:0051252 (p-value 8.12e-36)	Neuron, pyramidal in network from Sugino/Winden (p-value 8.231e-07).
ENSG00000168216	0.9081	tan	592	macroautophagy GO:0016236 (p-value 8.08e-07), autophagy GO:0006914 (p-value 2.07e-05), intracellular transport GO:0046907 (p-value 5.85e-05), protein localization GO:0008104 (p-value 0.000116), establishment of	Neuron in Human brain Module (Geschwind,2010) (p-value 9.001e-07). Neuron module in Cortex (p-value 3.457e-07). Neuron, pyramidal in network from Sugino/Winden (p-value 0.02704).

				protein localization GO:0045184 (p-value 0.000246)	
ENSG00000168216	0.7941	antiquewhite2	341	organelle organization GO:0006996 (p-value 0.000499), nucleotide-excision repair GO:0006289 (p-value 0.000998), cellular component biogenesis GO:0044085 (p-value 0.00247), macromolecular complex assembly GO:0065003 (p-value 0.00553), cellular component assembly GO:0022607 (p-value 0.00846)	Neuron in Human brain Module (Geschwind,2010) (p-value 5.839e-14).
ENSG00000168216	0.7352	darkorange2	278	cellular macromolecule catabolic process GO:0044265 (p-value 8.86e-06), macromolecule catabolic process GO:0009057 (p-value 2.38e-05), proteolysis involved in cellular protein catabolic process GO:0051603 (p-value 2.53e-05), protein modification by small protein conjugation or removal GO:0070647 (p-value 3.55e-05), cellular protein catabolic process GO:0044257 (p-value 7.31e-05)	Neuron module in Cortex (p-value 1.838e-11). Neuron, pyramidal in network from Sugino/Winden (p-value 0.002464).
ENSG00000168216	0.6967	midnightblue	919	nucleobase-containing compound biosynthetic process GO:0034654 (p-value 3.19e-06), regulation of cellular macromolecule biosynthetic process GO:2000112 (p-value 5.09e-06), regulation of macromolecule biosynthetic process GO:0010556 (p-value 5.93e-06), regulation of nucleobase-containing compound metabolic process GO:0019219 (p-value 9.06e-06), heterocycle biosynthetic process GO:0018130 (p-value 1.21e-05)	void
ENSG00000168216	0.8901	brown4	635	regulation of nucleic acid-templated transcription GO:1903506 (p-value 6.64e-07), regulation of transcription, DNA-templated GO:0006355 (p-value 8.21e-07), regulation of RNA biosynthetic process GO:2001141 (p-value 9.68e-07), transcription, DNA-templated GO:0006351 (p-value 1.65e-06), organic cyclic compound biosynthetic process GO:1901362 (p-value 1.81e-06)	Oligodendrocytes in Human brain Module (Geschwind,2010) (p-value 0.02051).
ENSG00000168216	0.8486	lightcyan	954	cell cycle process GO:0022402 (p-value 0.0013), cell cycle GO:0007049 (p-value 0.012), vesicle organization GO:0016050 (p-value 0.0178), Golgi vesicle transport GO:0048193 (p-value 0.0209), sister chromatid segregation GO:0000819 (p-value 0.0268)	Oligodendrocytes in Human brain Module (Geschwind,2010) (p-value 1.217e-07). Oligodendrocytes in Cortex (p-value 6.931e-05).
ENSG00000168216	0.8881	skyblue3	580	mitochondrion organization GO:0007005 (p-value 1.02e-08), mitochondrial translational elongation GO:0070125 (p-value 2.22e-08), mitochondrial translational termination	void

				GO:0070126 (p-value 2.73e-08), translational termination GO:0006415 (p-value 3.61e-07), translational elongation GO:0006414 (p-value 3.62e-07)	
ENSG00000168216	0.8383	midnightblue	655	transcription, DNA-templated GO:0006351 (p-value 1.17e-21), nucleic acid-templated transcription GO:0097659 (p-value 2.06e-21), RNA biosynthetic process GO:0032774 (p-value 7.52e-21), regulation of nucleic acid-templated transcription GO:1903506 (p-value 5.52e-20), regulation of gene expression GO:0010468 (p-value 6.41e-20)	void
ENSG00000139428	0.659	skyblue3	338	void	void
ENSG00000139428	0.7629	salmon	943	mitochondrion organization GO:0007005 (p-value 4.27e-43), oxidative phosphorylation GO:0006119 (p-value 4.46e-35), mitochondrial ATP synthesis coupled electron transport GO:0042775 (p-value 1.1e-34), ATP synthesis coupled electron transport GO:0042773 (p-value 2.04e-34), purine nucleoside triphosphate metabolic process GO:0009144 (p-value 1.7e-33)	Neuron in Human brain Module (Geschwind,2010) (p-value 0.0464). Neuron_Dopaminergic-External (p-value 1.99e-06).
ENSG00000139428	0.8658	darkred	1288	peptide biosynthetic process GO:0043043 (p-value 4.43e-59), translation GO:0006412 (p-value 5.71e-59), organonitrogen compound metabolic process GO:1901564 (p-value 5.38e-56), amide biosynthetic process GO:0043604 (p-value 1.07e-54), protein targeting to ER GO:0045047 (p-value 1.81e-54)	Neuron in Human brain Module (Geschwind,2010) (p-value 2.25e-07). Neuron_Dopaminergic-External (p-value 0.008645).
ENSG00000139428	0.6418	darkred	1474	mitochondrion organization GO:0007005 (p-value 1.02e-42), ATP synthesis coupled electron transport GO:0042773 (p-value 1.82e-34), respiratory electron transport chain GO:0022904 (p-value 1.29e-33), mitochondrial ATP synthesis coupled electron transport GO:0042775 (p-value 1.76e-33), electron transport chain GO:0022900 (p-value 4.33e-33)	Neuron_Dopaminergic-External (p-value 3.228e-05).
ENSG00000139428	0.6462	lightsteelblue1	642	RNA processing GO:0006396 (p-value 0.000166), response to mitochondrial depolarisation GO:0098780 (p-value 0.00633), mitophagy in response to mitochondrial depolarization GO:0098779 (p-value 0.00633), ncRNA processing GO:0034470 (p-value 0.00655), macromitophagy GO:0000423 (p-value 0.0186)	void
ENSG00000139428	0.7535	darkgrey	830	ncRNA metabolic process GO:0034660 (p-value 3.2e-11), RNA processing GO:0006396 (p-value 2.78e-09), ncRNA processing GO:0034470 (p-value 2e-07), tRNA	void

				metabolic process GO:0006399 (p-value 2.51e-07), ribonucleoprotein complex biogenesis GO:0022613 (p-value 2.56e-07)	
ENSG00000139428	0.8195	greenyellow	1460	SRP-dependent cotranslational protein targeting to membrane GO:0006614 (p-value 6.34e-59), protein targeting to ER GO:0045047 (p-value 1.24e-58), cotranslational protein targeting to membrane GO:0006613 (p-value 4.72e-57), establishment of protein localization to endoplasmic reticulum GO:0072599 (p-value 5.29e-57), protein localization to endoplasmic reticulum GO:0070972 (p-value 1.48e-51)	void
ENSG00000139428	0.8743	sienna3	509	lipid biosynthetic process GO:0008610 (p-value 0.00232), cholesterol biosynthetic process GO:0006695 (p-value 0.00271), secondary alcohol biosynthetic process GO:1902653 (p-value 0.00323), sterol metabolic process GO:0016125 (p-value 0.00346), cholesterol metabolic process GO:0008203 (p-value 0.00567)	Oligodendrocytes in Human brain Module (Geschwind,2010) (p-value 3.833e-05). Oligodendrocyte-External (p-value 1.247e-12).
ENSG00000139428	0.694	darkgreen	291	void	void
ENSG00000139428	0.8633	antiquewhite4	339	void	Oligodendrocytes in Human brain Module (Geschwind,2010) (p-value 6.053e-07). Oligodendrocytes in Cortex (p-value 0.008201). Oligodendrocyte-External (p-value 5.488e-10).
ENSG00000139428	0.8261	darkturquoise	1045	mitochondrion organization GO:0007005 (p-value 9.04e-52), oxidative phosphorylation GO:0006119 (p-value 7.72e-35), ATP synthesis coupled electron transport GO:0042773 (p-value 2.26e-34), respiratory electron transport chain GO:0022904 (p-value 2.88e-34), electron transport chain GO:0022900 (p-value 8.44e-34)	Neuron_Dopaminergic-External (p-value 2.573e-07).
ENSG00000139428	0.8451	salmon4	579	RNA processing GO:0006396 (p-value 9.61e-06), mRNA processing GO:0006397 (p-value 0.000131), ribonucleoprotein complex biogenesis GO:0022613 (p-value 0.000302), RNA splicing GO:0008380 (p-value 0.00113), RNA splicing, via transesterification reactions with bulged adenosine as nucleophile GO:0000377 (p-value 0.00604)	void
ENSG00000139428	0.6797	plum2	518	void	void
ENSG00000178802	0.7084	turquoise	2045	chemical synaptic transmission GO:0023051 (p-value 1.33e-65), cell-cell signaling GO:0051962 (p-value 7.65e-43), modulation of synaptic transmission GO:0051640 (p-value 1.53e-33), nervous system development GO:0048468 (p-value 3.32e-32), neuron projection	Neuron, definite (Cahoy, 2008) (p-value 2.392e-37). Neuron, probably (Cahoy, 2008) (p-value 7.597e-108). Neuron_Pyramidal_CA1-External (p-value 3.115e-15). Neuron_Interneuron-External (p-value 1.456e-20). Neuron_Pyramidal_S1-External (p-value 1.477e-18). Neuron_Dopaminergic-

				development GO:0048699 (p-value 1.11e-25)	External (p-value 9.78e-06). Neuron_Dopaminergic_SNigra-External (p-value 0.004763). Neuron.In6-External (p-value 0.01889). Neuron.Ex1-External (p-value 1.538e-08). Neuron.Ex7-External (p-value 0.0005358).
ENSG00000178802	0.7871	paleturquoise	848	nervous system development GO:0000902 (p-value 1.93e-11), neuron projection development GO:0007399 (p-value 9.25e-11), neurogenesis GO:0030030 (p-value 2.09e-10), generation of neurons GO:0031344 (p-value 7.97e-10), neuron development GO:0000904 (p-value 1.06e-09)	Neuron, definite (Cahoy, 2008) (p-value 0.0005271). Neuron, probably (Cahoy, 2008) (p-value 8.155e-30). Neuron_Pyramidal_CA1-External (p-value 0.009455). Neuron_Interneuron-External (p-value 0.03546). Neuron_Pyramidal_S1-External (p-value 0.004703).
ENSG00000178802	0.8336	salmon	943	mitochondrion organization GO:0007005 (p-value 4.27e-43), oxidative phosphorylation GO:0006119 (p-value 4.46e-35), mitochondrial ATP synthesis coupled electron transport GO:0042775 (p-value 1.1e-34), ATP synthesis coupled electron transport GO:0042773 (p-value 2.04e-34), purine nucleoside triphosphate metabolic process GO:0009144 (p-value 1.7e-33)	Neuron in Human brain Module (Geschwind,2010) (p-value 0.0464). Neuron_Dopaminergic-External (p-value 1.99e-06).
ENSG00000178802	0.7792	darkred	1288	peptide biosynthetic process GO:0043043 (p-value 4.43e-59), translation GO:0006412 (p-value 5.71e-59), organonitrogen compound metabolic process GO:1901564 (p-value 5.38e-56), amide biosynthetic process GO:0043604 (p-value 1.07e-54), protein targeting to ER GO:0045047 (p-value 1.81e-54)	Neuron in Human brain Module (Geschwind,2010) (p-value 2.25e-07). Neuron_Dopaminergic-External (p-value 0.008645).
ENSG00000178802	0.8461	darkorange2	653	catechol-containing compound biosynthetic process GO:0009713 (p-value 1.67e-05), catecholamine biosynthetic process GO:0042423 (p-value 1.67e-05), regulation of neurotransmitter levels GO:0001505 (p-value 7.95e-05), cell-cell signaling GO:0007267 (p-value 0.000107), ion transport GO:0007268 (p-value 0.00014)	Neuron, definite (Cahoy, 2008) (p-value 0.0004005). Neuron, probably (Cahoy, 2008) (p-value 9.33e-20). Neuron_Interneuron-External (p-value 1.336e-09). Neuron_Dopaminergic-External (p-value 4.597e-25). Neuron_Dopaminergic_SNigra-External (p-value 2.425e-14). Neuron.In5-External (p-value 0.02296).
ENSG00000178802	0.6983	lightsteelblue1	642	RNA processing GO:0006396 (p-value 0.000166), response to mitochondrial depolarisation GO:0098780 (p-value 0.00633), mitophagy in response to mitochondrial depolarization GO:0098779 (p-value 0.00633), ncRNA processing GO:0034470 (p-value 0.00655), macromitophagy GO:0000423 (p-value 0.0186)	void
ENSG00000178802	0.8783	darkgrey	830	ncRNA metabolic process GO:0034660 (p-value 3.2e-11), RNA processing GO:0006396 (p-value 2.78e-09), ncRNA processing GO:0034470 (p-value 2e-07), tRNA metabolic process GO:0006399 (p-value 2.51e-07), ribonucleoprotein	void

				complex biogenesis GO:0022613 (p-value 2.56e-07)	
ENSG00000178802	0.8454	green	3107	chemical synaptic transmission GO:0010646 (p-value 5.76e-29), cell-cell signaling GO:0098662 (p-value 5.55e-21), modulation of synaptic transmission GO:0099536 (p-value 5.82e-19), nervous system development GO:0048168 (p-value 1.27e-13), neuron projection development GO:0022008 (p-value 2.86e-13)	Neuron, definite (Cahoy, 2008) (p-value 2.335e-08). Neuron, probably (Cahoy, 2008) (p-value 3.502e-44). Neuron_Pyramidal_CA1-External (p-value 3.467e-08). Neuron_Interneuron-External (p-value 1.383e-09). Neuron_Pyramidal_S1-External (p-value 1.147e-11). Neuron_Dopaminergic-External (p-value 6.679e-07).
ENSG00000178802	0.8804	orangered3	576	ncRNA metabolic process GO:0034660 (p-value 7.01e-06), organelle organization GO:0006996 (p-value 5.93e-05), tRNA metabolic process GO:0006399 (p-value 0.00022), tRNA aminoacylation for protein translation GO:0006418 (p-value 0.00161), RNA processing GO:0006396 (p-value 0.00213)	void
ENSG00000178802	0.9192	greenyellow	360	void	void
ENSG00000178802	0.8408	cyan	305	void	void
ENSG00000178802	0.8555	darkorange	509	RNA processing GO:0006396 (p-value 6.27e-11), ribonucleoprotein complex biogenesis GO:0022613 (p-value 1.55e-09), organonitrogen compound metabolic process GO:1901564 (p-value 1.44e-08), amide biosynthetic process GO:0043604 (p-value 7.77e-08), small molecule metabolic process GO:0044281 (p-value 1.05e-07)	void
ENSG00000178802	0.8689	darkturquoise	1045	mitochondrion organization GO:0007005 (p-value 9.04e-52), oxidative phosphorylation GO:0006119 (p-value 7.72e-35), ATP synthesis coupled electron transport GO:0042773 (p-value 2.26e-34), respiratory electron transport chain GO:0022904 (p-value 2.88e-34), electron transport chain GO:0022900 (p-value 8.44e-34)	Neuron_Dopaminergic-External (p-value 2.573e-07).
ENSG00000055950	0.5604	skyblue3	338	void	void
ENSG00000055950	0.8622	midnightblue	648	mitochondrion organization GO:0007005 (p-value 7.79e-45), mitochondrial ATP synthesis coupled electron transport GO:0042775 (p-value 6.46e-33), ATP synthesis coupled electron transport GO:0042773 (p-value 1.08e-32), respiratory electron transport chain GO:0022904 (p-value 7.31e-31), electron transport chain GO:0022900 (p-value 1.69e-30)	Neuron_Dopaminergic-External (p-value 1.604e-05).
ENSG00000055950	0.8939	salmon	943	mitochondrion organization GO:0007005 (p-value 4.27e-43), oxidative phosphorylation GO:0006119 (p-value 4.46e-35), mitochondrial ATP synthesis coupled electron transport	Neuron in Human brain Module (Geschwind,2010) (p-value 0.0464). Neuron_Dopaminergic-External (p-value 1.99e-06).

				GO:0042775 (p-value 1.1e-34), ATP synthesis coupled electron transport GO:0042773 (p-value 2.04e-34), purine nucleoside triphosphate metabolic process GO:0009144 (p-value 1.7e-33)	
ENSG00000055950	0.861	darkred	1288	peptide biosynthetic process GO:0043043 (p-value 4.43e-59), translation GO:0006412 (p-value 5.71e-59), organonitrogen compound metabolic process GO:1901564 (p-value 5.38e-56), amide biosynthetic process GO:0043604 (p-value 1.07e-54), protein targeting to ER GO:0045047 (p-value 1.81e-54)	Neuron in Human brain Module (Geschwind,2010) (p-value 2.25e-07). Neuron_Dopaminergic-External (p-value 0.008645).
ENSG00000055950	0.7634	greenyellow	616	mRNA metabolic process GO:0016071 (p-value 3.5e-11), mRNA processing GO:0006397 (p-value 1.11e-10), RNA splicing GO:0008380 (p-value 2.26e-10), RNA processing GO:0006396 (p-value 9.13e-10), RNA splicing, via transesterification reactions GO:0000375 (p-value 5.46e-07)	void
ENSG00000055950	0.7024	tan	701	SRP-dependent cotranslational protein targeting to membrane GO:0006614 (p-value 1.44e-111), protein targeting to ER GO:0045047 (p-value 7.49e-109), establishment of protein localization to endoplasmic reticulum GO:0072599 (p-value 1.33e-106), cotranslational protein targeting to membrane GO:0006613 (p-value 1.37e-106), nuclear-transcribed mRNA catabolic process, nonsense-mediated decay GO:0000184 (p-value 2.02e-97)	void
ENSG00000055950	0.8195	lightcyan	533	mitochondrion organization GO:0007005 (p-value 1.48e-34), purine ribonucleoside triphosphate metabolic process GO:0009205 (p-value 5.76e-32), ATP metabolic process GO:0046034 (p-value 9.57e-32), mitochondrial ATP synthesis coupled electron transport GO:0042775 (p-value 1.35e-31), ribonucleoside triphosphate metabolic process GO:0009199 (p-value 1.86e-31)	Neuron_Dopaminergic-External (p-value 1.383e-12).
ENSG00000055950	0.8638	greenyellow	1460	SRP-dependent cotranslational protein targeting to membrane GO:0006614 (p-value 6.34e-59), protein targeting to ER GO:0045047 (p-value 1.24e-58), cotranslational protein targeting to membrane GO:0006613 (p-value 4.72e-57), establishment of protein localization to endoplasmic reticulum GO:0072599 (p-value 5.29e-57), protein localization to endoplasmic reticulum GO:0070972 (p-value 1.48e-51)	void

ENSG00000055950	0.9258	mediumpurple3	392	mitochondrion organization GO:0007005 (p-value 1.02e-05), mitochondrial respiratory chain complex assembly GO:0033108 (p-value 0.0286)	void
ENSG00000055950	0.8173	brown	355	ATP metabolic process GO:0046034 (p-value 1.04e-13), purine nucleoside triphosphate metabolic process GO:0009144 (p-value 2.87e-13), mitochondrion organization GO:0007005 (p-value 6.54e-13), purine ribonucleoside triphosphate metabolic process GO:0009205 (p-value 1.19e-12), ribonucleoside triphosphate metabolic process GO:0009199 (p-value 2.16e-12)	Neuron_Dopaminergic-External (p-value 0.001234).
ENSG00000055950	0.8955	lightsteelblue1	319	mitochondrion organization GO:0007005 (p-value 0.000275), RNA splicing GO:0008380 (p-value 0.00196), RNA splicing, via transesterification reactions with bulged adenosine as nucleophile GO:0000377 (p-value 0.0037), mRNA splicing, via spliceosome GO:0000398 (p-value 0.0037), RNA splicing, via transesterification reactions GO:0000375 (p-value 0.00438)	void
ENSG00000055950	0.9152	darkturquoise	1045	mitochondrion organization GO:0007005 (p-value 9.04e-52), oxidative phosphorylation GO:0006119 (p-value 7.72e-35), ATP synthesis coupled electron transport GO:0042773 (p-value 2.26e-34), respiratory electron transport chain GO:0022904 (p-value 2.88e-34), electron transport chain GO:0022900 (p-value 8.44e-34)	Neuron_Dopaminergic-External (p-value 2.573e-07).
ENSG00000055950	0.8077	bisque4	508	carbohydrate phosphorylation GO:0046835 (p-value 0.0145)	void
ENSG00000074071	0.9071	lightgreen	594	oxidative phosphorylation GO:0006119 (p-value 1.26e-18), mitochondrion organization GO:0007005 (p-value 5.52e-18), mitochondrial ATP synthesis coupled electron transport GO:0042775 (p-value 1.7e-17), ATP synthesis coupled electron transport GO:0042773 (p-value 2.3e-17), respiratory electron transport chain GO:0022904 (p-value 1.26e-16)	Neuron_Dopaminergic-External (p-value 0.0005717).
ENSG00000074071	0.8555	midnightblue	648	mitochondrion organization GO:0007005 (p-value 7.79e-45), mitochondrial ATP synthesis coupled electron transport GO:0042775 (p-value 6.46e-33), ATP synthesis coupled electron transport GO:0042773 (p-value 1.08e-32), respiratory electron transport chain GO:0022904 (p-value 7.31e-31), electron transport	Neuron_Dopaminergic-External (p-value 1.604e-05).

				chain GO:0022900 (p-value 1.69e-30)	
ENSG00000074071	0.9026	salmon	943	mitochondrion organization GO:0007005 (p-value 4.27e-43), oxidative phosphorylation GO:0006119 (p-value 4.46e-35), mitochondrial ATP synthesis coupled electron transport GO:0042775 (p-value 1.1e-34), ATP synthesis coupled electron transport GO:0042773 (p-value 2.04e-34), purine nucleoside triphosphate metabolic process GO:0009144 (p-value 1.7e-33)	Neuron in Human brain Module (Geschwind,2010) (p-value 0.0464). Neuron_Dopaminergic-External (p-value 1.99e-06).
ENSG00000074071	0.941	royalblue	524	mitochondrion organization GO:0007005 (p-value 1.7e-36), oxidative phosphorylation GO:0006119 (p-value 2.97e-36), ATP synthesis coupled electron transport GO:0042773 (p-value 5.1e-34), respiratory electron transport chain GO:0022904 (p-value 6.87e-34), electron transport chain GO:0022900 (p-value 1.56e-33)	Neuron_Dopaminergic-External (p-value 2.247e-07).
ENSG00000074071	0.8867	greenyellow	1460	SRP-dependent cotranslational protein targeting to membrane GO:0006614 (p-value 6.34e-59), protein targeting to ER GO:0045047 (p-value 1.24e-58), cotranslational protein targeting to membrane GO:0006613 (p-value 4.72e-57), establishment of protein localization to endoplasmic reticulum GO:0072599 (p-value 5.29e-57), protein localization to endoplasmic reticulum GO:0070972 (p-value 1.48e-51)	void
ENSG00000074071	0.853	lightyellow	438	ATP synthesis coupled electron transport GO:0042773 (p-value 4.46e-32), oxidative phosphorylation GO:0006119 (p-value 8.43e-32), respiratory electron transport chain GO:0022904 (p-value 8.02e-31), mitochondrial ATP synthesis coupled electron transport GO:0042775 (p-value 9.86e-31), electron transport chain GO:0022900 (p-value 1.65e-30)	Neuron_Dopaminergic-External (p-value 0.001887).
ENSG00000074071	0.9106	darkred	1474	mitochondrion organization GO:0007005 (p-value 1.02e-42), ATP synthesis coupled electron transport GO:0042773 (p-value 1.82e-34), respiratory electron transport chain GO:0022904 (p-value 1.29e-33), mitochondrial ATP synthesis coupled electron transport GO:0042775 (p-value 1.76e-33), electron transport chain GO:0022900 (p-value 4.33e-33)	Neuron_Dopaminergic-External (p-value 3.228e-05).
ENSG00000074071	0.8896	darkturquoise	1045	mitochondrion organization GO:0007005 (p-value 9.04e-52), oxidative phosphorylation GO:0006119 (p-value 7.72e-35),	Neuron_Dopaminergic-External (p-value 2.573e-07).

				ATP synthesis coupled electron transport GO:0042773 (p-value 2.26e-34), respiratory electron transport chain GO:0022904 (p-value 2.88e-34), electron transport chain GO:0022900 (p-value 8.44e-34)	
ENSG00000074071	0.8823	midnightblue	1147	viral transcription GO:0019083 (p-value 3.22e-11), viral gene expression GO:0019080 (p-value 3.79e-11), multi-organism metabolic process GO:0044033 (p-value 6.42e-11), protein localization to endoplasmic reticulum GO:0070972 (p-value 6.1e-09), protein targeting to ER GO:0045047 (p-value 1.29e-08)	void
ENSG00000074071	0.8894	ivory	461	void	void
ENSG00000074071	0.8741	salmon2	239	void	void
ENSG00000074071	0.8338	darkseagreen4	206	void	void
ENSG00000074071	0.8784	darkmagenta	319	oxidative phosphorylation GO:0006119 (p-value 1.08e-23), ATP metabolic process GO:0046034 (p-value 1.21e-21), purine ribonucleoside triphosphate metabolic process GO:0009205 (p-value 1.45e-21), mitochondrial ATP synthesis coupled electron transport GO:0042775 (p-value 1.85e-21), ATP synthesis coupled electron transport GO:0042773 (p-value 2.42e-21)	Neuron_Dopaminergic-External (p-value 3.99e-07).
ENSG00000185499	0.5595	pink	1225	response to stress GO:0006950 (p-value 9.91e-35), immune system process GO:0002376 (p-value 9.17e-32), response to cytokine GO:0034097 (p-value 8.86e-30), cell surface receptor signaling pathway GO:0007166 (p-value 4.1e-28), response to organic substance GO:0010033 (p-value 1.12e-27)	Microglia in Human brain Module (Geschwind, 2010) (p-value 2.326e-68). Microglia (Type2) module in Cortex (p-value 7.295e-33). Microglia-External (p-value 0.01838). Endothelial-External (p-value 3.821e-08).
ENSG00000185499	0.7606	red	811	response to stress GO:0006950 (p-value 1.51e-42), defense response GO:0006952 (p-value 1.74e-39), immune system process GO:0002376 (p-value 9.78e-37), response to organic substance GO:0010033 (p-value 2.15e-33), cell surface receptor signaling pathway GO:0007166 (p-value 1.04e-32)	Microglia in Human brain Module (Geschwind, 2010) (p-value 1.649e-83). Microglia (Type2) module in Cortex (p-value 9.268e-42). Microglia (Type 1) (Geschwind, 2010) (p-value 8.268e-13). Microglia-External (p-value 0.0003156). Endothelial-External (p-value 7.882e-15).
ENSG00000185499	0.8152	pink	973	response to stress GO:0006950 (p-value 5.95e-13), regulation of response to stimulus GO:0048583 (p-value 9.29e-13), regulation of signal transduction GO:0009966 (p-value 5.63e-12), regulation of signaling GO:0023051 (p-value 9.44e-12), regulation of cell communication GO:0010646 (p-value 5.04e-11)	Astrocytes module in Cortex (p-value 4.393e-13). Microglia in Human brain Module (Geschwind, 2010) (p-value 1.388e-14). Microglia (Type2) module in Cortex (p-value 3.484e-13). Oligodendrocytes in Human brain Module (Geschwind,2010) (p-value 0.0001253). Endothelial-External (p-value 1.459e-07).

ENSG00000185499	0.4736	antiquewhite4	694	chemical synaptic transmission GO:0099536 (p-value 1.19e-12), regulation of transport GO:0051049 (p-value 5.46e-10), regulation of ion transport GO:0098662 (p-value 3.33e-09), inorganic ion transmembrane transport GO:0070838 (p-value 5.39e-09), cell-cell signaling GO:0007267 (p-value 3.66e-08)	Neuron, definite (Cahoy, 2008) (p-value 3.577e-06). Neuron, probably (Cahoy, 2008) (p-value 2.735e-14). Neuron_Pyramidal_CA1-External (p-value 3.598e-05). Neuron_Pyramidal_S1-External (p-value 0.00749). Neuron.In1-External (p-value 0.002755). Neuron.Ex1-External (p-value 0.0004293).
ENSG00000185499	0.5952	darkorange2	408	response to stress GO:0006950 (p-value 3.72e-05), immune system process GO:0002376 (p-value 0.0114), response to cytokine GO:0034097 (p-value 0.0131), dendritic cell differentiation GO:0097028 (p-value 0.0157), response to external stimulus GO:0009605 (p-value 0.025)	Astrocytes module in Cortex (p-value 0.0001686). Microglia (Type2) module in Cortex (p-value 2.449e-09).
ENSG00000185499	0.6769	paleturquoise	470	regulation of response to stimulus GO:0048583 (p-value 0.000591), regulation of signal transduction GO:0009966 (p-value 0.000629), anatomical structure morphogenesis GO:0009653 (p-value 0.000825), cell surface receptor signaling pathway GO:0007166 (p-value 0.00121), Ras protein signal transduction GO:0007265 (p-value 0.00125)	Astrocytes, highly probable (Cahoy, 2008) (p-value 0.0001791). Astrocytes module in Cortex (p-value 2.891e-12). Astrocyte-External (p-value 0.0001091).
ENSG00000185499	0.6892	brown	1592	organic acid catabolic process GO:0016054 (p-value 2.37e-23), carboxylic acid catabolic process GO:0046395 (p-value 2.37e-23), organic acid metabolic process GO:0006082 (p-value 3.19e-23), oxoacid metabolic process GO:0043436 (p-value 1.84e-22), small molecule catabolic process GO:0044282 (p-value 2.91e-22)	Astrocytes, highly probable (Cahoy, 2008) (p-value 1.003e-59). Astrocytes module in Cortex (p-value 9.24e-62). Astrocytes-Cahoy (p-value 2.306e-13). Ependymal-External (p-value 0.01246). Astrocyte-External (p-value 2.607e-36). Neuron.Ex6-External (p-value 0.02396).
ENSG00000185499	0.8212	darkslateblue	237	tissue development GO:0009888 (p-value 1.11e-05), negative regulation of cellular process GO:0048523 (p-value 2.11e-05), animal organ development GO:0048513 (p-value 4.01e-05), regulation of developmental process GO:0050793 (p-value 9.89e-05), cell differentiation GO:0030154 (p-value 0.000143)	Astrocytes, highly probable (Cahoy, 2008) (p-value 0.0003201). Astrocytes module in Cortex (p-value 7.059e-09). Microglia in Human brain Module (Geschwind, 2010) (p-value 0.0001961).
ENSG00000185499	0.6968	grey60	257	void	void
ENSG00000185499	0.6507	palevioletred2	188	void	void
ENSG00000185499	0.7407	darkturquoise	428	cell proliferation GO:0008283 (p-value 2.13e-07), regulation of cell proliferation GO:0042127 (p-value 1.77e-06), locomotion GO:0040011 (p-value 4.89e-06), localization of cell GO:0051674 (p-value 4.67e-05), cell motility GO:0048870 (p-value 4.67e-05)	Astrocytes module in Cortex (p-value 0.01064). Microglia (Type2) module in Cortex (p-value 1.647e-07).
ENSG00000185499	0.6173	green	1022	cilium assembly GO:0070925 (p-value 6.61e-38), cilium organization GO:0030031 (p-value 5.89e-29), axoneme assembly GO:0044458 (p-	Ependymal-External (p-value 3.376e-94).

				value 1.61e-27), microtubule bundle formation GO:0000226 (p-value 2.34e-24), cilium movement GO:0007018 (p-value 5.36e-21)	
ENSG00000185499	0.8111	lightcyan1	483	biological adhesion GO:0022610 (p-value 6.19e-07), cell adhesion GO:0007155 (p-value 1.33e-06), response to stress GO:0006950 (p-value 2.24e-06), regulation of response to stimulus GO:0048583 (p-value 4.59e-05), localization of cell GO:0051674 (p-value 5.3e-05)	Astrocytes, highly probable (Cahoy, 2008) (p-value 0.0139). Astrocytes module in Cortex (p-value 0.000211). Ependymal-External (p-value 0.03574).
ENSG00000196396	0.7058	pink	1225	response to stress GO:0006950 (p-value 9.91e-35), immune system process GO:0002376 (p-value 9.17e-32), response to cytokine GO:0034097 (p-value 8.86e-30), cell surface receptor signaling pathway GO:0007166 (p-value 4.1e-28), response to organic substance GO:0010033 (p-value 1.12e-27)	Microglia in Human brain Module (Geschwind, 2010) (p-value 2.326e-68). Microglia (Type2) module in Cortex (p-value 7.295e-33). Microglia-External (p-value 0.01838). Endothelial-External (p-value 3.821e-08).
ENSG00000196396	0.6685	skyblue3	490	response to unfolded protein GO:0006986 (p-value 8.88e-19), response to topologically incorrect protein GO:0035966 (p-value 1.5e-17), response to organic substance GO:0010033 (p-value 2.1e-13), protein folding GO:0006457 (p-value 2.02e-11), endoplasmic reticulum unfolded protein response GO:0030968 (p-value 5.34e-11)	Microglia-External (p-value 0.008314).
ENSG00000196396	0.6814	cyan	952	immune response GO:0006955 (p-value 2.54e-79), immune system process GO:0002376 (p-value 3.36e-79), defense response GO:0006952 (p-value 1.54e-67), regulation of immune system process GO:0045766 (p-value 4.39e-55), positive regulation of immune system process GO:0002698 (p-value 3.48e-51)	Microglia in Human brain Module (Geschwind, 2010) (p-value 5.544e-20). Microglia (Type2) module in Cortex (p-value 6.675e-34). Microglia (Type 1) (Geschwind, 2010) (p-value 4.979e-88). Microglia-External (p-value 1.268e-105).
ENSG00000196396	0.801	sienna3	789	regulation of gene expression GO:0010468 (p-value 9.02e-11), regulation of RNA metabolic process GO:0051252 (p-value 6.55e-10), RNA biosynthetic process GO:0032774 (p-value 7.16e-10), nucleic acid-templated transcription GO:0097659 (p-value 2.34e-09), regulation of nucleobase-containing compound metabolic process GO:0019219 (p-value 2.8e-09)	void
ENSG00000196396	0.8399	ivory	452	response to stress GO:0006950 (p-value 7.99e-10), negative regulation of cellular process GO:0048523 (p-value 2.17e-09), regulation of gene expression GO:0010468 (p-value 4.73e-09), negative regulation of macromolecule metabolic process GO:0010605 (p-value 1.39e-08), negative regulation of metabolic	void

				process GO:0009892 (p-value 4.38e-08)	
ENSG00000196396	0.7924	lightyellow	510	response to cytokine GO:0034097 (p-value 7.16e-29), response to organic substance GO:0010033 (p-value 6.15e-22), cellular response to cytokine stimulus GO:0071345 (p-value 2e-20), cell death GO:0008219 (p-value 3.03e-20), regulation of apoptotic process GO:0042981 (p-value 1.16e-19)	Microglia in Human brain Module (Geschwind, 2010) (p-value 2.078e-53). Microglia (Type2) module in Cortex (p-value 2.272e-20). Endothelial-External (p-value 0.00092).
ENSG00000196396	0.7174	tan	1983	histone modification GO:0016570 (p-value 0.000544), covalent chromatin modification GO:0016569 (p-value 0.00055), peptidyl-lysine modification GO:0018205 (p-value 0.00433), mRNA-containing ribonucleoprotein complex export from nucleus GO:0071427 (p-value 0.00575), mRNA export from nucleus GO:0006406 (p-value 0.00575)	void
ENSG00000196396	0.7463	darkmagenta	484	positive regulation of protein catabolic process GO:0045732 (p-value 3.77e-07), regulation of protein catabolic process GO:0042176 (p-value 5.69e-07), cellular response to topologically incorrect protein GO:0035967 (p-value 6.46e-07), response to endoplasmic reticulum stress GO:0034976 (p-value 1.03e-06), protein catabolic process GO:0030163 (p-value 3.04e-06)	void
ENSG00000196396	0.697	brown4	257	RNA processing GO:0006396 (p-value 1.72e-05), mRNA metabolic process GO:0016071 (p-value 2.11e-05), regulation of gene expression GO:0010468 (p-value 2.27e-05), RNA biosynthetic process GO:0032774 (p-value 3.35e-05), transcription, DNA-templated GO:0006351 (p-value 4.08e-05)	void
ENSG00000196396	0.74	ivory	260	regulation of inflammatory response GO:0050727 (p-value 0.0222)	void
ENSG00000196396	0.7558	mediumpurple3	541	mRNA metabolic process GO:0016071 (p-value 4.84e-13), mRNA processing GO:0006397 (p-value 7.08e-12), regulation of gene expression GO:0010468 (p-value 2.52e-11), RNA processing GO:0006396 (p-value 5.69e-11), RNA splicing, via transesterification reactions GO:0000375 (p-value 6.26e-11)	void
ENSG00000196396	0.7153	antiquewhite4	339	void	Oligodendrocytes in Human brain Module (Geschwind,2010) (p-value 6.053e-07). Oligodendrocytes in Cortex (p-value 0.008201). Oligodendrocyte-External (p-value 5.488e-10).
ENSG00000196396	0.7383	pink	829	response to organic substance GO:0010033 (p-value 1.44e-34),	Astrocytes, highly probable (Cahoy, 2008) (p-value 0.001327). Astrocytes module in

				response to stress GO:0006950 (p-value 5.04e-34), cellular response to chemical stimulus GO:0070887 (p-value 6.35e-31), cellular response to organic substance GO:0071310 (p-value 1.6e-30), response to cytokine GO:0034097 (p-value 2.71e-28)	Cortex (p-value 0.003138). Microglia in Human brain Module (Geschwind, 2010) (p-value 1.779e-68). Microglia (Type2) module in Cortex (p-value 2.723e-37). Microglia-External (p-value 0.04953). Neutrophils-External (p-value 0.0373).
